# Supplementary material for: Computationally predicting clinical drug combination efficacy with cancer cell line screens and independent drug action
Source: Nat Commun. 2020 Nov 17;11:5848. doi: 10.1038/s41467-020-19563-6 (PMC7673995; doi:10.1038/s41467-020-19563-6)
Supplement: Supplementary file 1 — Supplementary Information [file 41467_2020_19563_MOESM1_ESM.pdf]

Supplemental Text for "Computationally predicting clinical drug combination efficacy with cancer cell line screens and independent drug action"

Alexander Ling and R. Stephanie Huang

SUPPLEMENTAL TEXT DESCRIPTION

This file contains supplemental figures, tables, results, discussion, and references that relate to the findings of the main text but could not be included there due to a lack of space. In general, the text in this document simply expands on findings and discussions from the main text without introducing entirely new findings or discussion topics.

SUPPLEMENTAL FIGURES

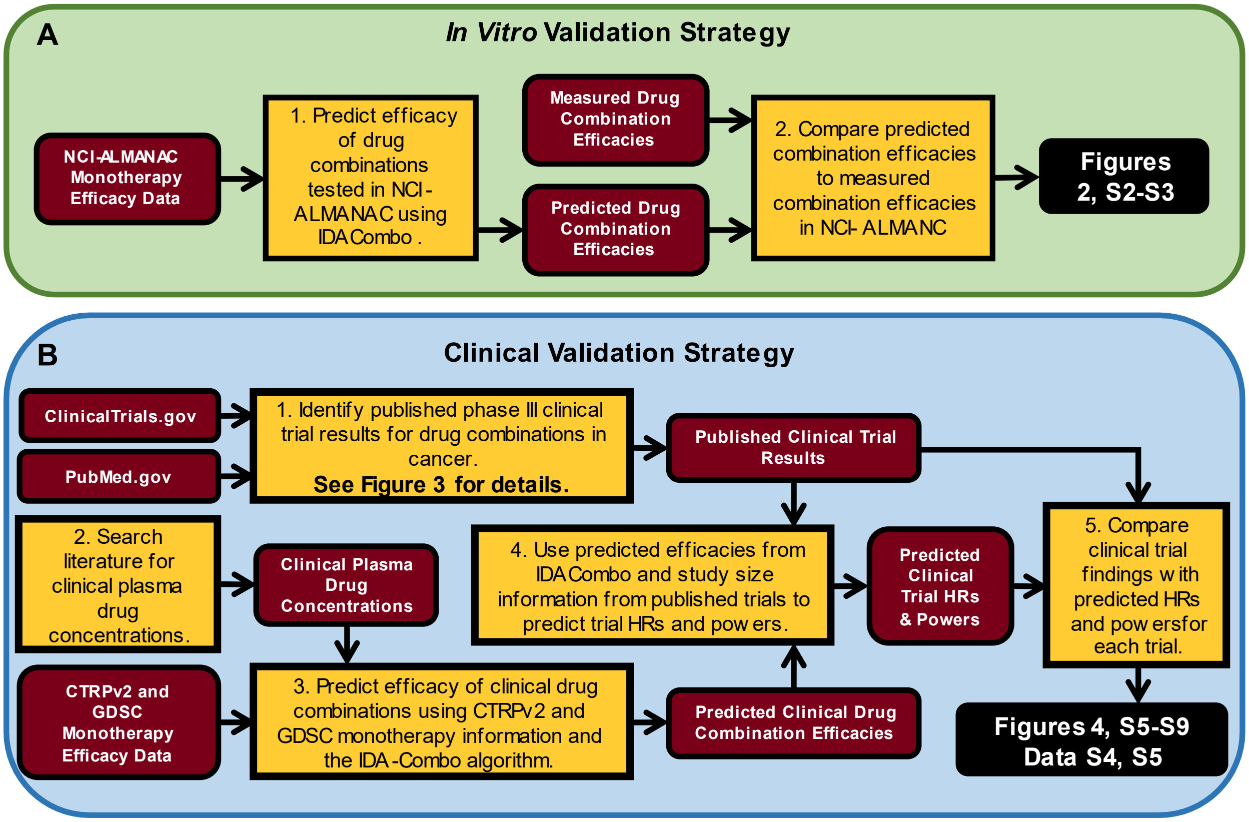

Figure S1. Pipelines to validate IDACombo predictions both in vitro and in clinical trial data. A) In vitro validation strategy. Monotherapy data from NCI-ALMANAC is used to predict drug combination efficacies, and these efficacies are compared to the measured combination efficacies that are also in NCI-ALMANAC. **B)** Clinical trials are systematically identified using ClinicalTrials.gov and PubMed.gov, and efficacy predictions are made for each treatment in each trial using clinical drug concentrations and monotherapy cell line data from CTRPv2 and/or GDSC. These predictions are used to estimate hazard ratios (HRs) and powers for each trial, and these HRs and powers are compared to reported clinical trial outcomes.

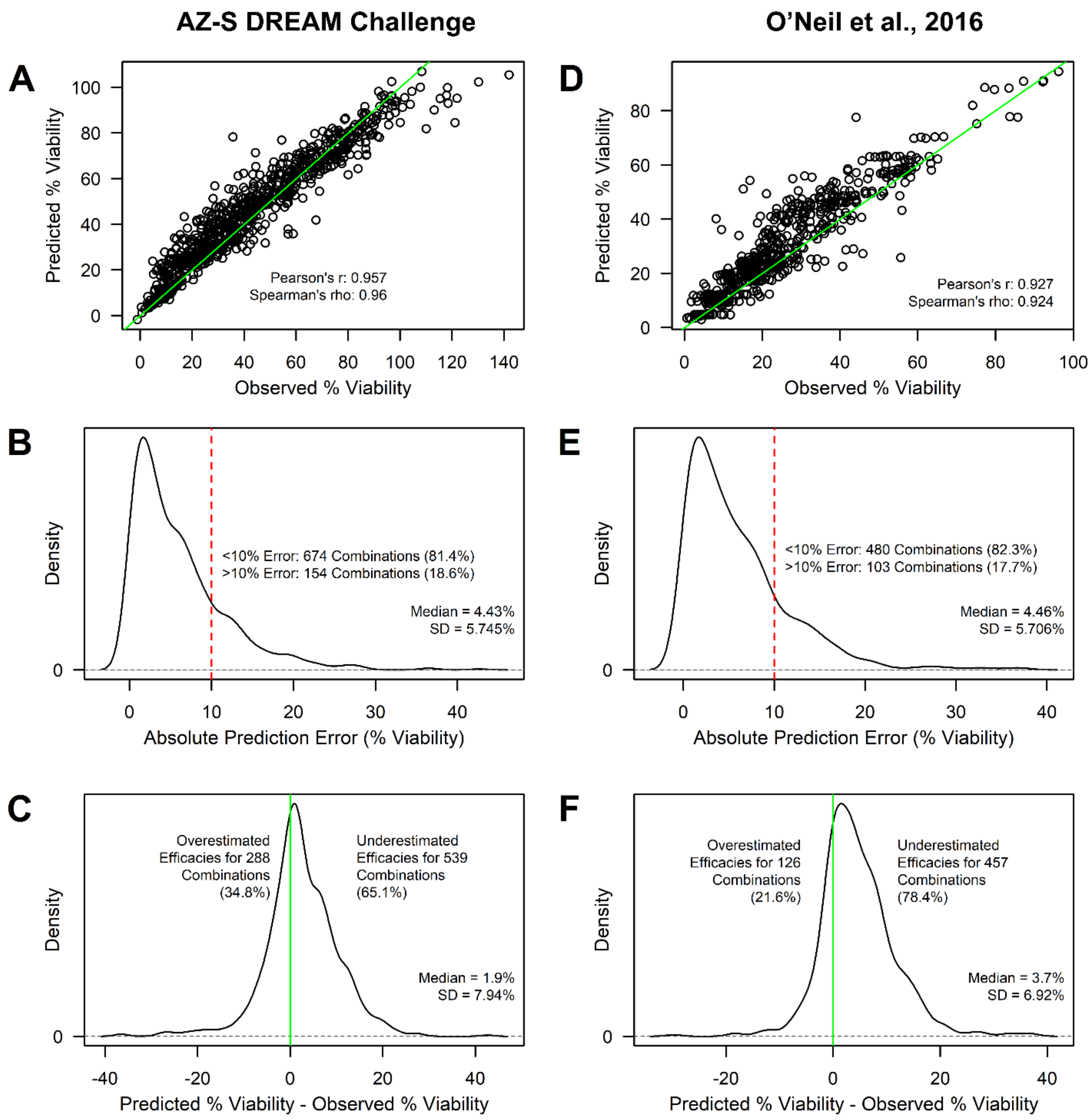

Figure S2. Agreement between predicted and observed combination viabilities in the AstraZeneca-Sanger DREAM Challenge and O'Neil et al., 2016 drug combination datasets. A-C) Results using the AstraZeneca-Sanger DREAM challenge (AZ-S DREAM) drug combination dataset. **D-F)** Results using the O'Neil et al., 2016 drug combination dataset. **A&D)** Scatterplot showing high correlation between predicted average percent viability and experimentally observed average percent viability for each drug combination in the dataset. Predictions were made using monotherapy data from the dataset. The green line is a reference diagonal with slope = 1 and intercept = 0. Note that predictions were only made for the maximum concentration tested for each drug. **B&E)** Density plot showing that the absolute values of the differences between the predicted percent viabilities and the observed percent viabilities for each drug combination are generally below 10%, with >50% of drug combinations having an absolute prediction error below 5%. The red line marks a difference of ±10% viability between predicted and observed values. **C&F)** Density plot showing that the differences between the predicted percent viabilities and the observed percent viabilities for each drug combination have a slight tendency towards being positive—indicating that IDA-Combo underestimates efficacy more often than it overestimates efficacies. Source data are provided with this paper.

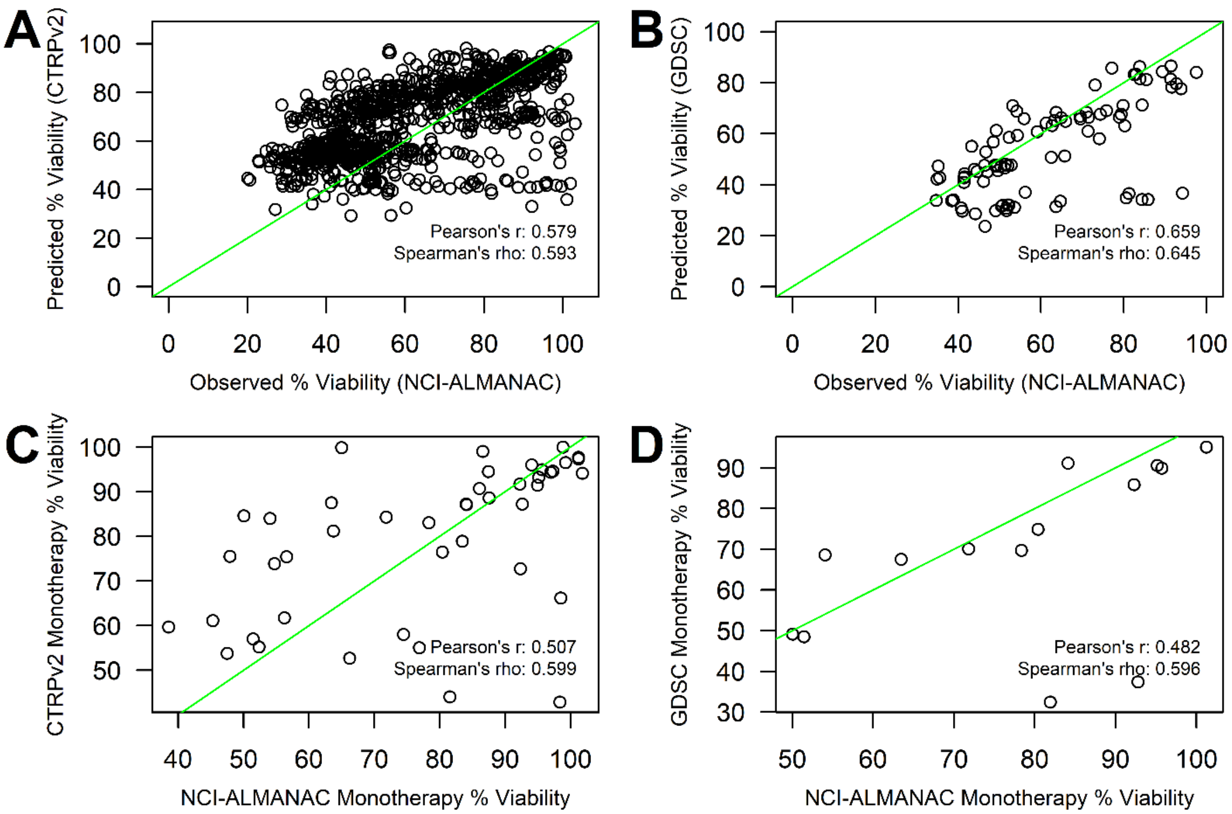

Figure S3. Agreement between drug combination efficacy predictions made with CTRPv2 or GDSC and measured efficacies in NCI-ALMANAC A-B) Scatterplots showing the correlation between drug combination mean % viabilities predicted with IDACombo and **(A)** CTRPv2 or **(B)** GDSC monotherapy data vs measured mean % viabilities for those combinations in NCI-ALMANAC. The green line is a reference diagonal with slope = 1 and intercept = 0. Note that CTRPv2 and GDSC predictions were made using all available cell lines for each combination in the dataset, so the predicted and measured mean % viabilities were produced with different cell line sets. Also note that predictions were only made for the maximum tested NCI-ALMANAC concentrations for each drug and that overlapping combinations were excluded if the concentration tested in NCI-ALMANAC exceeded the maximum tested concentration in CTRPv2 (for **A)** or GDSC (for **B**) for any drug in the combination. **C-D)** Mean monotherapy % viabilities for each drug included in at least one of the drug combinations plotted in **A** or **B** for **C** and **D** respectively. Monotherapy viabilities are plotted for **C)** CTRPv2 vs. NCI-ALMANAC and **D)** GDSC vs NCI-ALMANAC with average viabilities being calculated for all available cell lines in each dataset for each drug. The green line is a reference diagonal with slope = 1 and intercept = 0. Note that measured viability averages are at the maximum tested NCI-ALMANAC concentrations used for that drug in each combination the drug was included in. If the maximum concentration for a drug differed between different combinations involving that drug in NCI-ALMANAC, the most commonly used maximum concentration was selected for plotting in panels **C** and **D**. Source data are provided with this paper.

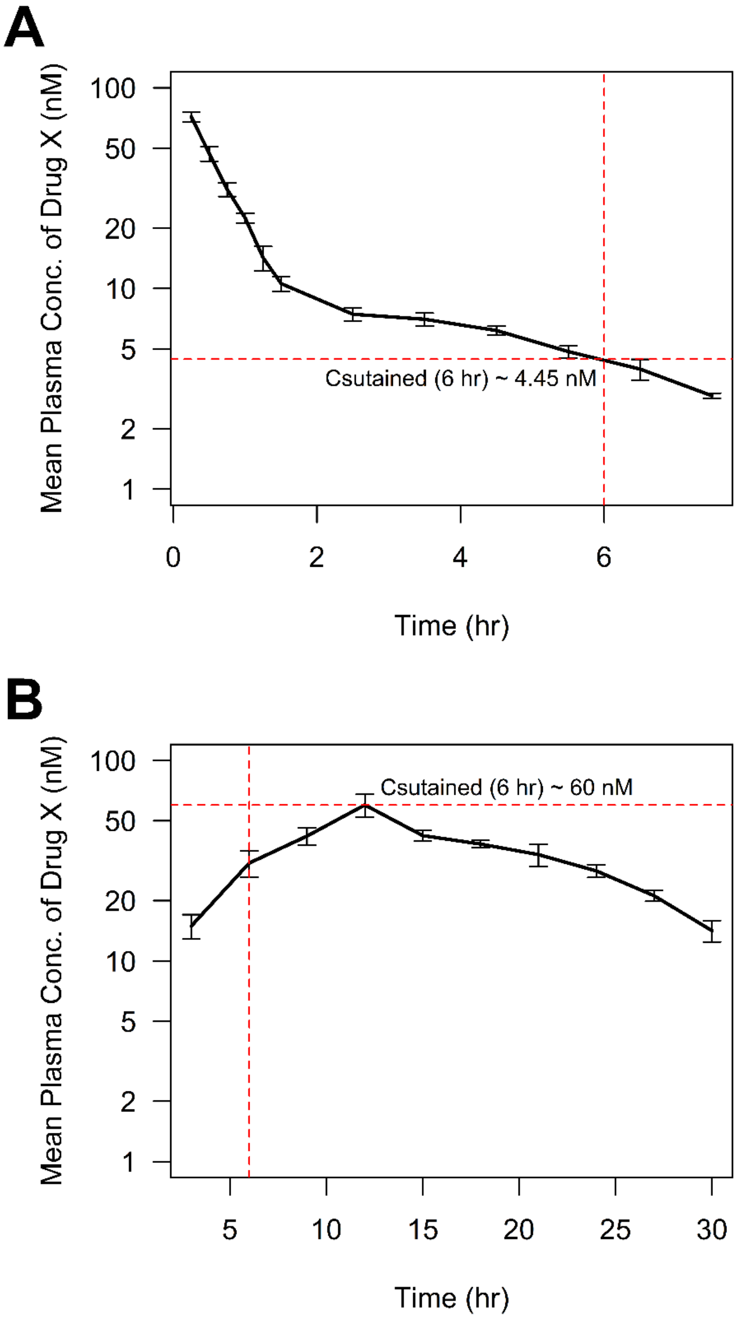

Figure S4. Calculating Csustained,6hr from clinical plasma concentration curves. This figure gives two hypothetical examples to illustrate how Csustained is calculated from plasma concentration curves identified in phase I or II clinical trials. **A)** When mean plasma drug concentrations constantly decrease following administration of a drug, Csustained,6hr is simply the mean plasma concentration at 6 hours after drug administration. **B)** When mean plasma drug concentrations continue rising for more than 6 hours following administration of a drug, Csustained,6hr is the maximum plasma concentration achieved at least 6 hours after drug administration. Error bars represent mean ± standard error.

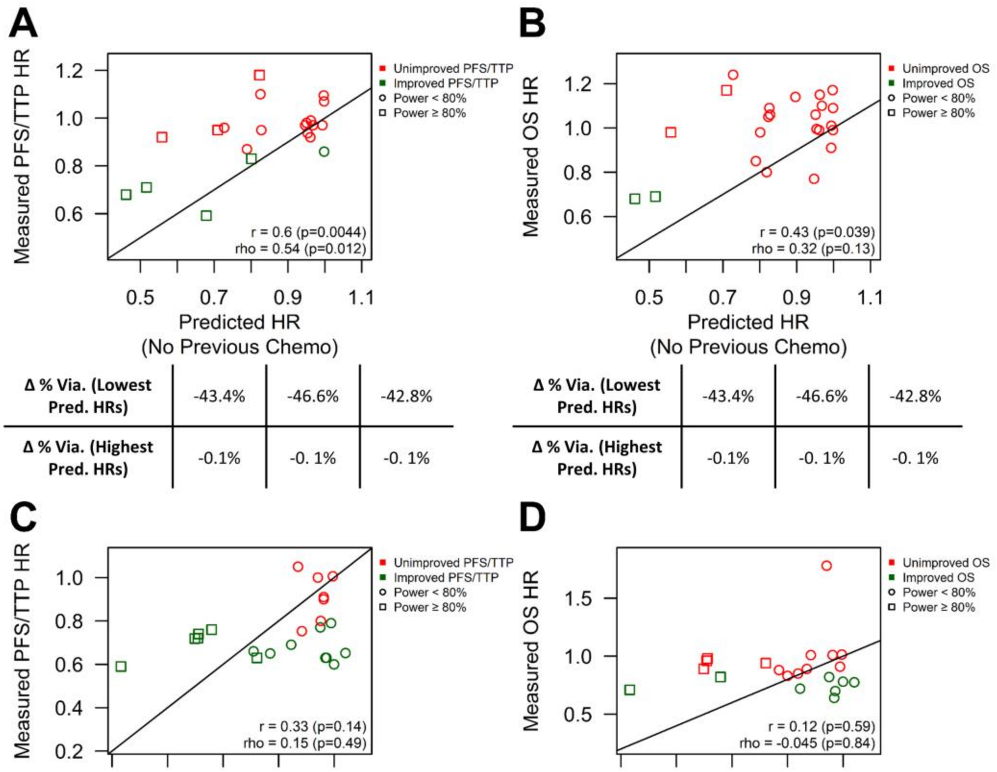

A

**Δ % Via. (Lowest Pred. HRs)**	-43.4%	-46.6%	-42.8%
**Δ % Via. (Highest Pred. HRs)**	-0.1%	-0. 1%	-0. 1%

B

**Δ % Via. (Lowest Pred. HRs)**	-43.4%	-46.6%	-42.8%
**Δ % Via. (Highest Pred. HRs)**	-0.1%	-0. 1%	-0. 1%

C

**Δ % Via. (Lowest Pred. HRs)**	-42.2%	-43.9%	-48.0%
**Δ % Via. (Highest Pred. HRs)**	2.2%	0. 0%	-0.2%

D

**Δ % Via. (Lowest Pred. HRs)**	-42.2%	-43.9%	-48.0%
**Δ % Via. (Highest Pred. HRs)**	2.2%	0.0%	-0.2%

Figure S5. Predicted vs measured hazard ratios for clinical validation analysis. This figure shows how hazard ratios (HRs) predicted with IDACombo (x-axes) compare to HRs reported by the clinical trials selected for the clinical trial validation analysis (y-axes). Note that, while this figure includes largely the same set of trials used in Figure 4 in the main text, some of those trials are not included in this figure because they did not report HRs. Red points represent trials which did not report a HR that was statistically less than 1, while green points represent trials that did report a HR that was statistically less than 1. Circles represent trials where the power predicted by IDACombo for that trial was <80%, while squares represent trials where the predicted power was ≥80%. Pearson's r and Spearman's rho are reported alongside two-sided p-values for whether or not the measured correlation is significantly different from 0. **A)** Measured PFS/TTP HRs vs predicted HR in clinical trials where patients had not received chemotherapy prior to trial entry. **B)** Measured OS HRs vs predicted HR in clinical trials where patients had not received chemotherapy prior to trial entry. **C)** Measured PFS/TTP HRs vs predicted HR in clinical trials where patients had received chemotherapy prior to trial entry. **D)** Measured OS HRs vs predicted HR in clinical trials where patients had received chemotherapy prior to trial entry. Note that further information for these trials and IDACombo's predictions for them is included in Supplementary Data 3. The tables below each plot indicate the change in predicted mean viability for the experimental therapy vs. the control therapy for the three highest predicted HRs and the three lowest predicted HRs from each panel (negative values indicate experimental therapy has lower predicted viability than control therapy). Source data are provided with this paper.

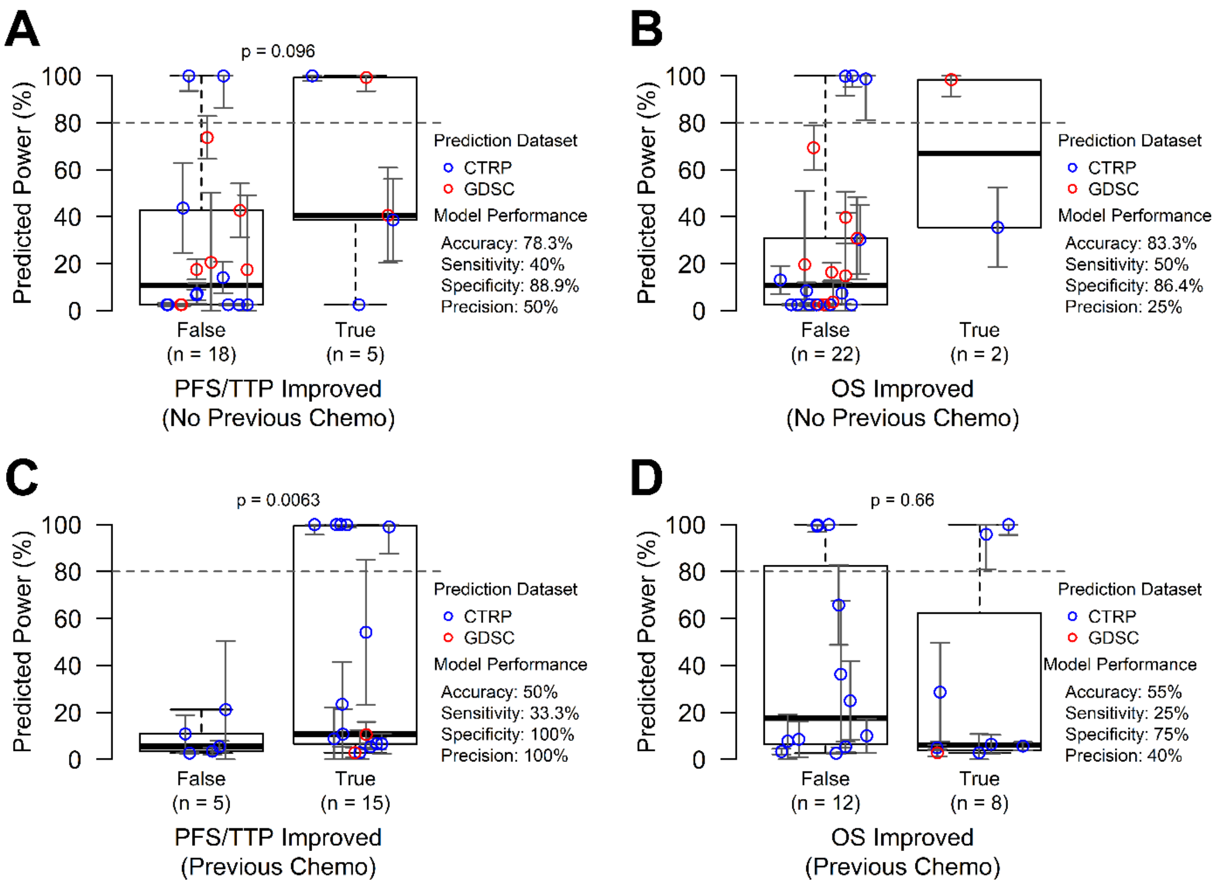

Figure S6. Using only cancer-specific cell lines does not improve model performance for clinical trial power predictions. Identical to Figure 4, except that predictions were made for each trial using sets of cell lines specific to the cancer type being studied in each trial. **A)** Predicted power of each trial in previously untreated patients to detect a significant improvement in PFS/TTP at an alpha of 0.05 versus whether or not the study actually detected a significant improvement in PFS/TTP. **B)** Predicted power of each study in previously untreated patients to detect a significant improvement in OS at an alpha of 0.05 versus whether or not the study actually detected a significant improvement in OS. **C)** Predicted power of each trial in previously treated patients to detect a significant improvement in PFS/TTP at an alpha of 0.05 versus whether or not the study actually detected a significant improvement in PFS/TTP. **D)** Predicted power of each study in previously treated patients to detect a significant improvement in OS at an alpha of 0.05 versus whether or not the study actually detected a significant improvement in OS. Error bars for each plotted clinical trial power represent mean estimated power ± standard error (bounded between 0 and 100% power). P values were calculated using one-tailed t-tests. Blue circles indicate predictions made using the CTRP dataset, and red circles indicate predictions made using the GDSC dataset. Boxplots are plotted so that the lower and upper whiskers indicate the extreme lower and upper values respectively, the box boundaries indicate the first and third quartiles, and the center line indicates the median. Source data are provided with this paper.

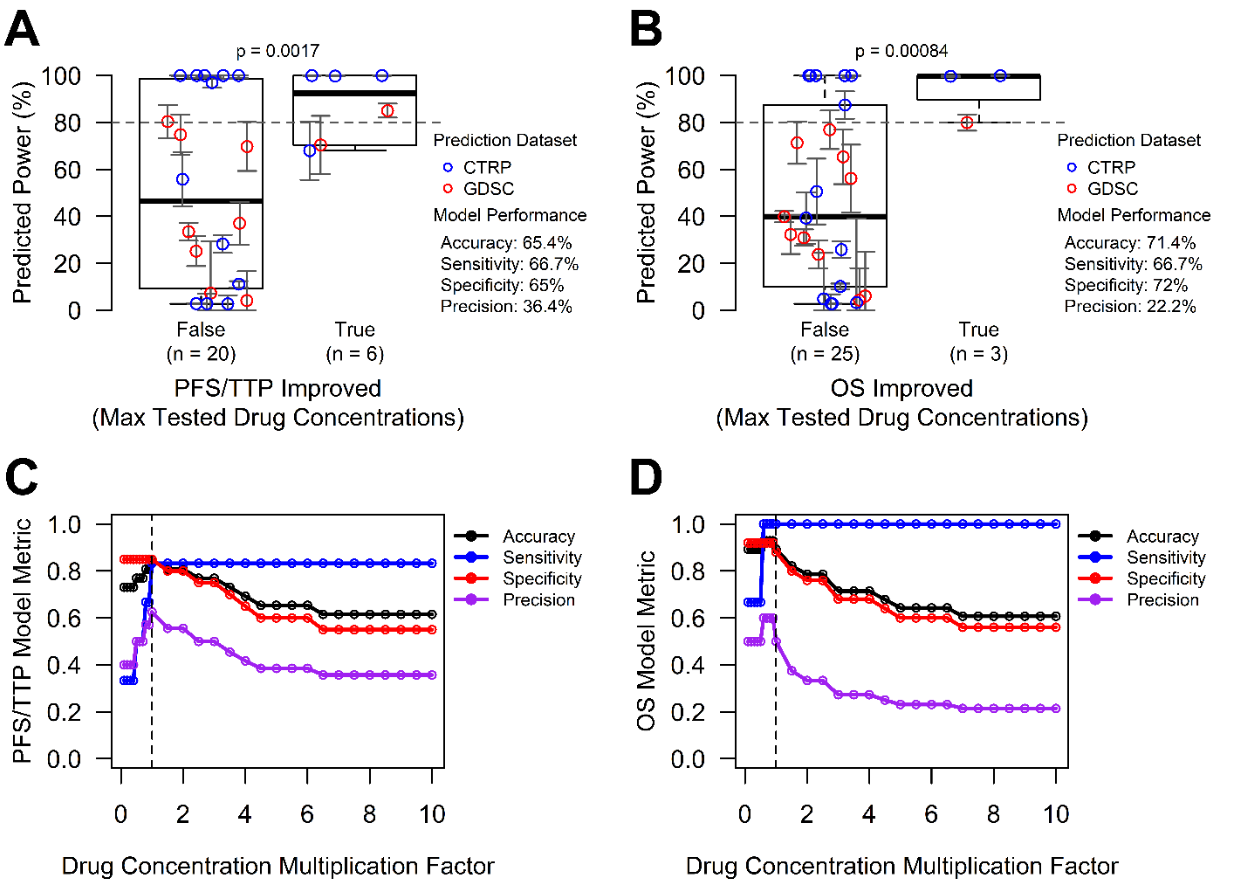

Figure S7. Clinical power predictions are dose-dependent. A&B) Similar to Figure 4A and 4B, all available cell lines were used to create predictions of study power for trials in chemo-naïve patients and compared to whether or not the trials saw a statistically significant improvement in PFS/TTP **(A)** or OS **(B)**. In this figure, however, maximum tested concentrations were used for each drug instead of Csustained concentrations. Notably, these predictions with the maximum tested concentration of each drug results in much poorer model performance than the Csustained predictions in Figure 4. **C&D)** In an effort to determine how sensitive our method is to dose perturbation, power predictions were made for each trial in chemo-naïve patients using Csustained drug concentrations which have been multiplied by a multiplication factor between 0.1 and 10. Model performance metrics for PFS/TTP **(C)** or OS **(D)** were then calculated using predictions from each dose multiplication factor, and those metrics are plotted here. Error bars for each plotted clinical trial power represent mean estimated power ± standard error (bounded between 0 and 100% power). P values in **A** and **B** were calculated using one-tailed t-tests. Blue circles indicate predictions made using the CTRP dataset, and red circles indicate predictions made using the GDSC dataset. Boxplots are plotted so that the lower and upper whiskers indicate the extreme lower and upper values respectively, the box boundaries indicate the first and third quartiles, and the center line indicates the median. Source data are provided with this paper.

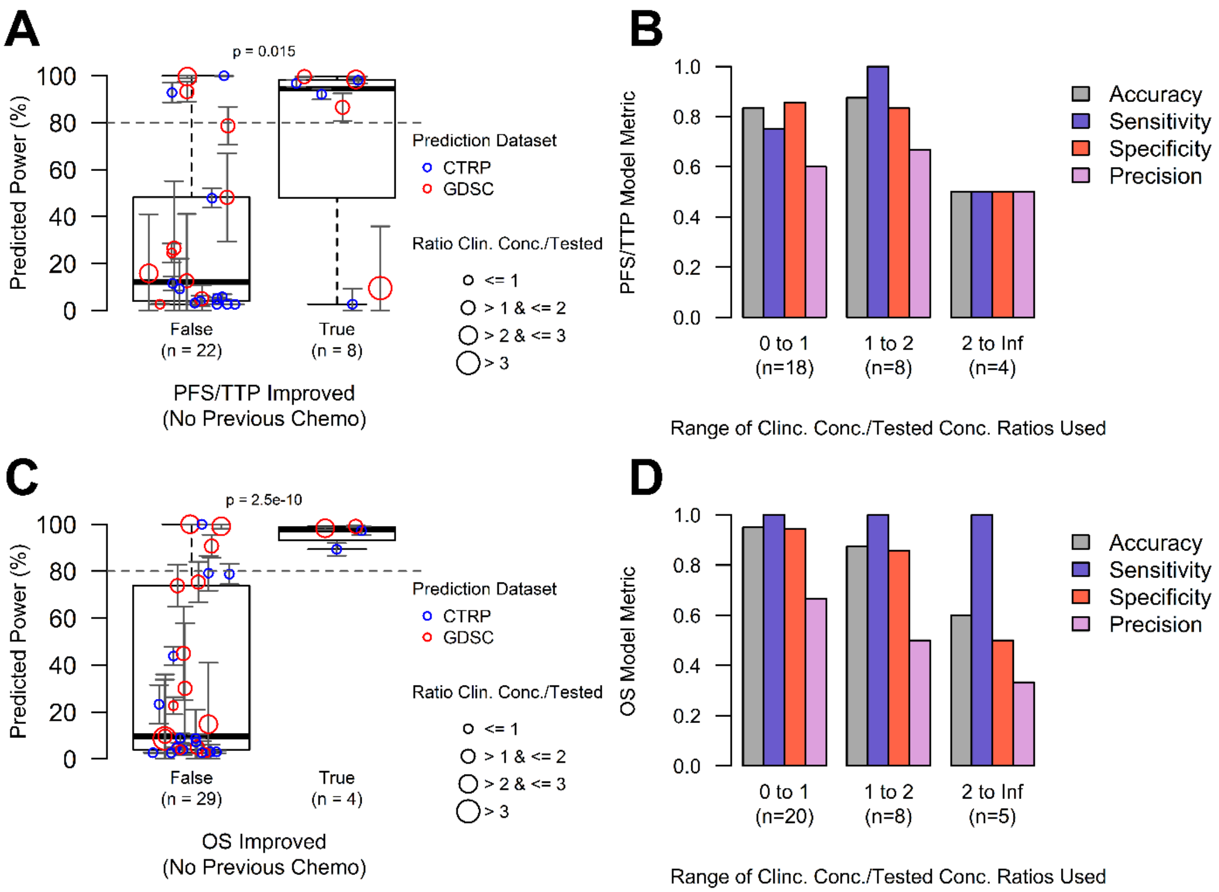

Figure S8. IDACombo predictions become less accurate when made using drug concentrations beyond the tested monotherapy concentration range. A) Similar to Figure 4A, this plot shows predicted clinical trial powers for PFS/TTP in trials with chemo-naïve patients, separated based on whether or not the trial actually observed a statistical improvement in PFS/TTP with the test treatment. Trial points are sized according to the maximum ratio of the Csustained concentrations used for the drugs in the trial to the maximum tested concentrations of those drugs in CTRPv2 or GDSC. Ratios above 1 indicate that the Csustained concentration is higher than the maximum available concentration in CTRPv2 or GDSC. Notably, most of the incorrectly classified trails have ratios > 1 and most of the correctly classified trials have ratios < 1. **B)** Barplot showing PFS/TTP model performance for trials with chemo-naïve patients that fall within three different ranges of ratios of drug Csustained concentration to tested concentration in CTRPv2 or GDSC. Notably, trials with a Csustained/tested concentration ratio > 2 are predicted much more poorly than trials with a ratio between 0 and 1 or with a ratio between 1 and 2. **C)** Same as **A**, except for OS in trials with chemo-naïve patients. **D)** Same as **B**, except for OS in trials with chemo-naïve patients. Error bars for each plotted clinical trial power represent mean estimated power ± standard error (bounded between 0 and 100% power). P values in **A** and **C** were calculated using one-tailed t-tests. Blue circles indicate predictions made using the CTRP dataset, and red circles indicate predictions made using the GDSC dataset. Boxplots are plotted so that the lower and upper whiskers indicate the extreme lower and upper values respectively, the box boundaries indicate the first and third quartiles, and the center line indicates the median. Source data are provided with this paper.

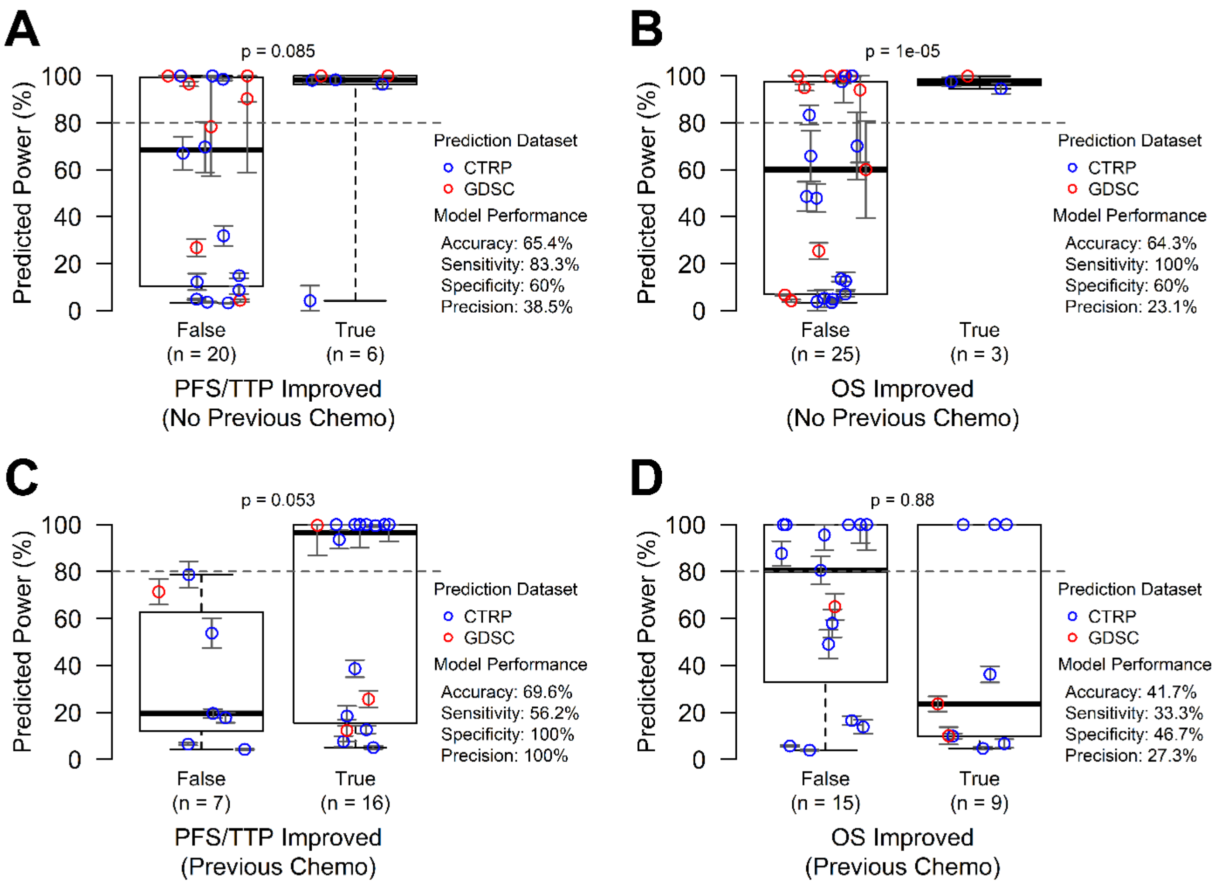

Figure S9. Predictions made using Bliss independence are less accurate than those made with independent drug action. Power predictions were made for the clinical trials shown in Figure 4, but using the Bliss independence model rather than the IDA model. In general, when compared to the IDA predictions in Figure 4, Bliss Independence inflates estimated powers, leading to decreased precision, specificity, and accuracy while providing marginal improvements in sensitivity. Error bars for each plotted clinical trial power represent mean estimated power ± standard error (bounded between 0 and 100% power). P values were calculated using one-tailed t-tests. Blue circles indicate predictions made using the CTRP dataset, and red circles indicate predictions made using the GDSC dataset. Boxplots are plotted so that the lower and upper whiskers indicate the extreme lower and upper values respectively, the box boundaries indicate the first and third quartiles, and the center line indicates the median. Source data are provided with this paper.

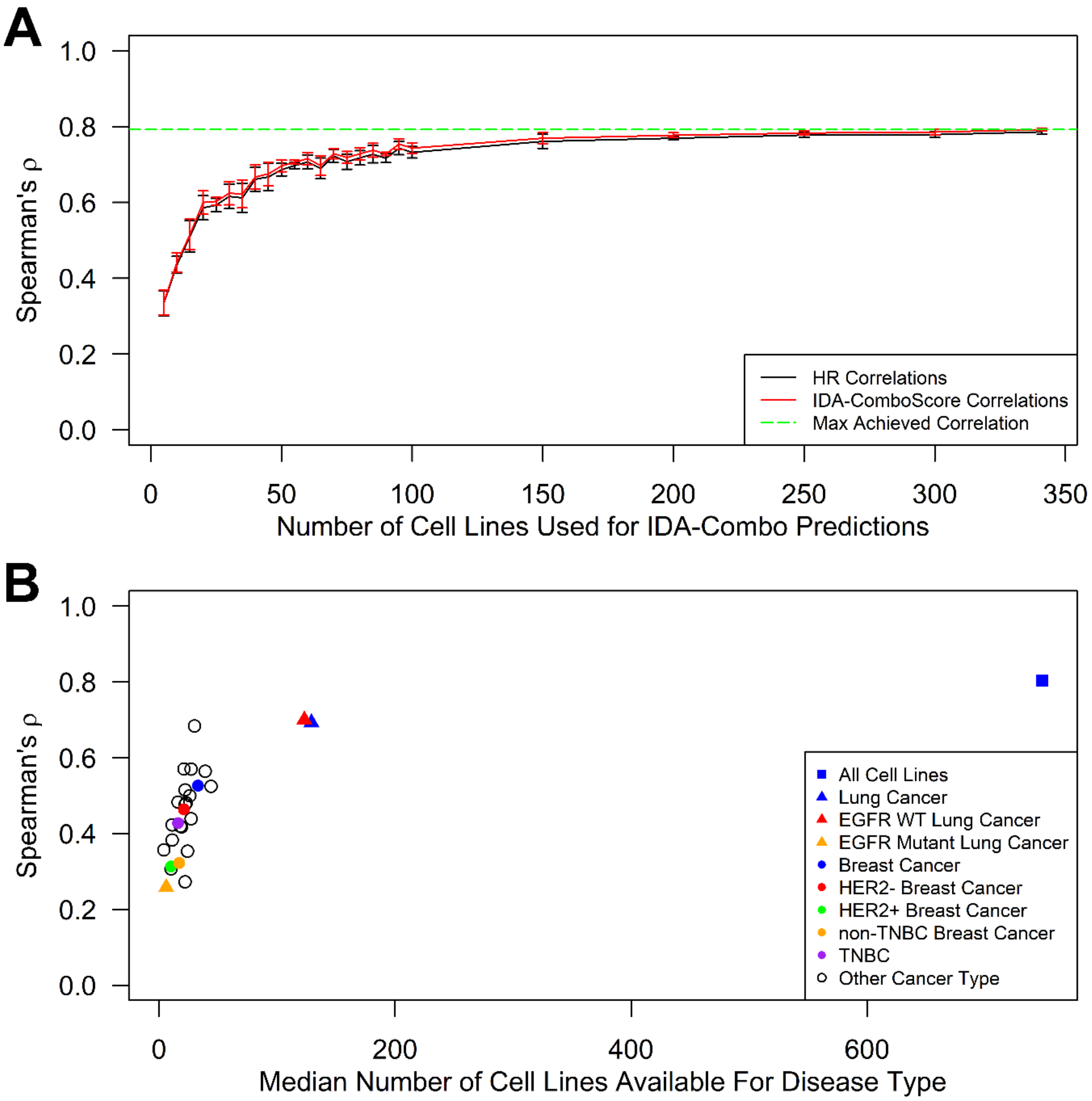

Figure S10. IDAcomboscore agreement between CTRPv2 and GDSC is affected by the number of cell lines available to make predictions with. In an effort to determine how many cell lines are required to estimate drug combination efficacy, IDAcomboscore correlations between CTRPv2 and GDSC are plotted versus the number of cell lines used to make those predictions. **A)** IDA-comboscore predictions were made using randomly sampled sets of cell lines of varying sizes. Sampling was performed three times for each number of cell lines and the mean of each triplicate is plotted with error bars representing the standard deviation of the triplicate correlation measurements. Notably, agreement between CTRPv2 and GDSC decreases rapidly as the number of cell lines is reduced below 50. **B)** Correlations are plotted for predictions made using cancer-specific cell lines. Note that the x-axis denotes the median number of cell lines available for that cancer type for each drug combination, as the number of cell lines available for each cancer type varies from drug to drug. Subsets of breast cancer and lung cancer are highlighted in the plot. Note that for both panels **A** and **B**, correlations were only calculated for drug combinations that used drugs for which their clinical doses were available in both CTRP and GDSC so as to avoid calculating correlations between predictions made with different drug concentrations between the two datasets. Source data are provided with this paper.

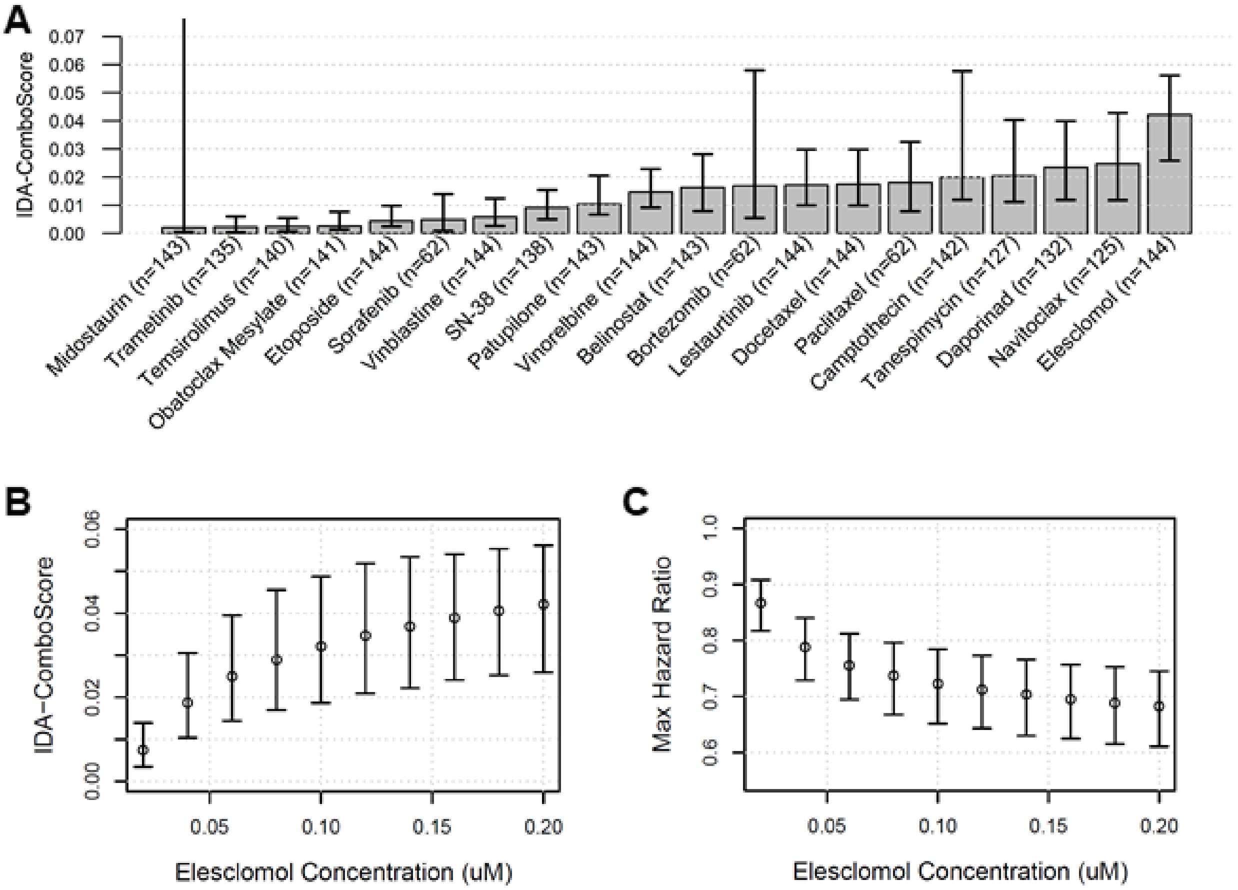

Figure S11. IDACombo predicts that elesclomol will efficaciously combine with cisplatin+gemcitabine in EGFR WT lung cancer. A) IDAcomboscores were calculated for the addition of late-stage clinical drugs in GDSC at their Csustained concentrations to the control treatment combination of Cisplatin (6.44μM) + Gemcitabine (1.14μM) in EGFR WT lung cancer. The number of cell lines available to generate predictions for combinations with each additional drug are provided in parentheses in the x-axis labels. Only the top 20 IDAcomboscores are plotted here. **B)** Predicted IDAcomboscores for the addition of elesclomol to the combination of Cisplatin (6.44μM) + Gemcitabine (1.14μM) across a range of concentrations of elesclomol in EGFR WT lung cancer. **C)** Maximum predicted hazard ratios for the addition of elesclomol to combination of Cisplatin (6.44μM) + Gemcitabine (1.14μM) in EGFR WT lung cancer across a range of concentrations of elesclomol. Maximum hazard ratio is defined as the higher hazard ratio (i.e. the hazard ratio that indicates less efficacy improvement from the test treatment vs the control treatment) of either: 1. elsclomol+cisplatin+gemcitabine vs cisplatin+gemcitabine or 2. elesclomol+cisplatin+gemcitabine vs elesclomol monotherapy. **A-C)** Error bars represent mean ± 95% confidence interval as estimated using Monte Carlo Simulations (see Online Methods). Source data are provided with this paper.

SUPPLEMENTAL TABLES

Table S1. R packages used in the analyses performed in this paper.

Package Name	Package Version	Package Citation	Package WebLink
car	2.1.5	Fox and Weisberg, 2011	https://CRAN.R-project.org/package=car
ComplexHeatmap	1.14.0	Gu et al., 2016	https://bioconductor.org/packages/release/bioc/html/ComplexHeatmap.html
drc	3.0.1	Ritz et al., 2015	https://CRAN.R-project.org/package=drc
IDACombo	1.0.2	This paper.	https://github.com/Alexander-Ling/IDACombo
openxlsx	4.1.4	Schauberger and Walker, 2019	https://CRAN.R-project.org/package=openxlsx
parallel	3.4.2	R Core Team, 2017	Created by the R Core team and included in R since R version 2.14.0.
pbapply	1.3.3	Solymos and Zawadzki, 2017	https://CRAN.R-project.org/package=pbapply
powerSurvEpi	0.0.9	Qiu et al., 2015	https://CRAN.R-project.org/package=powerSurvEpi
precrec	0.9.1	Saito and Rehmsmeier, 2017	https://CRAN.R-project.org/package=precrec
progress	1.1.2	Csárdi and FitzJohn, 2016	https://CRAN.R-project.org/package=progress
RColorBrewer	1.1.2	Neuwirth, 2014	https://CRAN.R-project.org/package=RColorBrewer
readr	1.1.1	Wickham et al., 2017	https://CRAN.R-project.org/package=readr
readxl	1.0.0	Wickham and Bryan, 2017	https://CRAN.R-project.org/package=readxl
rgl	0.98.1	Adler et al., 2017	https://CRAN.R-project.org/package=rgl
rvest	0.3.2	Wickham, 2016	https://CRAN.R-project.org/package=rvest
sandwich	2.4.0	Zeileis, 2004, 2006	https://CRAN.R-project.org/package=sandwich
xlsx	0.5.7	Dragulescu, 2014	https://CRAN.R-project.org/package=xlsx

SUPPLEMENTAL RESULTS

Clinical Trial Validation

Identifying clinical drug concentrations for clinical validation of IDACombo

As mentioned in the main text, we searched published phase I and II clinical trials to identify clinical plasma concentrations for each drug at the administered doses used in each trial in our clinical trial validation analysis. Since maximum plasma concentrations (Cmax) are extremely transient for some drugs, especially those administered via IV bolus, we decided to use the maximum plasma concentrations achieved at least 6 hours after drug administration (a metric we termed Csustained,6hr) as our concentrations for IDACombo predictions. Figure S4A illustrates how Csustained is calculated for drugs with constantly decreasing plasma concentrations over time, and Figure S4B illustrates how Csustained is calculated for drugs with increasing plasma concentrations beyond 6 hours. A more detailed description of this metric and why it was chosen is included in the Online Methods. Csustained values for each drug in the clinical trial analysis, along with the citations used to determine them, are included in Supplementary Data 4.

Misclassified trials in Figure 4A: PFS/TTP powers in first-line therapy trials

The first false positive in Figure 4A tested the addition of vinorelbine to gemcitabine in non-small cell lung cancer (NSC lung cancer)[1]. Notably, the National Comprehensive Cancer Network (NCCN) currently classifies vinorelbine + gemcitabine as a category 1 therapy useful in certain circumstances for the first-line treatment of advanced NSC lung cancer[2], indicating that the predicted utility of this combination may not be entirely inappropriate. Furthermore, this trial was unusual in our clinical validation dataset in that it only enrolled elderly patients (≥70 years old), most of whom had multiple comorbidities and non-zero ECOG scores, and death from unknown causes or losing patients to follow up was considered as progression in this study.

The other two false positives in Figure 4A were ovarian cancer trials that tested: 1) the addition of paclitaxel to carboplatin[3] and 2) the addition of gemcitabine to paclitaxel + carboplatin[4]. While NCCN guidelines do not recommend paclitaxel + carboplatin + gemcitabine for ovarian cancer[5], paclitaxel + carboplatin is considered the backbone of first-line therapy for ovarian cancer[6]. That said, there is reason to doubt IDACombo's predictions for these treatments, because dimethyl sulfoxide (DMSO) was used as the solvent for drug testing in CTRPv2, and DMSO is known to inactivate platinum complexes[7]. Indeed, carboplatin monotherapy produces an average viability of 97% in CTRPv2 (Supplementary Data 3), suggesting that the drug may be inactivated in the dataset and, therefore, is not being properly accounted for in the control therapies for these trials.

The false negative in Figure 4A was also an ovarian cancer trial, this time testing the addition of nintedanib to paclitaxel + carboplatin[8]. Since carboplatin inactivation in this case would have increased the predicted benefit of nintedanib, it cannot be the cause of this misclassification. While it is possible that this case represents a case of drug additivity/synergy, we believe the fact that three out of four misclassified trials are ovarian cancer trials suggests that the pan-cancer set of cell lines used to generate these predictions may perform poorly when making predictions for ovarian cancer trials. It is also worth noting that, while this study did detect a statistically significant improvement in PFS, the study's authors note that the

improvement is “of limited clinical relevance” and that the study’s results “do not support a role for nintedanib in ovarian cancer”[8].

Clinical IDACombo predictions with cancer type/subtype specific sets of cell lines

As mentioned in the main text, we performed analyses to evaluate the suitability of IDACombo to predict the efficacy of targeted therapies, which are often only effective in specific molecular subsets of cancer. Two of the clinical trials in our dataset tested targeted therapies and reported full study results for patients with and without the molecular features targeted by those therapies. We made power predictions for these two trials using sets of cell lines with or without the relevant molecular features for each reported patient subgroup. The resulting predictions for these trials are shown in Supplementary Data 5. Notably, IDACombo’s predictions agreed with clinical findings that there is a higher expected benefit for patients with the molecular features targeted by the targeted therapies than for patients without those molecular features. However, the subtype-specific predictions did not reach the 80% power cuttoff necessary to correctly classify the trials. This may be due to the fact that very few cell lines were available for these subtype-specific predictions, leading to relatively high prediction uncertainties and a relatively small population in which to detect phenotypic heterogeneity.

To further assess the utility of making predictions with sets of cell lines matched to patient phenotypes, we predicted clinical trial powers using cancer-specific sets of cell lines for each clinical trial (Figure S6). Note that clinical trials were excluded if fewer than 5 cancer-specific cell lines were available with which to make predictions. The cancer-specific predictions resulted in accuracies > 80% for trials in chemo-naïve patients, but model performance was generally reduced and prediction uncertainties increased for cancer-specific predictions versus pan-cancer predictions. This result, along with the analysis of the two targeted therapy trials, suggests that predictions made using cancer and subtype-specific sets of cell lines could be preferable to pan-cancer predictions if sufficient numbers of cell lines were available for each cancer type, but there are currently too few cell lines available for each cancer type in these datasets for this approach to be viable. In the meantime, pan-cancer predictions appear to be adequate for most of the drug combinations used in our clinical trial dataset.

Clinical IDACombo predictions are affected by selected drug concentrations, but remain accurate, sensitive, and specific across a range of concentrations

Beyond the selection of cell lines, we also wanted to investigate the importance of drug concentration selection for IDACombo predictions. We examined the importance of drug concentration selection by assessing whether or not prediction performance was harmed by using drug concentrations that deviated from clinical plasma concentrations. When predictions were made using the maximum concentrations tested for each drug in either CTRPv2 or GDSC rather than Csustained concentrations, prediction accuracies in treatment-naïve trials fell dramatically (65.4% accuracy for PFS/TTP and 71.4% accuracy for OS) (Figures S7A and S7B). Alternatively, when the Csustained concentrations for each drug in a trial were multiplied by factors between 0.1 and 10, we found that uniformly increasing drug concentrations kept the method’s sensitivity high but decreased accuracy, specificity, and precision for both PFS/TTP and OS. Uniformly decreasing concentrations quickly reduced sensitivity and precision (Figures S7C and S7D). These results suggest that correctly identifying clinical drug concentrations is important for *in vitro* predictions using IDA, with underestimated concentrations decreasing

model performance more than overestimated concentrations when clinical dose ratios between drugs are preserved.

Clinical IDACombo prediction accuracy drops when predicting efficacy for trials with drugs which have plasma concentrations beyond the tested in vitro concentrations

To further assess the importance of drug concentration for model performance, we looked at trials that used treatments which resulted in Csustained concentrations greater than the concentrations tested for those drugs *in vitro*. Several of the trials identified from ClinicalTrials.gov tested drugs with Csustained concentrations above the tested concentrations for those drugs in CTRPv2 or GDSC, with several trials including drugs with Csustained concentrations > 2x the tested *in vitro* concentrations in GDSC (Figures S8A and S8C). To determine whether or not this would affect IDACombo based power predictions for these trials, we calculated model performance for both PFS/TTP and OS for these trials (specifically trials in chemo-naïve patients) and compared model performance to whether or not trials included drugs with Csustained concentrations higher than tested *in vitro* concentrations. Trials with at least one drug with a Csustained concentration > 2x the maximum tested *in vitro* concentration for that drug showed largely reduced accuracy, specificity, and precision in both PFS/TTP and OS predictions relative to trials with drugs that have Csustained concentrations ≤2x the maximum tested *in vitro* concentrations (Figures S8B and S8D). As a result of this finding, only trials with drugs that have Csustained concentrations ≤2x the maximum tested *in vitro* concentrations were included in the clinical analyses in this paper.

Prospective Analysis

IDAcomboscore Clusters

As mentioned in the main text, the clusters in Figure 5 can partially be explained by drug mechanisms of action, as drugs with the same mechanism of action often end up in the same hierarchical clusters (at least, this is the case for the few mechanisms of action for which we have more than one drug). This does not fully explain the clustering, however, as we can see with topoisomerase inhibitors and EGFR inhibitors, which are divided between several small clusters. A more detailed analysis of the drugs' mechanisms of action may partially explain this, as, for the topoisomerase inhibitors, drugs are separated by whether or not they inhibit topoisomerase I or II and whether or not they act by binding DNA or intercalating DNA. This is highly speculative, however, given the small number of drugs available for each mechanism of action. It is also notable that drugs which have similar average viabilities across all cell lines when used as a monotherapy tend to be more closely clustered. This suggests that the clustering observed in Figure 5 may be explained partially by similarity in drug mechanisms and partially by similarity in the average monotherapy efficacies of drugs at their clinical concentrations. Unfortunately, a more detailed analysis of which mechanisms and monotherapy efficacies provide the most effective combinations is prevented by the limited number of drugs available for each drug mechanism.

The accuracy of cancer-specific IDACombo predictions is currently limited by the number of available cell lines for each cancer type

As discussed in the main text, we sought to determine how many cell lines are necessary to create accurate predictions using IDACombo. Since the true efficacy of most drug combinations is not known, we decided to use agreement between predictions made using

CTRPv2 and GDSC as a metric of prediction accuracy. Notably, we only compared CTRPv2 and GDSC predictions for combinations in which Csustained was available for both drugs in both datasets and which had at least 400 cell lines available to make predictions with—this resulted in comparisons for 351 drug combinations involving 27 compounds.

For the comparison, we calculated Spearman's ρ between CTRPv2 and GDSC predictions made with varying number of cell lines and plotted them in Figure S10A. This revealed that a ρ as high as 0.8 could be achieved using 250 or more cell lines, and that this correlation slowly decreased to ~0.7 as the number of cell lines was reduced to 50. With less than 50 cell lines, ρ decreased more rapidly, to ~0.6 with 25 cell lines and ~0.3 with 5 cell lines. This suggests that most cancer-specific predictions will be suboptimal, owing to their having less than 50 cell lines available to make predictions with, but it also suggests that there is some level of reproducibility using those numbers of cell lines. To quantify this reproducibility specifically for the cancer types available in CTRPv2 and GDSC, we plotted Spearman ρ's between cancer-specific IDAcomboscores versus the median number of cell lines available for each of 27 cancer types/subtypes (Figure S10B). The results largely agreed with the downsampling approach in Figure S10A, showing that Spearman ρ's for cancer-specific predictions ranged from ~0.7 to ~0.3 depending roughly on how many cell lines were available for each cancer type. A full list of correlation coefficients for each cancer type can be found in Data S6. These findings suggest that highly reproducible cancer-specific predictions are currently possible for some cancer types, but IDACombo predictions for most cancer types would likely be significantly improved by increasing the number of cell lines available for those cancer types.

SUPPLEMENTAL DISCUSSION

As briefly mentioned in the main text, there are several limitations of our method that must be considered when using it in the future.

First, while IDACombo's predicted efficacies strongly correlate with measured efficacies in NCI-ALMANAC and deviations of predicted efficacies from measured efficacies are generally small, it is still obvious that examples can be found where the measured effect of a drug combination is significantly different from the predicted effect. These may represent true cases of drug synergy, additivity, or antagonism, and the drug interactions present in these combinations could have a significant impact on the clinical behavior of these treatments. Given this result and the fact that synergistic drug combinations are likely to outperform combinations that work via IDA[9], it is likely that predictions based on IDA will fail to identify a subset of highly effective drug combinations. Synergy and additivity based prediction methods will need to be developed to identify such combinations. Fortunately, however, the results of our clinical trial validation analysis suggest that this is not a problem for most clinical drug combinations, as the large majority of them were predicted well using IDACombo, at least for trials in previously untreated patients.

This brings us to a second, and perhaps more serious, limitation of the method, which is an apparent unsuitability of cell-line based IDA predictions for patients who have undergone previous cancer drug treatment. We do not have sufficient data from our analyses to definitively explain this finding, but we can propose several hypotheses for future testing. First, there is the possibility that the difference in model performance between previously treated and previously untreated patients is coincidental—merely due to the model working better for some drugs than

for others and to different drugs being tested in trials of previously treated or untreated patients. Upon a closer inspection of the drugs involved in misclassified trials, however, we believe this is unlikely to be the case. Of the 12 drugs involved in trials that were misclassified for PFS/TTP improvement, all except vandetanib and nintedanib (which were both used in only a single trial) were also used in trials that were correctly classified, and 8 of the 12 drugs were used in correctly classified trials at least as often as they were used in misclassified trials. A more likely explanation for this finding could be that the cell line models in CTRPv2 and GDSC may more accurately represent chemo-naïve tumors than previously treated tumors. It is well known that drug treatment can induce clonal selection in tumors in ways that alter the tumors' drug sensitivities[10]. While these altered sensitivities may be reflected in cell lines that were generated from the tumors of previously treated patients[11], it is likely that the cell lines in CTRPv2 and GDSC were derived under a diverse set of circumstances. As such, we would not expect our population of available cell lines to be a good representation of a population of tumors which had all recently received similar drug treatments. In the future, it may be possible to test this hypothesis by creating panels of cell lines that are derived from patients who had received the same prior therapies as the patients in the trials which were poorly predicted in this study and then test whether predictions made with these cell line panels agree with the clinical findings of those trials.

A third limitation of this study is that our method is currently unable to make predictions for combinations which include immunotherapies or drugs which function by acting systemically on non-tumor cells, such as drugs that act systemically to block hormone synthesis. This is because our predictions rely on *in vitro* drug screening data, and the *in vitro* systems that have been used for high-throughput cancer cell line drug screens lack the ability to mimic immune responses or non-tumor processes such as systemic hormone production. This does not mean, however, that IDA based predictions of drug combination efficacy are unsuitable for immunotherapies or drugs which act outside of the tumor. Efforts are underway to generate *in vitro* models which may be suitable for screening immunotherapies in the future[12] and which could allow for IDA based predictions to be made for immunotherapy combinations. While those models mature, however, IDA based predictions of efficacy for combinations with immunotherapies/systemically acting therapies may be made using the results of monotherapy based clinical trials and the method developed by Palmer and Sorger[9], providing that cross-resistance can be estimated between combined treatments.

Despite these limitations, our results are notable for several reasons which are briefly discussed in the main text. A more detailed discussion of these reasons is as follows.

First, these results demonstrate that *in vitro* drug screening data can be used to generate clinically meaningful predictions for drug combination efficacies in patients, and, furthermore, they suggest that many of these predictions can be made using pan-cancer sets of cell lines. This is somewhat unexpected given the wide range of genetic and phenotypic diversities observed between different cancer types. On the other hand, our results suggest that it will be necessary to make predictions using cell lines of the appropriate cancer type/subtype for targeted therapies, and we believe it is likely that cancer-specific IDACombo predictions could be comparable to or better than pan-cancer predictions if not for the fact that many cancer types currently have relatively few available cell lines in CTRPv2 and GDSC. The solution to this problem, however, may be more complicated than simply increasing the number of cell lines for each cancer type. That is because it must also be noted that, beyond the limited numbers of cell

lines available for many cancer types, the ethnic diversity of available cancer cell lines is also very limited—particularly for ethnicities other than Caucasian or Asian [13]. This means that caution will be necessary when applying the predictions made in this paper to ethnicities that are poorly represented in the cell lines currently available in CTRPv2 and GDSC. Fortunately, others in the field have already recognized the need to increase the number and genetic diversity of available cancer cell lines [14], and the Broad Institute has received an NCI contract to create new cancer cell lines (https://portals.broadinstitute.org/cellfactory). This has already lead to the creation of over 100 validated cancer models. The use of these models in future monotherapy drug screens may improve predictions made with IDACombo even further.

A second reason that the success of IDACombo is notable is that, despite our extensive efforts to identify clinical relevant drug concentrations for each drug in our analysis, these concentrations remain only rough estimates of true clinically relevant concentrations. Beyond the fact that measured plasma concentrations are simply unavailable for some drugs and doses for patients of each cancer type, there is little available information about how plasma drug concentrations relate to intratumoral drug concentrations *in vivo.* Similarly, there is little available information about how media drug concentrations relate to intracellular drug concentrations *in vitro*. In the single study we were able to find that did examine these relationships, researchers found that the clinically relevant *in vitro* drug concentration for paclitaxel may be an order of magnitude below clinically measured plasma concentrations [15]. Even with this information, the appropriate paclitaxel concentration to use for different cancer types is unclear, because the concentrations identified in the study were based on only two cell lines and six patients in a single cancer type. Given that our results suggest that varying drug concentrations can significantly affect prediction performance, it is possible that IDACombo predictions could be improved by future research aimed at identifying the *in vitro* drug concentrations that most closely mimic the drug exposure of tumor cells in the clinic. It is notable, however, that IDACombo works as well as it does—especially given the high uncertainties in the drug concentrations we used to estimate clinical trial powers. It is our hope that this method will help researchers identify promising combinations for future clinical development and that they will ultimately lead to improved therapies for cancer patients.

SUPPLEMENTAL REFERENCES

(Note that this section includes references from the supplemental tables)

1. Gridelli, C. *et al.* Chemotherapy for Elderly Patients With Advanced Non-Small-Cell Lung Cancer: The Multicenter Italian Lung Cancer in the Elderly Study (MILES) Phase III Randomized Trial. *JNCI J. Natl. Cancer Inst.* **95**, 362–372 (2003).

2. National Comprehensive Cancer Network. NCCN Clinical Practice Guidelines in Oncology – Non-Small Cell Lung Cancer. V3.2020. (2020).

3. The ICON Group. Paclitaxel plus carboplatin versus standard chemotherapy with either single-agent carboplatin or cyclophosphamide, doxorubicin, and cisplatin in women with ovarian cancer: the ICON3 randomised trial. *Lancet Lond. Engl.* **360**, 505–515 (2002).

4. Bois, A. du *et al.* Phase III Trial of Carboplatin Plus Paclitaxel With or Without Gemcitabine in First-Line Treatment of Epithelial Ovarian Cancer. *J. Clin. Oncol.* (2010) doi:10.1200/JCO.2009.27.4696.

5. National Comprehensive Cancer Network. NCCN Clinical Practice Guidelines in Oncology – Ovarian Cancer Including Fallopian Tube Cancer and Primary Peritoneal Cancer. V3.2019. (2019).

6. Boyd, L. R. & Muggia, F. M. Carboplatin/Paclitaxel Induction in Ovarian Cancer: The Finer Points. *Oncol. Williston Park N* **32**, 418–420, 422–424 (2018).

7. Hall, M. D. *et al.* Say No to DMSO: Dimethylsulfoxide Inactivates Cisplatin, Carboplatin and Other Platinum Complexes. *Cancer Res.* **74**, 3913–3922 (2014).

8. Ray-Coquard, I. *et al.* Final results from GCIG/ENGOT/AGO-OVAR 12, a randomised placebo-controlled phase III trial of nintedanib combined with chemotherapy for newly diagnosed advanced ovarian cancer. *Int. J. Cancer* **146**, 439–448 (2020).

9. Palmer, A. C. & Sorger, P. K. Combination Cancer Therapy Can Confer Benefit via Patient-to-Patient Variability without Drug Additivity or Synergy. *Cell* **171**, 1678-1691.e13 (2017).

10. Ibragimova, M. K., Tsyganov, M. M. & Litviakov, N. V. Natural and chemotherapy-induced clonal evolution of tumors. *Biochem. Mosc.* **82**, 413–425 (2017).

11. Berendsen, H. H. *et al.* Characterization of Three Small Cell Lung Cancer Cell Lines Established from One Patient during Longitudinal Follow-up. *Cancer Res.* **48**, 6891–6899 (1988).

12. Dijkstra, K. K. *et al.* Generation of Tumor-Reactive T Cells by Co-culture of Peripheral Blood Lymphocytes and Tumor Organoids. *Cell* **174**, 1586-1598.e12 (2018).

13. Ling, A., Gruener, R. F., Fessler, J. & Huang, R. S. More than fishing for a cure: The promises and pitfalls of high throughput cancer cell line screens. *Pharmacol. Ther.* **191**, 178–189 (2018).

14. Boehm, J. S. & Golub, T. R. An ecosystem of cancer cell line factories to support a cancer dependency map. *Nat. Rev. Genet.* **16**, 373–374 (2015).

15. Zasadil, L. M. *et al.* Cytotoxicity of paclitaxel in breast cancer is due to chromosome missegregation on multipolar spindles. *Sci. Transl. Med.* **6**, 229ra43 (2014).

16. Aoki, D. *et al.* A phase II clinical trial of topotecan in Japanese patients with relapsed ovarian carcinoma. *Jpn. J. Clin. Oncol.* **41**, 320–327 (2011).

17. Baker, S. D. *et al.* Comparative Pharmacokinetics of Weekly and Every-Three-Weeks Docetaxel. *Clin. Cancer Res.* **10**, 1976–1983 (2004).

18. Bocci, G. *et al.* Comparative pharmacokinetic analysis of 5-fluorouracil and its major metabolite 5-fluoro-5,6-dihydrouracil after conventional and reduced test dose in cancer patients. *Clin. Cancer Res. Off. J. Am. Assoc. Cancer Res.* **6**, 3032–3037 (2000).

19. Bonneterre, J., Chevalier, B., Focan, C., Mauriac, L. & Piccart, M. Phase I and pharmacokinetic study of weekly oral therapy with vinorelbine in patients with advanced breast cancer (ABC). *Ann. Oncol. Off. J. Eur. Soc. Med. Oncol.* **12**, 1683–1691 (2001).

20. Brooks, D. J. *et al.* Phase I and pharmacokinetic study of etoposide phosphate. *Anticancer. Drugs* **6**, 637–644 (1995).

21. Burz, C. *et al.* Clinical and pharmacokinetics study of oxaliplatin in colon cancer patients. *J. Gastrointest. Liver Dis. JGLD* **18**, 39–43 (2009).

22. Chabot, G. G. *et al.* Population pharmacokinetics and pharmacodynamics of irinotecan (CPT-11) and active metabolite SN-38 during phase I trials. *Ann. Oncol.* **6**, 141–151 (1995).

23. Chen, T.-L. *et al.* Nonlinear Pharmacokinetics of Cyclophosphamide in Patients with Metastatic Breast Cancer Receiving High-Dose Chemotherapy followed by Autologous Bone Marrow Transplantation. *Cancer Res.* **55**, 810–816 (1995).

24. Comandone, A. *et al.* High dose methotrexate in adult patients with osteosarcoma: clinical and pharmacokinetic results. *Acta Oncol. Stockh. Swed.* **44**, 406–411 (2005).

25. Danhauser, L. L. *et al.* Phase I and plasma pharmacokinetic study of infusional fluorouracil combined with recombinant interferon alfa-2b in patients with advanced cancer. *J. Clin. Oncol.* **11**, 751–761 (1993).

26. Diamant, Z., Samuelsson Palmgren, G., Westrin, B. & Bjermer, L. Phase I study evaluating the safety, tolerability and pharmacokinetics of a novel oral dissolvable film containing dexamethasone versus Fortecortin dexamethasone tablets. *Eur. Clin. Respir. J.* **4**, (2017).

27. Doebele, R. C. *et al.* A phase I, open-label dose-escalation study of continuous treatment with BIBF 1120 in combination with paclitaxel and carboplatin as first-line treatment in patients with advanced non-small-cell lung cancer. *Ann. Oncol.* **23**, 2094–2102 (2012).

28. Doi, T. *et al.* Phase 1 pharmacokinetic study of the oral pan-AKT inhibitor MK-2206 in Japanese patients with advanced solid tumors. *Cancer Chemother. Pharmacol.* **76**, 409–416 (2015).

29. Fakih, M. G. *et al.* A phase I, pharmacokinetic, and pharmacodynamic study of two schedules of vorinostat in combination with 5-fluorouracil and leucovorin in patients with refractory solid tumors. *Clin. Cancer Res. Off. J. Am. Assoc. Cancer Res.* **16**, 3786–3794 (2010).

30. Fraile, R. J., Baker, L. H., Buroker, T. R., Horwitz, J. & Vaitkevicius, V. K. Pharmacokinetics of 5-Fluorouracil Administered Orally, by Rapid Intravenous and by Slow Infusion. *Cancer Res.* **40**, 2223–2228 (1980).

31. Fujisaka, Y. *et al.* Phase 1 clinical study of pegylated liposomal doxorubicin (JNS002) in Japanese patients with solid tumors. *Jpn. J. Clin. Oncol.* **36**, 768–774 (2006).

32. Fumoleau, P. *et al.* A phase I pharmacokinetics study of lapatinib and tamoxifen in metastatic breast cancer (EORTC 10053 Lapatam study). *The Breast* **23**, 663–669 (2014).

33. Grahnén, A., von Bahr, C., Lindström, B. & Rosén, A. Bioavailability and pharmacokinetics of cimetidine. *Eur. J. Clin. Pharmacol.* **16**, 335–340 (1979).

34. Greene, R. F., Collins, J. M., Jenkins, J. F., Speyer, J. L. & Myers, C. E. Plasma Pharmacokinetics of Adriamycin and Adriamycinol: Implications for the Design of in Vitro Experiments and Treatment Protocols. *Cancer Res.* **43**, 3417–3421 (1983).

35. Hageboutros, A. *et al.* Phase I study of phosphonacetyl-L-aspartate, 5-fluorouracil, and leucovorin in patients with advanced cancer. *Cancer Chemother. Pharmacol.* **35**, 205–212 (1995).

36. Hartigh, J. den, McVie, J. G., Oort, W. J. V. & Pinedo, H. M. Pharmacokinetics of Mitomycin C in Humans. *Cancer Res.* **43**, 5017–5021 (1983).

37. Hidalgo, M. *et al.* Phase I and Pharmacologic Study of OSI-774, an Epidermal Growth Factor Receptor Tyrosine Kinase Inhibitor, in Patients With Advanced Solid Malignancies. *J. Clin. Oncol.* **19**, 3267–3279 (2001).

38. Huang, S.-Y. *et al.* Pharmacokinetic study of bortezomib administered intravenously in Taiwanese patients with multiple myeloma. *Hematol. Oncol.* **36**, 238–244 (2018).

39. Ibrahim, N. K. *et al.* Phase I and Pharmacokinetic Study of ABI-007, a Cremophor-free, Protein-stabilized, Nanoparticle Formulation of Paclitaxel. *Clin. Cancer Res.* **8**, 1038–1044 (2002).

40. Ikeda, K. *et al.* Pharmacokinetics of Cisplatin in Combined Cisplatin and 5-Fluorouracil Therapy: A Comparative Study of Three Different Schedules of Cisplatin Administration. *Jpn. J. Clin. Oncol.* **28**, 168–175 (1998).

41. Inoue, K. *et al.* Safety, pharmacokinetics and efficacy findings in an open-label, single-arm study of weekly paclitaxel plus lapatinib as first-line therapy for Japanese women with HER2-positive metastatic breast cancer. *Int. J. Clin. Oncol.* **20**, 1102–1109 (2015).

42. Jain, L. *et al.* Population pharmacokinetic analysis of sorafenib in patients with solid tumours. *Br. J. Clin. Pharmacol.* **72**, 294–305 (2011).

43. Kerbusch, T. *et al.* Influence of Dose and Infusion Duration on Pharmacokinetics of Ifosfamide and Metabolites. *Drug Metab. Dispos.* **29**, 967–975 (2001).

44. Keyvanjah, K. *et al.* Pharmacokinetics of neratinib during coadministration with lansoprazole in healthy subjects. *Br. J. Clin. Pharmacol.* **83**, 554–561 (2017).

45. Kurata, T. *et al.* Phase I and Pharmacological Study of Paclitaxel Given Over 3 h with Cisplatin for Advanced Non-small Cell Lung Cancer. *Jpn. J. Clin. Oncol.* **31**, 93–99 (2001).

46. Lankheet, N. A. G. *et al.* Pharmacokinetically guided sunitinib dosing: a feasibility study in patients with advanced solid tumours. *Br. J. Cancer* **110**, 2441–2449 (2014).

47. Levêque, D., Jehl, F., Quoix, E. & Breillout, F. Clinical Pharmacokinetics of Vinorelbine Alone and Combined with Cisplatin. *J. Clin. Pharmacol.* **32**, 1096–1098 (1992).

48. Liston, D. R. & Davis, M. Clinically Relevant Concentrations of Anticancer Drugs: A Guide for Nonclinical Studies. *Clin. Cancer Res.* **23**, 3489–3498 (2017).

49. Ma, W. W. *et al.* Phase I Study of Rigosertib, an Inhibitor of the Phosphatidylinositol 3-Kinase and Polo-like Kinase 1 Pathways, Combined with Gemcitabine in Patients with Solid Tumors and Pancreatic Cancer. *Clin. Cancer Res.* **18**, 2048–2055 (2012).

50. Mavroudis, D. *et al.* A dose-escalation and pharmacokinetic study of gemcitabine and oxaliplatin in patients with advanced solid tumors. *Ann. Oncol. Off. J. Eur. Soc. Med. Oncol.* **14**, 304–312 (2003).

51. Mehrotra, S. *et al.* Population pharmacokinetics and site of action exposures of veliparib with topotecan plus carboplatin in patients with haematological malignancies. *Br. J. Clin. Pharmacol.* **83**, 1688–1700 (2017).

52. Moloney, M. *et al.* Feasibility of 5-fluorouracil pharmacokinetic monitoring using the My-5FU PCM^{TM} system in a quaternary oncology centre. *Cancer Chemother. Pharmacol.* **82**, 865–876 (2018).

53. Müller, M. *et al.* Interstitial methotrexate kinetics in primary breast cancer lesions. *Cancer Res.* **58**, 2982–2985 (1998).

54. Nokihara, H., Yamamoto, N., Ohe, Y., Hiraoka, M. & Tamura, T. Pharmacokinetics of Weekly Paclitaxel and Feasibility of Dexamethasone Taper in Japanese Patients with Advanced Non–small Cell Lung Cancer. *Clin. Ther.* **38**, 338–347 (2016).

55. Rajkumar, P. *et al.* Cisplatin Concentrations in Long and Short Duration Infusion: Implications for the Optimal Time of Radiation Delivery. *J. Clin. Diagn. Res.* **10**, XC01–XC04 (2016).

56. Reigner, B., Blesch, K. & Weidekamm, E. Clinical Pharmacokinetics of Capecitabine. *Clin. Pharmacokinet.* **40**, 85–104 (2001).

57. Rugo, H. S. *et al.* Phase I Trial of the Oral Antiangiogenesis Agent AG-013736 in Patients With Advanced Solid Tumors: Pharmacokinetic and Clinical Results. *J. Clin. Oncol.* **23**, 5474–5483 (2005).

58. Shiah, H.-S. *et al.* Phase I and pharmacokinetic study of oral thalidomide in patients with advanced hepatocellular carcinoma. *Cancer Chemother. Pharmacol.* **58**, 654–664 (2006).

59. Shirao, K. *et al.* Phase I Study of Single-Dose Oxaliplatin in Japanese Patients with Malignant Tumors. *Jpn. J. Clin. Oncol.* **36**, 295–300 (2006).

60. Speth, P. A. J., Linssen, P. C. M., Holdrinet, R. S. G. & Haanen, C. Plasma and cellular Adriamycin concentrations in patients with myeloma treated with ninety-six-hour continuous infusion. *Clin. Pharmacol. Ther.* **41**, 661–665 (1987).

61. Speyer, J. L. *et al.* Prospective evaluation of cardiotoxicity during a six-hour doxorubicin infusion regimen in women with adenocarcinoma of the breast. *Am. J. Med.* **78**, 555–563 (1985).

62. Tchekmedyian, N. S. *et al.* Phase I clinical and pharmacokinetic study of cyclophosphamide administered by five-day continuous intravenous infusion. *Cancer Chemother. Pharmacol.* **18**, 33–38 (1986).

63. Terret, C. *et al.* Dose and time dependencies of 5-fluorouracil pharmacokinetics. *Clin. Pharmacol. Ther.* **68**, 270–279 (2000).

64. Van Veggel, M., Westerman, E. & Hamberg, P. Clinical Pharmacokinetics and Pharmacodynamics of Panobinostat. *Clin. Pharmacokinet.* **57**, 21–29 (2018).

65. Wada, T. *et al.* Pharmacokinetic analyses of carboplatin in a patient with cancer of the fallopian tubes undergoing hemodialysis: A case report. *Biomed. Rep.* **5**, 199–202 (2016).

66. Wang, X. *et al.* Differential effects of dosing regimen on the safety and efficacy of dasatinib: retrospective exposure–response analysis of a Phase III study. *Clin. Pharmacol. Adv. Appl.* **5**, 85–97 (2013).

67. Xue, C. *et al.* Randomized, Multicenter Study of Gefitinib Dose-escalation in Advanced Non-small-cell Lung Cancer Patients Achieved Stable Disease after One-month Gefitinib Treatment. *Sci. Rep.* **5**, 10648 (2015).

68. Yamamoto, N. *et al.* A Phase I, dose-finding and pharmacokinetic study of olaparib (AZD2281) in Japanese patients with advanced solid tumors. *Cancer Sci.* **103**, 504–509 (2012).

69. Yamamoto, N. *et al.* CYP2C19 genotype-based phase I studies of a c-Met inhibitor tivantinib in combination with erlotinib, in advanced/metastatic non-small cell lung cancer. *Br. J. Cancer* **109**, 2803–2809 (2013).

70. Yamazaki, N. *et al.* Phase 1/2 study assessing the safety and efficacy of dabrafenib and trametinib combination therapy in Japanese patients with BRAF V600 mutation-positive advanced cutaneous melanoma. *J. Dermatol.* **45**, 397–407 (2018).

71. Yen, C.-J. *et al.* A Phase I/Randomized Phase II Study to Evaluate the Safety, Pharmacokinetics, and Efficacy of Nintedanib versus Sorafenib in Asian Patients with Advanced Hepatocellular Carcinoma. *Liver Cancer* **7**, 165–178 (2018).

72. Zhang, L. *et al.* Pharmacokinetics and Tolerability of Vandetanib in Chinese Patients With Solid, Malignant Tumors: An Open-Label, Phase I, Rising Multiple-Dose Study. *Clin. Ther.* **33**, 315–327 (2011).

73. Abbas, R. *et al.* A phase I ascending single-dose study of the safety, tolerability, and pharmacokinetics of bosutinib (SKI-606) in healthy adult subjects. *Cancer Chemother. Pharmacol.* **69**, 221–227 (2012).

74. Amaravadi, R. K. *et al.* A Phase 1 Study of the SMAC-Mimetic Birinapant in Adults with Refractory Solid Tumors or Lymphoma. *Mol. Cancer Ther.* **14**, 2569–2575 (2015).

75. Attard, G. *et al.* Phase I Clinical Trial of a Selective Inhibitor of CYP17, Abiraterone Acetate, Confirms That Castration-Resistant Prostate Cancer Commonly Remains Hormone Driven. *J. Clin. Oncol.* **26**, 4563–4571 (2008).

76. Balis, F. M. *et al.* First-dose and steady-state pharmacokinetics of orally administered crizotinib in children with solid tumors: a report on ADVL0912 from the Children's Oncology Group Phase 1/Pilot Consortium. *Cancer Chemother. Pharmacol.* **79**, 181–187 (2017).

77. Berkenblit, A. *et al.* Phase I Clinical Trial of STA-4783 in Combination with Paclitaxel in Patients with Refractory Solid Tumors. *Clin. Cancer Res.* **13**, 584–590 (2007).

78. Breithaupt, H., Dammann, A. & Aigner, K. Pharmacokinetics of dacarbazine (DTIC) and its metabolite 5-aminoimidazole-4-carboxamide (AIC) following different dose schedules. *Cancer Chemother. Pharmacol.* **9**, 103–109 (1982).

79. Cantarovich, F. *et al.* Cyclosporine plasma levels six hours after oral administration. A useful tool for monitoring therapy. *Transplantation* **45**, 389–394 (1988).

80. Cheng, A. L. *et al.* Phase I clinical trial of curcumin, a chemopreventive agent, in patients with high-risk or pre-malignant lesions. *Anticancer Res.* **21**, 2895–2900 (2001).

81. Dahut, W. L. *et al.* A Phase I Study of Oral Lenalidomide in Patients with Refractory Metastatic Cancer. *J. Clin. Pharmacol.* **49**, 650–660 (2009).

82. Davids, M. S. *et al.* Phase I First-in-Human Study of Venetoclax in Patients With Relapsed or Refractory Non-Hodgkin Lymphoma. *J. Clin. Oncol.* **35**, 826–833 (2017).

83. European Medicines Agency. Assessment report: Alsitek. https://www.ema.europa.eu/en/documents/assessment-report/alsitek-epar-refusal-public-assessment-report_en.pdf (2018).

84. Gadgeel, S. M. *et al.* Safety and activity of alectinib against systemic disease and brain metastases in patients with crizotinib-resistant ALK-rearranged non-small-cell lung cancer (AF-002JG): results from the dose-finding portion of a phase 1/2 study. *Lancet Oncol.* **15**, 1119–1128 (2014).

85. Ghosal, K. *et al.* A randomized controlled study to evaluate the effect of bexarotene on amyloid-β and apolipoprotein E metabolism in healthy subjects. *Alzheimers Dement. Transl. Res. Clin. Interv.* **2**, 110–120 (2016).

86. Gojo, I. *et al.* Phase 1 and pharmacologic study of MS-275, a histone deacetylase inhibitor, in adults with refractory and relapsed acute leukemias. *Blood* **109**, 2781–2790 (2007).

87. Gueorguieva, I. *et al.* Relative bioavailability of three formulations of galunisertib administered as monotherapy in patients with advanced or metastatic cancer. *Drugs Context* **5**, 212303 (2016).

88. Hexner, E. *et al.* Open-label study of oral CEP-701 (lestaurtinib) in patients with polycythaemia vera or essential thrombocythaemia with JAK2-V617F mutation. *Br. J. Haematol.* **164**, 83–93 (2014).

89. Holen, K., Saltz, L. B., Hollywood, E., Burk, K. & Hanauske, A.-R. The pharmacokinetics, toxicities, and biologic effects of FK866, a nicotinamide adenine dinucleotide biosynthesis inhibitor. *Invest. New Drugs* **26**, 45–51 (2008).

90. Howell, S. B., Schiefer, M., Andrews, P. A., Markman, M. & Abramson, I. The pharmacology of intraperitoneally administered bleomycin. *J. Clin. Oncol.* **5**, 2009–2016 (1987).

91. Huang, L., Lizak, P., Dvorak, C. C., Aweeka, F. & Long-Boyle, J. Simultaneous determination of fludarabine and clofarabine in human plasma by LC-MS/MS. *J. Chromatogr. B Analyt. Technol. Biomed. Life. Sci.* **960**, 194–199 (2014).

92. Ikeda, M. *et al.* Safety and Pharmacokinetics of Lenvatinib in Patients with Advanced Hepatocellular Carcinoma. *Clin. Cancer Res.* **22**, 1385–1394 (2016).

93. Jin, F., Robeson, M., Zhou, H., Hisoire, G. & Ramanathan, S. The pharmacokinetics and safety of idelalisib in subjects with moderate or severe hepatic impairment. *J. Clin. Pharmacol.* **55**, 944–952 (2015).

94. de Jong, J. *et al.* The effect of food on the pharmacokinetics of oral ibrutinib in healthy participants and patients with chronic lymphocytic leukemia. *Cancer Chemother. Pharmacol.* **75**, 907–916 (2015).

95. Kantarjian, H. M. *et al.* Phase I study assessing the safety and tolerability of barasertib (AZD1152) with low-dose cytosine arabinoside in elderly patients with AML. *Clin. Lymphoma Myeloma Leuk.* **13**, 559–567 (2013).

96. Kovarik, J. M. *et al.* Oral-intravenous crossover study of fingolimod pharmacokinetics, lymphocyte responses and cardiac effects. *Biopharm. Drug Dispos.* **28**, 97–104 (2007).

97. Lacy, S. A., Miles, D. R. & Nguyen, L. T. Clinical Pharmacokinetics and Pharmacodynamics of Cabozantinib. *Clin. Pharmacokinet.* **56**, 477–491 (2017).

98. Lee, S. *et al.* Relative Bioavailability and Tolerability of Two Formulations of Bicalutamide 50-mg Tablets: A Randomized-Sequence, Open-Label, Two-Period Crossover Study in Healthy Korean Male Subjects. *Clin. Ther.* **32**, 2496–2501 (2010).

99. Macaulay, V. M. *et al.* Phase I Dose-Escalation Study of Linsitinib (OSI-906) and Erlotinib in Patients with Advanced Solid Tumors. *Clin. Cancer Res. Off. J. Am. Assoc. Cancer Res.* **22**, 2897–2907 (2016).

100. Marumo, A., Miyawaki, S., Dan, N. & Ishiyama, K. Plasma Concentration of Itraconazole in Patients With Hematologic Malignancies Treated With Itraconazole Oral Solution. *Ther. Drug Monit.* **39**, 229–234 (2017).

101. Mekhail, T. *et al.* Metabolism, Excretion, and Pharmacokinetics of Oral Brivanib in Patients with Advanced or Metastatic Solid Tumors. *Drug Metab. Dispos.* **38**, 1962–1966 (2010).

102. Minami, H. *et al.* Phase I, multicenter, open-label, dose-escalation study of sonidegib in Asian patients with advanced solid tumors. *Cancer Sci.* **107**, 1477–1483 (2016).

103. Minden, M. D. *et al.* Oral ciclopirox olamine displays biological activity in a phase I study in patients with advanced hematologic malignancies. *Am. J. Hematol.* **89**, 363–368 (2014).

104. Mross, K. *et al.* A phase I clinical and pharmacokinetic study of the camptothecin glycoconjugate, BAY 38-3441, as a daily infusion in patients with advanced solid tumors. *Ann. Oncol.* **15**, 1284–1294 (2004).

105. Mukai, M. *et al.* Effects of Rifampin on the Pharmacokinetics of a Single Dose of Istradefylline in Healthy Subjects. *J. Clin. Pharmacol.* **58**, 193–201 (2018).

106. Mukohara, T., Nagai, S., Koshiji, M., Yoshizawa, K. & Minami, H. Phase I dose escalation and pharmacokinetic study of oral enzastaurin (LY317615) in advanced solid tumors. *Cancer Sci.* **101**, 2193–2199 (2010).

107. Nemunaitis, J. J. *et al.* A first-in-human, phase 1, dose-escalation study of dinaciclib, a novel cyclin-dependent kinase inhibitor, administered weekly in subjects with advanced malignancies. *J. Transl. Med.* **11**, 259 (2013).

108. Ogura, M. *et al.* Phase I and pharmacokinetic study of bendamustine hydrochloride in relapsed or refractory indolent B-cell non-Hodgkin lymphoma and mantle cell lymphoma. *Cancer Sci.* **101**, 2054–2058 (2010).

109. Oki, Y. *et al.* Phase I/II study of decitabine in patients with myelodysplastic syndrome: A multi-center study in Japan. *Cancer Sci.* **103**, 1839–1847 (2012).

110. Park, Y. B., Kim, H. S., Oh, J. H. & Lee, S. H. The co-expression of p53 protein and P-glycoprotein is correlated to a poor prognosis in osteosarcoma. *Int. Orthop.* **24**, 307–310 (2001).

111. Peng, B. *et al.* Pharmacokinetics and Pharmacodynamics of Imatinib in a Phase I Trial With Chronic Myeloid Leukemia Patients. *J. Clin. Oncol.* **22**, 935–942 (2004).

112. Robertson, J. F. R. *et al.* Pharmacokinetic Profile of Intramuscular Fulvestrant in Advanced Breast Cancer. *Clin. Pharmacokinet.* **43**, 529–538 (2004).

113. Rodon, J. *et al.* A Phase Ib, open-label, dose-finding study of alpelisib in combination with paclitaxel in patients with advanced solid tumors. *Oncotarget* **9**, 31709–31718 (2018).

114. Saka, H. *et al.* Safety, tolerability and pharmacokinetics of the fibroblast growth factor receptor inhibitor AZD4547 in Japanese patients with advanced solid tumours: a Phase I study. *Invest. New Drugs* **35**, 451–462 (2017).

115. Salem, A. H., Koenig, D. & Carlson, D. Pooled Population Pharmacokinetic Analysis of Phase I, II and III Studies of Linifanib in Cancer Patients. *Clin. Pharmacokinet.* **53**, 347–359 (2014).

116. Silvennoinen, R., Malminiemi, K., Malminiemi, O., Seppälä, E. & Vilpo, J. Pharmacokinetics of chlorambucil in patients with chronic lymphocytic leukaemia: comparison of different days, cycles and doses. *Pharmacol. Toxicol.* **87**, 223–228 (2000).

117. Simon, G. R. *et al.* Increased Bioavailability of Intravenous Versus Oral CI-1033, a Pan erbB Tyrosine Kinase Inhibitor: Results of a Phase I Pharmacokinetic Study. *Clin. Cancer Res.* **12**, 4645–4651 (2006).

118. Steele, N. L. *et al.* Pharmacokinetic and pharmacodynamic properties of an oral formulation of the histone deacetylase inhibitor Belinostat (PXD101). *Cancer Chemother. Pharmacol.* **67**, 1273–1279 (2011).

119. Sun, J. X. *et al.* Comparative pharmacokinetics of lovastatin extended-release tablets and lovastatin immediate-release tablets in humans. *J. Clin. Pharmacol.* **42**, 198–204 (2002).

120. Tsimberidou, A. M. *et al.* Phase I study of azacitidine and oxaliplatin in patients with advanced cancers that have relapsed or are refractory to any platinum therapy. *Clin. Epigenetics* **7**, 29 (2015).

121. Tsutsumi, H. *et al.* [Plasma concentration of cytosine arabinoside (Ara-C) in the elderly patients with hematological malignancy treated by Ara-C or cytarabine ocfosfate (SPAC)]. *Nihon Ronen Igakkai Zasshi Jpn. J. Geriatr.* **32**, 190–194 (1995).

122. Wind, S., Schmid, M., Erhardt, J., Goeldner, R.-G. & Stopfer, P. Pharmacokinetics of Afatinib, a Selective Irreversible ErbB Family Blocker, in Patients with Advanced Solid Tumours. *Clin. Pharmacokinet.* **52**, 1101–1109 (2013).

123. Yamamoto, N. *et al.* Phase I, dose escalation and pharmacokinetic study of cediranib (RECENTIN^{TM}), a highly potent and selective VEGFR signaling inhibitor, in Japanese patients with advanced solid tumors. *Cancer Chemother. Pharmacol.* **64**, 1165–1172 (2009).

124. Zhang, M. *et al.* A randomized, placebo-controlled study of the pharmacokinetics, pharmacodynamics, and tolerability of the oral JAK2 inhibitor fedratinib (SAR302503) in healthy volunteers. *J. Clin. Pharmacol.* **54**, 415–421 (2014).

125. Aoki, T. *et al.* Pharmacokinetic study of temozolomide on a daily-for-5-days schedule in Japanese patients with relapsed malignant gliomas: first study in Asians. *Int. J. Clin. Oncol.* **12**, 341–349 (2007).

126. Bergman, A. *et al.* Absolute bioavailability of sitagliptin, an oral dipeptidyl peptidase-4 inhibitor, in healthy volunteers. *Biopharm. Drug Dispos.* **28**, 315–322 (2007).

127. Blumenschein, G. R. *et al.* Phase 1b Study of Motesanib, an Oral Angiogenesis Inhibitor, in Combination with Carboplatin/Paclitaxel and/or Panitumumab for the Treatment of Advanced Non–Small Cell Lung Cancer. *Clin. Cancer Res.* **16**, 279–290 (2010).

128. de Bono, J. *et al.* Phase I, Dose-Escalation, Two-Part Trial of the PARP Inhibitor Talazoparib in Patients with Advanced Germline BRCA1/2 Mutations and Selected Sporadic Cancers. *Cancer Discov.* **7**, 620–629 (2017).

129. Canal, P. *et al.* Pharmacokinetics of teniposide (VM 26) after IV administration in serum and malignant ascites of patients with ovarian carcinoma. *Cancer Chemother. Pharmacol.* **15**, 149–152 (1985).

130. Choo, S. P. *et al.* A Phase 1 dose-finding and pharmacodynamic study of rapamycin in combination with bevacizumab in patients with unresectable hepatocellular carcinoma. *Eur. J. Cancer* **49**, 999–1008 (2013).

131. Chu, N.-N., Chen, W.-L., Xu, H.-R. & Li, X.-N. Pharmacokinetics and Safety of Ezetimibe/Simvastatin Combination Tablet. *Clin. Drug Investig.* **32**, 791–798 (2012).

132. Conley, B. A. *et al.* Phase I clinical trial of all-trans-retinoic acid with correlation of its pharmacokinetics and pharmacodynamics. *Cancer Chemother. Pharmacol.* **39**, 291–299 (1997).

133. Do, K. *et al.* Phase I Study of Single-Agent AZD1775 (MK-1775), a Wee1 Kinase Inhibitor, in Patients With Refractory Solid Tumors. *J. Clin. Oncol.* **33**, 3409–3415 (2015).

134. Dolan, M. E. *et al.* O6-benzylguanine in humans: metabolic, pharmacokinetic, and pharmacodynamic findings. *J. Clin. Oncol.* **16**, 1803–1810 (1998).

135. Fishman, M. N. *et al.* Phase Ib study of tivozanib (AV-951) in combination with temsirolimus in patients with renal cell carcinoma. *Eur. J. Cancer Oxf. Engl. 1990* **49**, 2841–2850 (2013).

136. Fujisaka, Y. *et al.* First report of the safety, tolerability, and pharmacokinetics of the Src kinase inhibitor saracatinib (AZD0530) in Japanese patients with advanced solid tumours. *Invest. New Drugs* **31**, 108–114 (2013).

137. Gandhi, V. *et al.* Compound GW506U78 in refractory hematologic malignancies: relationship between cellular pharmacokinetics and clinical response. *J. Clin. Oncol.* **16**, 3607–3615 (1998).

138. Graham, R. A. *et al.* Pharmacokinetics of Hedgehog Pathway Inhibitor Vismodegib (GDC-0449) in Patients with Locally Advanced or Metastatic Solid Tumors: the Role of Alpha-1-Acid Glycoprotein Binding. *Clin. Cancer Res. Off. J. Am. Assoc. Cancer Res.* **17**, 2512–2520 (2011).

139. Grippo, J. F. *et al.* A phase I, randomized, open-label study of the multiple-dose pharmacokinetics of vemurafenib in patients with BRAFV600Emutation-positive metastatic melanoma. *Cancer Chemother. Pharmacol.* **73**, 103–111 (2014).

140. Grossman, S. A. *et al.* The Effect of Enzyme-Inducing Antiseizure Drugs on the Pharmacokinetics and Tolerability of Procarbazine Hydrochloride. *Clin. Cancer Res.* **12**, 5174–5181 (2006).

141. Gupta, N. *et al.* Pharmacokinetics of ixazomib, an oral proteasome inhibitor, in solid tumour patients with moderate or severe hepatic impairment. *Br. J. Clin. Pharmacol.* **82**, 728–738 (2016).

142. Hamberg, P. *et al.* Pazopanib exposure decreases as a result of an ifosfamide-dependent drug–drug interaction: results of a phase I study. *Br. J. Cancer* **110**, 888–893 (2014).

143. He, H. *et al.* Midostaurin, a Novel Protein Kinase Inhibitor for the Treatment of Acute Myelogenous Leukemia: Insights from Human Absorption, Metabolism, and Excretion Studies of a BDDCS II Drug. *Drug Metab. Dispos.* **45**, 540–555 (2017).

144. Hwang, J. J. *et al.* Phase I Dose Finding Studies of Obatoclax (GX15-070), a Small Molecule Pan-BCL-2 Family Antagonist, in Patients with Advanced Solid Tumors or Lymphoma. *Clin. Cancer Res. Off. J. Am. Assoc. Cancer Res.* **16**, 4038–4045 (2010).

145. Isah, A. O., Rawlins, M. D. & Bateman, D. N. Clinical pharmacology of prochlorperazine in healthy young males. *Br. J. Clin. Pharmacol.* **32**, 677–684 (1991).

146. Kisanga, E. R. *et al.* Tamoxifen and Metabolite Concentrations in Serum and Breast Cancer Tissue during Three Dose Regimens in a Randomized Preoperative Trial. *Clin. Cancer Res.* **10**, 2336–2343 (2004).

147. Kitzen, J. J. E. M. *et al.* Phase I dose-escalation study of F60008, a novel apoptosis inducer, in patients with advanced solid tumours. *Eur. J. Cancer* **45**, 1764–1772 (2009).

148. Kosoglou, T. *et al.* Pharmacodynamics and pharmacokinetics of the novel PAR-1 antagonist vorapaxar (formerly SCH 530348) in healthy subjects. *Eur. J. Clin. Pharmacol.* **68**, 249–258 (2012).

149. Kuhn, J. G. *et al.* Pharmacokinetic and Tumor Distribution Characteristics of Temsirolimus in Patients with Recurrent Malignant Glioma. *Clin. Cancer Res. Off. J. Am. Assoc. Cancer Res.* **13**, 7401–7406 (2007).

150. Leijen, S. *et al.* A phase I, open-label, randomized crossover study to assess the effect of dosing of the MEK 1/2 inhibitor Selumetinib (AZD6244; ARRY-142866) in the presence and absence of food in patients with advanced solid tumors. *Cancer Chemother. Pharmacol.* **68**, 1619–1628 (2011).

151. Liu, Y.-M. *et al.* Pharmacokinetic Properties and Bioequivalence of Two Sulfadoxine/Pyrimethamine Fixed-Dose Combination Tablets: A Parallel-Design Study in Healthy Chinese Male Volunteers. *Clin. Ther.* **34**, 2212–2220 (2012).

152. LoConte, N. K. *et al.* A Multicenter Phase 1 Study of γ -secretase inhibitor RO4929097 in Combination with Capecitabine in Refractory Solid Tumors. *Invest. New Drugs* **33**, 169–176 (2015).

153. Lu, K., Yap, H.-Y. & Loo, T. L. Clinical Pharmacokinetics of Vinblastine by Continuous Intravenous Infusion. *Cancer Res.* **43**, 1405–1408 (1983).

154. Melichar, B. *et al.* Clinical activity of patupilone in patients with pretreated advanced/metastatic colon cancer: results of a phase I dose escalation trial. *Br. J. Cancer* **105**, 1646–1653 (2011).

155. Midha, K. K. *et al.* Kinetics of oral trifluoperazine disposition in man. *Br. J. Clin. Pharmacol.* **15**, 380–382 (1983).

156. Mross, K. *et al.* A Phase I Dose–Escalation Study of Regorafenib (BAY 73–4506), an Inhibitor of Oncogenic, Angiogenic, and Stromal Kinases, in Patients with Advanced Solid Tumors. *Clin. Cancer Res.* **18**, 2658–2667 (2012).

157. Muralidharan, G., Micalizzi, M., Speth, J., Raible, D. & Troy, S. Pharmacokinetics of Tigecycline after Single and Multiple Doses in Healthy Subjects. *Antimicrob. Agents Chemother.* **49**, 220–229 (2005).

158. Narasimhan, N. I., Dorer, D. J., Niland, K., Haluska, F. & Sonnichsen, D. Effects of Ketoconazole on the Pharmacokinetics of Ponatinib in Healthy Subjects. *J. Clin. Pharmacol.* **53**, 974–981 (2013).

159. Nichols, D. J., Muirhead, G. J. & Harness, J. A. Pharmacokinetics of sildenafil after single oral doses in healthy male subjects: absolute bioavailability, food effects and dose proportionality. *Br. J. Clin. Pharmacol.* **53**, 5S-12S (2002).

160. Nijenhuis, C. M. *et al.* Pharmacokinetics and excretion of 14C-omacetaxine in patients with advanced solid tumors. *Invest. New Drugs* **34**, 565–574 (2016).

161. Patnaik, A. *et al.* A Phase I, Pharmacokinetic, and Biological Study of the Farnesyltransferase Inhibitor Tipifarnib in Combination with Gemcitabine in Patients with Advanced Malignancies. *Clin. Cancer Res.* **9**, 4761–4771 (2003).

162. Prakash, S. *et al.* Chronic Oral Administration of CI-994: A Phase I Study. *Invest. New Drugs* **19**, 1–11 (2001).

163. Ramalingam, S. S. *et al.* Phase I and Pharmacokinetic Study of Vorinostat, A Histone Deacetylase Inhibitor, in Combination with Carboplatin and Paclitaxel for Advanced Solid Malignancies. *Clin. Cancer Res.* **13**, 3605–3610 (2007).

164. Richardson, P. G. *et al.* Tanespimycin monotherapy in relapsed multiple myeloma: results of a phase 1 dose-escalation study. *Br. J. Haematol.* **150**, 438–445 (2010).

165. Sandmaier, B. M. *et al.* Results of a phase 1 study of quizartinib as maintenance therapy in subjects with acute myeloid leukemia in remission following allogeneic hematopoietic stem cell transplant. *Am. J. Hematol.* **93**, 222–231 (2018).

166. Savelieva, M. *et al.* Population pharmacokinetics of intravenous and oral panobinostat in patients with hematologic and solid tumors. *Eur. J. Clin. Pharmacol.* **71**, 663–672 (2015).

167. Schilcher, R. B., Young, J. D., Ratanatharathorn, V., Karanes, C. & Baker, L. H. Clinical pharmacokinetics of high-dose mitomycin C. *Cancer Chemother. Pharmacol.* **13**, 186–190 (1984).

168. Schweizer, M. T. *et al.* A phase I study of niclosamide in combination with enzalutamide in men with castration-resistant prostate cancer. *PLoS ONE* **13**, e0198389 (2018).

169. Selden, R., Smith, T. W. & Findley, W. Ouabain Pharmacokinetics in Dog and Man. *Circulation* **45**, 1176–1182 (1972).

170. Sethi, V. S. *et al.* Pharmacokinetics of Vincristine Sulfate in Adult Cancer Patients. *Cancer Res.* **41**, 3551–3555 (1981).

171. Shapiro, G. I. *et al.* Pharmacokinetic Study of Rucaparib in Patients With Advanced Solid Tumors. *Clin. Pharmacol. Drug Dev.* **8**, 107–118 (2019).

172. Sikma, M. A. *et al.* Pharmacokinetics and Toxicity of Tacrolimus Early After Heart and Lung Transplantation: Tacrolimus Pharmacokinetics Posttransplant. *Am. J. Transplant.* **15**, 2301–2313 (2015).

173. Tamura, K. *et al.* Phase I study of palbociclib, a cyclin-dependent kinase 4/6 inhibitor, in Japanese patients. *Cancer Sci.* **107**, 755–763 (2016).

174. Tanaka, C. *et al.* Clinical Pharmacokinetics of the BCR–ABL Tyrosine Kinase Inhibitor Nilotinib. *Clin. Pharmacol. Ther.* **87**, 197–203 (2010).

175. Tomkinson, H. *et al.* Pharmacokinetics and tolerability of zibotentan (ZD4054) in subjects with hepatic or renal impairment: two open-label comparative studies. *BMC Clin. Pharmacol.* **11**, 3 (2011).

176. Verstovsek, S., Yeleswaram, S., Hou, K., Chen, X. & Erickson-Viitanen, S. Sustained-release ruxolitinib: Findings from a phase 1 study in healthy subjects and a phase 2 study in patients with myelofibrosis. *Hematol. Oncol.* **36**, 701–708 (2018).

177. Willis, B. A. *et al.* Semagacestat Pharmacokinetics Are Not Significantly Affected by Formulation, Food, or Time of Dosing in Healthy Participants. *J. Clin. Pharmacol.* **52**, 904–913 (2012).

178. Wilson, W. H. *et al.* Safety, Pharmacokinetics, Pharmacodynamics, and Activity of Navitoclax, a Targeted High Affinity Inhibitor of BCL-2, in Lymphoid Malignancies. *Lancet Oncol.* **11**, 1149–1159 (2010).

179. Xin, Y. *et al.* The Relative Bioavailability, Food Effect, and Drug Interaction With Omeprazole of Momelotinib Tablet Formulation in Healthy Subjects. *Clin. Pharmacol. Drug Dev.* **7**, 277–286 (2018).

180. Albain, K. S. *et al.* A Randomized Trial of Adjuvant Chemotherapy and Tamoxifen Timing in Postmenopausal, Endocrine-Responsive, Node-Positive Breast Cancer. *Lancet* **374**, 2055–2063 (2009).

181. Araujo, J. C. *et al.* Docetaxel and dasatinib or placebo in men with metastatic castration-resistant prostate cancer (READY): a randomised, double-blind phase 3 trial. *Lancet Oncol.* **14**, 1307–1316 (2013).

182. Bajetta, E. *et al.* Randomized trial on adjuvant treatment with FOLFIRI followed by docetaxel and cisplatin versus 5-fluorouracil and folinic acid for radically resected gastric cancer. *Ann. Oncol.* **25**, 1373–1378 (2014).

183. Bear, H. D. *et al.* Sequential Preoperative or Postoperative Docetaxel Added to Preoperative Doxorubicin Plus Cyclophosphamide for Operable Breast Cancer: National Surgical Adjuvant Breast and Bowel Project Protocol B-27. *J. Clin. Oncol.* **24**, 2019–2027 (2006).

184. Bellmunt, J. *et al.* Randomized Phase III Study Comparing Paclitaxel/Cisplatin/ Gemcitabine and Gemcitabine/Cisplatin in Patients With Locally Advanced or Metastatic Urothelial Cancer Without Prior Systemic Therapy: EORTC Intergroup Study 30987. *J. Clin. Oncol.* **30**, 1107–1113 (2012).

185. du Bois, A. *et al.* Phase III trial of carboplatin plus paclitaxel with or without gemcitabine in first-line treatment of epithelial ovarian cancer. *J. Clin. Oncol. Off. J. Am. Soc. Clin. Oncol.* **28**, 4162–4169 (2010).

186. Carrato, A. *et al.* Fluorouracil, Leucovorin, and Irinotecan Plus Either Sunitinib or Placebo in Metastatic Colorectal Cancer: A Randomized, Phase III Trial. *J. Clin. Oncol.* **31**, 1341–1347 (2013).

187. Cavo, M. *et al.* Bortezomib-thalidomide-dexamethasone is superior to thalidomide-dexamethasone as consolidation therapy after autologous hematopoietic stem cell transplantation in patients with newly diagnosed multiple myeloma. *Blood* **120**, 9–19 (2012).

188. Colucci, G. *et al.* Randomized Phase III Trial of Gemcitabine Plus Cisplatin Compared With Single-Agent Gemcitabine As First-Line Treatment of Patients With Advanced Pancreatic Cancer: The GIP-1 Study. *J. Clin. Oncol.* **28**, 1645–1651 (2010).

189. Di Leo, A. *et al.* Phase III, Double-Blind, Randomized Study Comparing Lapatinib Plus Paclitaxel With Placebo Plus Paclitaxel As First-Line Treatment for Metastatic Breast Cancer. *J. Clin. Oncol.* **26**, 5544–5552 (2008).

190. Douillard, J. *et al.* Irinotecan combined with fluorouracil compared with fluorouracil alone as first-line treatment for metastatic colorectal cancer: a multicentre randomised trial. *The Lancet* **355**, 1041–1047 (2000).

191. Dueñas-González, A. *et al.* Phase III, Open-Label, Randomized Study Comparing Concurrent Gemcitabine Plus Cisplatin and Radiation Followed by Adjuvant Gemcitabine and Cisplatin Versus Concurrent Cisplatin and Radiation in Patients With Stage IIB to IVA Carcinoma of the Cervix. *J. Clin. Oncol.* **29**, 1678–1685 (2011).

192. Falcone, A. *et al.* Phase III Trial of Infusional Fluorouracil, Leucovorin, Oxaliplatin, and Irinotecan (FOLFOXIRI) Compared With Infusional Fluorouracil, Leucovorin, and Irinotecan (FOLFIRI) As First-Line Treatment for Metastatic Colorectal Cancer: The Gruppo Oncologico Nord Ovest. *J. Clin. Oncol.* **25**, 1670–1676 (2007).

193. Flaherty, K. T. *et al.* Phase III Trial of Carboplatin and Paclitaxel With or Without Sorafenib in Metastatic Melanoma. *J. Clin. Oncol.* **31**, 373–379 (2013).

194. Gatzemeier, U. *et al.* Phase III Study of Erlotinib in Combination With Cisplatin and Gemcitabine in Advanced Non–Small-Cell Lung Cancer: The Tarceva Lung Cancer Investigation Trial. *J. Clin. Oncol.* **25**, 1545–1552 (2007).

195. Giaccone, G. *et al.* Gefitinib in Combination With Gemcitabine and Cisplatin in Advanced Non–Small-Cell Lung Cancer: A Phase III Trial—INTACT 1. *J. Clin. Oncol.* **22**, 777–784 (2004).

196. Gianni, L. *et al.* Phase III Trial Evaluating the Addition of Paclitaxel to Doxorubicin Followed by Cyclophosphamide, Methotrexate, and Fluorouracil, As Adjuvant or Primary Systemic Therapy: European Cooperative Trial in Operable Breast Cancer. *J. Clin. Oncol.* **27**, 2474–2481 (2009).

197. Gill, S. *et al.* PANCREOX: A Randomized Phase III Study of Fluorouracil/Leucovorin With or Without Oxaliplatin for Second-Line Advanced Pancreatic Cancer in Patients Who Have Received Gemcitabine-Based Chemotherapy. *J. Clin. Oncol.* **34**, 3914–3920 (2016).

198. Haller, D. G. *et al.* Oxaliplatin Plus Irinotecan Compared With Irinotecan Alone as Second-Line Treatment After Single-Agent Fluoropyrimidine Therapy for Metastatic Colorectal Carcinoma. *J. Clin. Oncol.* **26**, 4544–4550 (2008).

199. Hanna, N. *et al.* Phase III Study of Cisplatin, Etoposide, and Concurrent Chest Radiation With or Without Consolidation Docetaxel in Patients With Inoperable Stage III Non–Small-Cell Lung Cancer: The Hoosier Oncology Group and U.S. Oncology. *J. Clin. Oncol.* **26**, 5755–5760 (2008).

200. Hauschild, A. *et al.* Results of a Phase III, Randomized, Placebo-Controlled Study of Sorafenib in Combination With Carboplatin and Paclitaxel As Second-Line Treatment in Patients With Unresectable Stage III or Stage IV Melanoma. *J. Clin. Oncol.* **27**, 2823–2830 (2009).

201. Herbst, R. S. *et al.* Gefitinib in Combination With Paclitaxel and Carboplatin in Advanced Non–Small-Cell Lung Cancer: A Phase III Trial—INTACT 2. *J. Clin. Oncol.* **22**, 785–794 (2004).

202. Herbst, R. S. *et al.* TRIBUTE: A Phase III Trial of Erlotinib Hydrochloride (OSI-774) Combined With Carboplatin and Paclitaxel Chemotherapy in Advanced Non–Small-Cell Lung Cancer. *J. Clin. Oncol.* **23**, 5892–5899 (2005).

203. Herbst, R. S. *et al.* Vandetanib plus docetaxel versus docetaxel as second-line treatment for patients with advanced non-small-cell lung cancer (ZODIAC): a double-blind, randomised, phase 3 trial. *Lancet Oncol.* **11**, 619–626 (2010).

204. Homesley, H. D. *et al.* Phase III Trial of Ifosfamide With or Without Paclitaxel in Advanced Uterine Carcinosarcoma: A Gynecologic Oncology Group Study. *J. Clin. Oncol.* **25**, 526–531 (2007).

205. Kindler, H. L. *et al.* Axitinib plus gemcitabine versus placebo plus gemcitabine in patients with advanced pancreatic adenocarcinoma: a double-blind randomised phase 3 study. *Lancet Oncol.* **12**, 256–262 (2011).

206. Köhne, C.-H. *et al.* Phase III Study of Weekly High-Dose Infusional Fluorouracil Plus Folinic Acid With or Without Irinotecan in Patients With Metastatic Colorectal Cancer: European Organisation for Research and Treatment of Cancer Gastrointestinal Group Study 40986. *J. Clin. Oncol.* **23**, 4856–4865 (2005).

207. Kubota, K. *et al.* Etoposide and cisplatin versus irinotecan and cisplatin in patients with limited-stage small-cell lung cancer treated with etoposide and cisplatin plus concurrent accelerated hyperfractionated thoracic radiotherapy (JCOG0202): a randomised phase 3 study. *Lancet Oncol.* **15**, 106–113 (2014).

208. Kudo, M. *et al.* Sorafenib plus low-dose cisplatin and fluorouracil hepatic arterial infusion chemotherapy versus sorafenib alone in patients with advanced hepatocellular carcinoma (SILIUS): a randomised, open label, phase 3 trial. *Lancet Gastroenterol. Hepatol.* **3**, 424–432 (2018).

209. Lee, J. *et al.* Gemcitabine and oxaliplatin with or without erlotinib in advanced biliary-tract cancer: a multicentre, open-label, randomised, phase 3 study. *Lancet Oncol.* **13**, 181–188 (2012).

210. Lee, S. M. *et al.* Anti-angiogenic Therapy Using Thalidomide Combined With Chemotherapy in Small Cell Lung Cancer: A Randomized, Double-Blind, Placebo-Controlled Trial. *JNCI J. Natl. Cancer Inst.* **101**, 1049–1057 (2009).

211. Lilenbaum, R. C. *et al.* Single-Agent Versus Combination Chemotherapy in Advanced Non–Small-Cell Lung Cancer: The Cancer and Leukemia Group B (study 9730). *J. Clin. Oncol.* **23**, 190–196 (2005).

212. Martín, M. *et al.* Gemcitabine plus vinorelbine versus vinorelbine monotherapy in patients with metastatic breast cancer previously treated with anthracyclines and taxanes: final results of the phase III Spanish Breast Cancer Research Group (GEICAM) trial. *Lancet Oncol.* **8**, 219–225 (2007).

213. Moore, M. J. *et al.* Erlotinib plus gemcitabine compared with gemcitabine alone in patients with advanced pancreatic cancer: a phase III trial of the National Cancer Institute of Canada Clinical Trials Group. *J. Clin. Oncol. Off. J. Am. Soc. Clin. Oncol.* **25**, 1960–1966 (2007).

214. Morabito, A. *et al.* Randomized phase III trial of gemcitabine and cisplatin vs. gemcitabine alone in patients with advanced non-small cell lung cancer and a performance status of 2: the CAPPA-2 study. *Lung Cancer Amst. Neth.* **81**, 77–83 (2013).

215. Moreau, P. *et al.* Bortezomib plus dexamethasone versus reduced-dose bortezomib, thalidomide plus dexamethasone as induction treatment before autologous stem cell transplantation in newly diagnosed multiple myeloma. *Blood* **118**, 5752–5758 (2011).

216. Muggia, F. M. *et al.* Phase III Randomized Study of Cisplatin Versus Paclitaxel Versus Cisplatin and Paclitaxel in Patients With Suboptimal Stage III or IV Ovarian Cancer: A Gynecologic Oncology Group Study. *J. Clin. Oncol.* **18**, 106–106 (2000).

217. Niesvizky, R. *et al.* Community-Based Phase IIIB Trial of Three UPFRONT Bortezomib-Based Myeloma Regimens. *J. Clin. Oncol.* **33**, 3921–3929 (2015).

218. O'Neil, B. H. *et al.* A phase II/III randomized study to compare the efficacy and safety of rigosertib plus gemcitabine versus gemcitabine alone in patients with previously untreated metastatic pancreatic cancer. *Ann. Oncol.* **26**, 1923–1929 (2015).

219. Orlowski, R. Z. *et al.* Randomized Phase III Study of Pegylated Liposomal Doxorubicin Plus Bortezomib Compared With Bortezomib Alone in Relapsed or Refractory Multiple Myeloma: Combination Therapy Improves Time to Progression. *J. Clin. Oncol.* **25**, 3892–3901 (2007).

220. O'Shaughnessy, J. *et al.* Superior Survival With Capecitabine Plus Docetaxel Combination Therapy in Anthracycline-Pretreated Patients With Advanced Breast Cancer: Phase III Trial Results. *J. Clin. Oncol.* **20**, 2812–2823 (2002).

221. Paz-Ares, L. G. *et al.* Phase III, Randomized, Double-Blind, Placebo-Controlled Trial of Gemcitabine/Cisplatin Alone or With Sorafenib for the First-Line Treatment of Advanced, Nonsquamous Non–Small-Cell Lung Cancer. *J. Clin. Oncol.* **30**, 3084–3092 (2012).

222. Perilongo, G. *et al.* Cisplatin versus Cisplatin plus Doxorubicin for Standard-Risk Hepatoblastoma. *N. Engl. J. Med.* **361**, 1662–1670 (2009).

223. Pfisterer, J. *et al.* Randomized Phase III Trial of Topotecan Following Carboplatin and Paclitaxel in First-line Treatment of Advanced Ovarian Cancer: A Gynecologic Cancer Intergroup Trial of the AGO-OVAR and GINECO. *JNCI J. Natl. Cancer Inst.* **98**, 1036–1045 (2006).

224. Rosiñol, L. *et al.* Superiority of bortezomib, thalidomide, and dexamethasone (VTD) as induction pretransplantation therapy in multiple myeloma: a randomized phase 3 PETHEMA/GEM study. *Blood* **120**, 1589–1596 (2012).

225. San-Miguel, J. F. *et al.* Overall survival of patients with relapsed multiple myeloma treated with panobinostat or placebo plus bortezomib and dexamethasone (the PANORAMA 1 trial): a randomised, placebo-controlled, phase 3 trial. *Lancet Haematol.* **3**, e506–e515 (2016).

226. Scagliotti, G. *et al.* Phase III Study of Carboplatin and Paclitaxel Alone or With Sorafenib in Advanced Non–Small-Cell Lung Cancer. *J. Clin. Oncol.* **28**, 1835–1842 (2010).

227. Scagliotti, G. *et al.* Phase III Multinational, Randomized, Double-Blind, Placebo-Controlled Study of Tivantinib (ARQ 197) Plus Erlotinib Versus Erlotinib Alone in Previously Treated Patients With Locally Advanced or Metastatic Nonsquamous Non–Small-Cell Lung Cancer. *J. Clin. Oncol.* **33**, 2667–2674 (2015).

228. Swain, S. M. *et al.* Definitive Results of a Phase III Adjuvant Trial Comparing Three Chemotherapy Regimens in Women With Operable, Node-Positive Breast Cancer: The NSABP B-38 Trial. *J. Clin. Oncol.* **31**, 3197–3204 (2013).

229. The ICON and AGO Collaborators. Paclitaxel plus platinum-based chemotherapy versus conventional platinum-based chemotherapy in women with relapsed ovarian cancer: the ICON4/AGO-OVAR-2.2 trial. *The Lancet* **361**, 2099–2106 (2003).

230. Tsukada, H. *et al.* Randomized controlled trial comparing docetaxel–cisplatin combination with weekly docetaxel alone in elderly patients with advanced non-small-cell lung cancer: Japan Clinical Oncology Group (JCOG) 0207. *Jpn. J. Clin. Oncol.* **45**, 88–95 (2015).

231. Valle, J. *et al.* Cisplatin plus gemcitabine versus gemcitabine for biliary tract cancer. *N. Engl. J. Med.* **362**, 1273–1281 (2010).

232. Van Cutsem, E. *et al.* Phase III Study of Docetaxel and Cisplatin Plus Fluorouracil Compared With Cisplatin and Fluorouracil As First-Line Therapy for Advanced Gastric Cancer: A Report of the V325 Study Group. *J. Clin. Oncol.* **24**, 4991–4997 (2006).

233. Van Cutsem, E. *et al.* Randomized Phase III Trial Comparing Biweekly Infusional Fluorouracil/Leucovorin Alone or With Irinotecan in the Adjuvant Treatment of Stage III Colon Cancer: PETACC-3. *J. Clin. Oncol.* **27**, 3117–3125 (2009).

234. Vermorken, J. B. *et al.* Cisplatin, Fluorouracil, and Docetaxel in Unresectable Head and Neck Cancer. *N. Engl. J. Med.* **357**, 1695–1704 (2007).

235. Von Hoff, D. D. *et al.* Increased Survival in Pancreatic Cancer with nab-Paclitaxel plus Gemcitabine. *N. Engl. J. Med.* **369**, 1691–1703 (2013).

236. Wu, Y.-L. *et al.* Intercalated combination of chemotherapy and erlotinib for patients with advanced stage non-small-cell lung cancer (FASTACT-2): a randomised, double-blind trial. *Lancet Oncol.* **14**, 777–786 (2013).

237. Yoshioka, H. *et al.* A randomized, double-blind, placebo-controlled, phase III trial of erlotinib with or without a c-Met inhibitor tivantinib (ARQ 197) in Asian patients with previously treated stage IIIB/IV nonsquamous nonsmall-cell lung cancer harboring wild-type epidermal growth factor receptor (ATTENTION study). *Ann. Oncol.* **26**, 2066–2072 (2015).

238. Adler, D. *et al. rgl: 3D Visualization Using OpenGL*. (2017).

239. Csárdi, G. & FitzJohn, R. *progress: Terminal Progress Bars*. (2016).

240. Dragulescu, A. A. *xlsx: Read, write, format Excel 2007 and Excel 97/2000/XP/2003 files*. (2014).

241. Fox, J. & Weisberg, S. *An R Companion to Applied Regression*. (SAGE Publications, 2011).

242. Gu, Z., Eils, R. & Schlesner, M. Complex heatmaps reveal patterns and correlations in multidimensional genomic data. *Bioinformatics* **32**, 2847–2849 (2016).

243. Neuwirth, E. *RColorBrewer: ColorBrewer Palettes*. (2014).

244. Qiu, W., Chavarro, J., Lazarus, R., Rosner, B. & Ma, J. *powerSurvEpi: Power and Sample Size Calculation for Survival Analysis of Epidemiological Studies*. (2015).

245. R Core Team. *R: A Language and Environment for Statistical Computing*. (R Foundation for Statistical Computing, 2017).

246. Ritz, C., Baty, F., Streibig, J. C. & Gerhard, D. Dose-Response Analysis Using R. *PLoS ONE* **10**, e0146021 (2015).

247. Saito, T. & Rehmsmeier, M. Precrec: fast and accurate precision–recall and ROC curve calculations in R. *Bioinformatics* **33**, 145–147 (2017).

248. Schauberger, P. & Walker, A. *openxlsx: Read, Write and Edit xlsx Files*. (2019).

249. Solymos, P. & Zawadzki, Z. *pbapply: Adding Progress Bar to '*apply' Functions*. (2017).

250. Wickham, H. *rvest: Easily Harvest (Scrape) Web Pages*. (2016).

251. Wickham, H. & Bryan, J. *readxl: Read Excel Files*. (2017).

252. Wickham, H., Hester, J. & Francois, R. *readr: Read Rectangular Text Data*. (2017).

253. Zeileis, A. Econometric Computing with HC and HAC Covariance Matrix Estimators. *J. Stat. Softw.* **11**, 1–17 (2004).

254. Zeileis, A. Object-oriented Computation of Sandwich Estimators. *J. Stat. Softw.* **16**, 1–16 (2006).
