# Supplementary material for: Computationally predicting clinical drug combination efficacy with cancer cell line screens and independent drug action
Source: Nat Commun. 2020 Nov 17;11:5848. doi: 10.1038/s41467-020-19563-6 (PMC7673995; doi:10.1038/s41467-020-19563-6)
Supplement: Supplementary file 3 — Description of Additional Supplementary Files [file 41467_2020_19563_MOESM3_ESM.pdf]

Description of Additional Supplementary Files

Title: “Supplementary Data 1_NCI-ALMANAC_Predicted_vs_Measured_Viabilities.xlsx”

Description: An excel table with two sheets containing the measured and predicted drug combination viabilities for NCI-ALMANAC used to generate Figure 2. The “ALMANAC_Prediction_vs_Measured” sheet contains the predicted and measured viability values and the “Column Descriptions” sheet contains descriptions for each column in the first sheet.

Title: “Supplementary Data 2_drug by drug NCI-ALMANAC validation.zip”

Description: A zip file containing a large PDF with 237 plots showing the relationship between predicted and measured mean % viability for combinations in NCI-ALMANAC, with each plot being specific to combinations containing the drug in the title of the plot. Each plot is a subset of the data plotted in Figure 2a in the main text and is provided in this drug-by-drug format to enable researchers to easily look up how well IDACombo preforms for their particular drug(s) of interest in NCI-ALMANAC.

Title: “Supplementary Data 3_Clinical Trial Predictions.xlsx”

Description: An excel table with two sheets containing information about the clinical trials used in the IDACombo clinical trial validation analysis as well as the predicted treatment efficacies, HRs, and powers for each trial. The “Clinical_Trial_Predictions” sheet contains the clinical trial information and the “Column Descriptions” sheet contains definitions for each column in the “Clinical_Trial_Predictions” sheet. This table relates to Figures 4, S5, and S6.

Title: “Supplementary Data 4_Clinical Drug Concentrations.xlsx”

Description: An excel table with two sheets containing Cmax and Csustained drug concentrations as well as concentration source citations for the drugs and doses used in the clinical trial analysis and the prospective analysis. The “Clinical Trial Conc.” sheet contains information for the clinical trial analysis, and the “Prospective Analysis Conc.” sheet contains information for the prospective analysis. This table relates to Figures 4-6 and S5-S11.

Title: “Supplementary Data 5_Targeted Therapy Predictions.xlsx”

Description: An excel table with one sheet detailing IDACombo predictions for two trials which tested targeted therapies in patients who did and did not contain the molecular features targeted by those therapies. IDACombo predictions are also presented when made with and without cell lines that carry the targeted molecular features.

Title: “Supplementary Data 6_GDSC_CTRP_Agreement_vs_n_Cell_Lines_and_Cancer_Type.xlsx”

Description: An excel table with two sheets containing information about the agreement between predictions made using CTRPv2 and those made using GDSC for various cancer types/subtypes. The “Correlation By Cancer Type” sheet contains information about the prediction agreement and the number of cell lines available for each cancer type. The “Column Descriptions” sheet contains definitions for each column in the “Correlation By Cancer Type” sheet. This table relates to Figure S10B.
