# Supplementary material for: Computationally predicting clinical drug combination efficacy with cancer cell line screens and independent drug action
Source: Nat Commun. 2020 Nov 17;11:5848. doi: 10.1038/s41467-020-19563-6 (PMC7673995; doi:10.1038/s41467-020-19563-6)

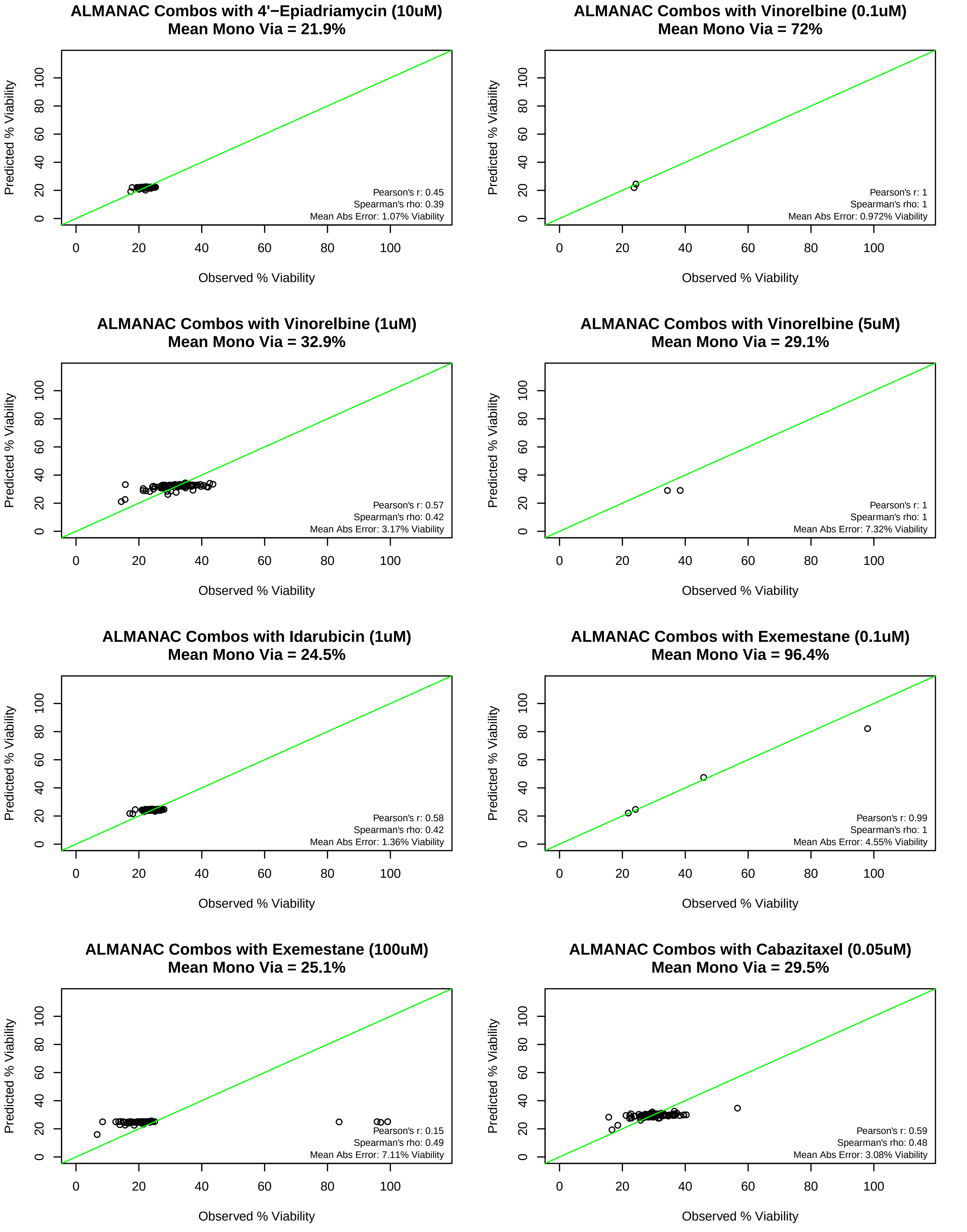
ALMANAC Combos with 4'−Epiadriamycin (10uM)
Mean Mono Via = 21.9%
Pearson's r: 0.45
Spearman's rho: 0.39
Mean Abs Error: 1.07% Viability
ALMANAC Combos with Vinorelbine (0.1uM)
Mean Mono Via = 72%
Pearson's r: 1
Spearman's rho: 1
Mean Abs Error: 0.972% Viability
ALMANAC Combos with Vinorelbine (1uM)
Mean Mono Via = 32.9%
Pearson's r: 0.57
Spearman's rho: 0.42
Mean Abs Error: 3.17% Viability
ALMANAC Combos with Vinorelbine (5uM)
Mean Mono Via = 29.1%
Pearson's r: 1
Spearman's rho: 1
Mean Abs Error: 7.32% Viability
ALMANAC Combos with Idarubicin (1uM)
Mean Mono Via = 24.5%
Pearson's r: 0.58
Spearman's rho: 0.42
Mean Abs Error: 1.36% Viability
ALMANAC Combos with Exemestane (0.1uM)
Mean Mono Via = 96.4%
Pearson's r: 0.99
Spearman's rho: 1
Mean Abs Error: 4.55% Viability
ALMANAC Combos with Exemestane (100uM)
Mean Mono Via = 25.1%
Pearson's r: 0.15
Spearman's rho: 0.49
Mean Abs Error: 7.11% Viability
ALMANAC Combos with Cabazitaxel (0.05uM)
Mean Mono Via = 29.5%
Pearson's r: 0.59
Spearman's rho: 0.48
Mean Abs Error: 3.08% Viability
Predicted % Viability
Observed % Viability
0
20
40
60
80
100

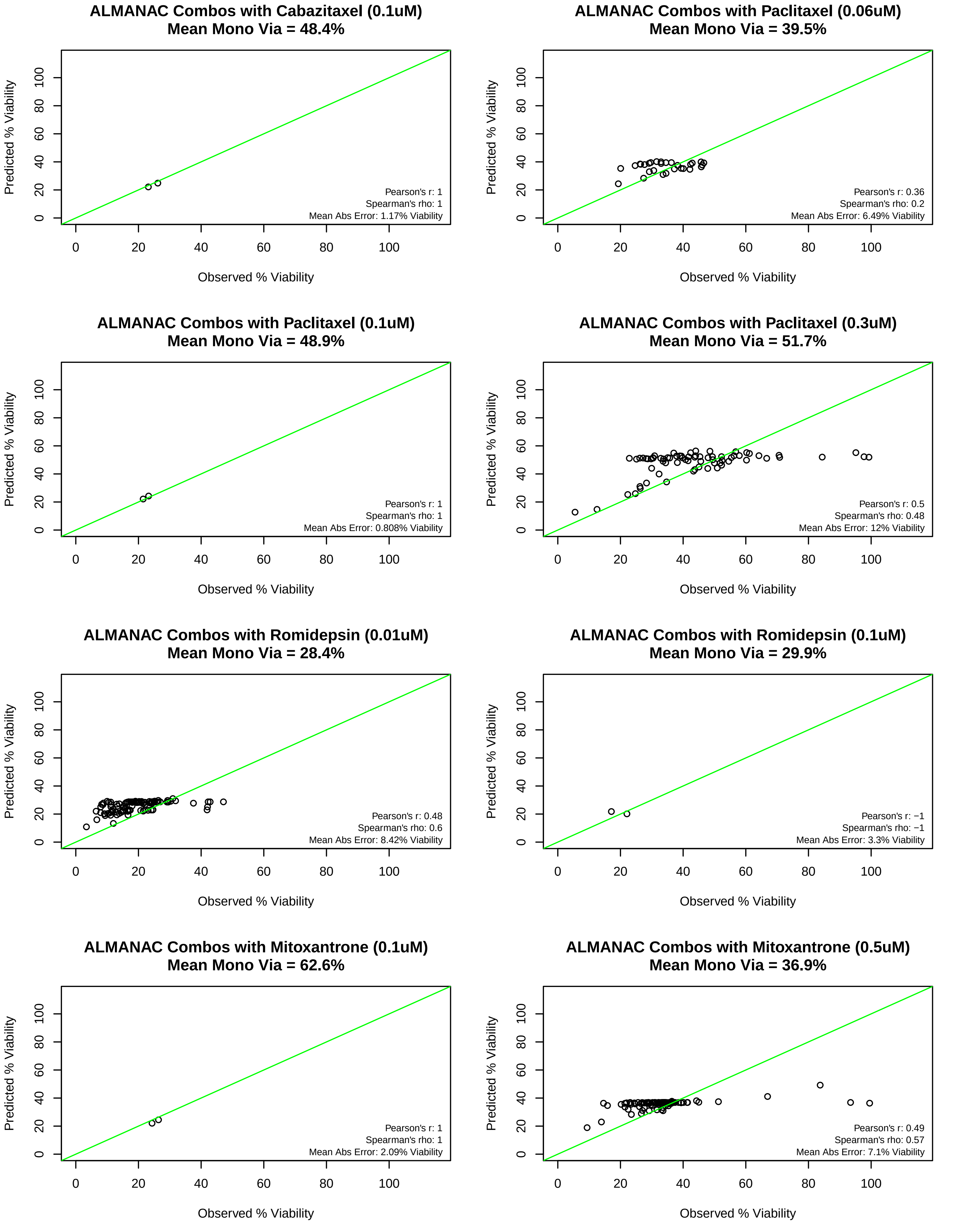
ALMANAC Combos with Cabazitaxel (0.1uM)
Mean Mono Via = 48.4%
Pearson's r: 1
Spearman's rho: 1
Mean Abs Error: 1.17% Viability
ALMANAC Combos with Paclitaxel (0.06uM)
Mean Mono Via = 39.5%
Pearson's r: 0.36
Spearman's rho: 0.2
Mean Abs Error: 6.49% Viability
ALMANAC Combos with Paclitaxel (0.1uM)
Mean Mono Via = 48.9%
Pearson's r: 1
Spearman's rho: 1
Mean Abs Error: 0.808% Viability
ALMANAC Combos with Paclitaxel (0.3uM)
Mean Mono Via = 51.7%
Pearson's r: 0.5
Spearman's rho: 0.48
Mean Abs Error: 12% Viability
ALMANAC Combos with Romidepsin (0.01uM)
Mean Mono Via = 28.4%
Pearson's r: 0.48
Spearman's rho: 0.6
Mean Abs Error: 8.42% Viability
ALMANAC Combos with Romidepsin (0.1uM)
Mean Mono Via = 29.9%
Pearson's r: −1
Spearman's rho: −1
Mean Abs Error: 3.3% Viability
ALMANAC Combos with Mitoxantrone (0.1uM)
Mean Mono Via = 62.6%
Pearson's r: 1
Spearman's rho: 1
Mean Abs Error: 2.09% Viability
ALMANAC Combos with Mitoxantrone (0.5uM)
Mean Mono Via = 36.9%
Pearson's r: 0.49
Spearman's rho: 0.57
Mean Abs Error: 7.1% Viability
Predicted % Viability
Observed % Viability
0
20
40
60
80
100

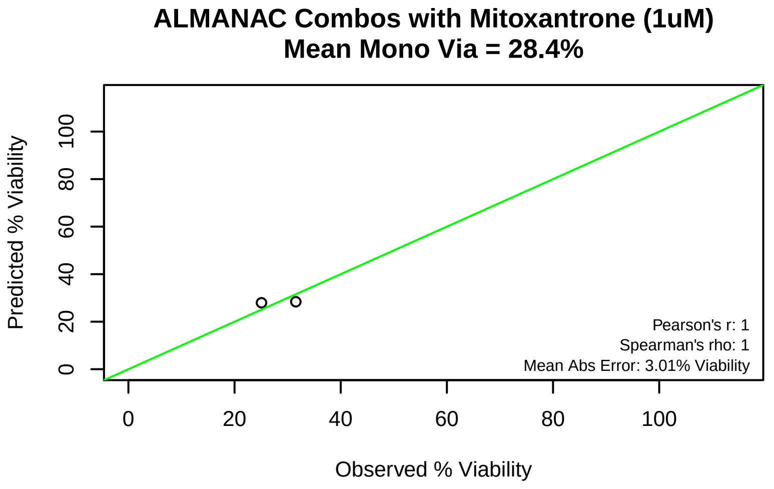
ALMANAC Combos with Mitoxantrone (1uM)
Mean Mono Via = 28.4%
Pearson's r: 1
Spearman's rho: 1
Mean Abs Error: 3.01% Viability
Predicted % Viability
Observed % Viability

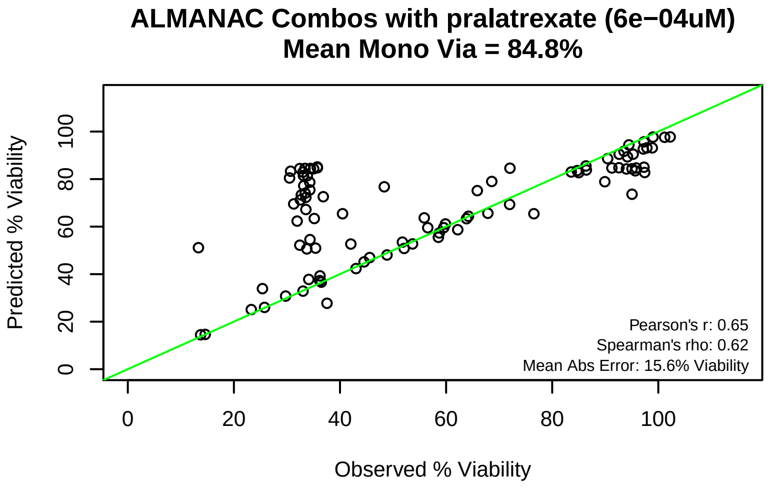
ALMANAC Combos with pralatrexate (6e−04uM)
Mean Mono Via = 84.8%
Pearson's r: 0.65
Spearman's rho: 0.62
Mean Abs Error: 15.6% Viability
Predicted % Viability
Observed % Viability

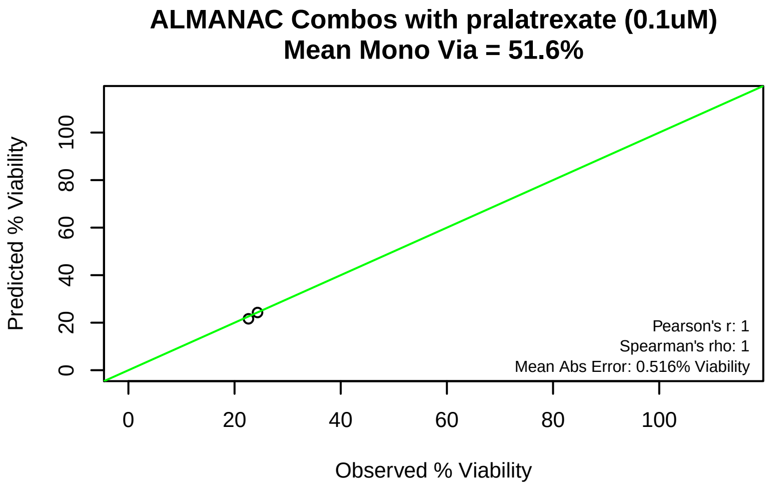
ALMANAC Combos with pralatrexate (0.1uM)
Mean Mono Via = 51.6%
Pearson's r: 1
Spearman's rho: 1
Mean Abs Error: 0.516% Viability
Predicted % Viability
Observed % Viability

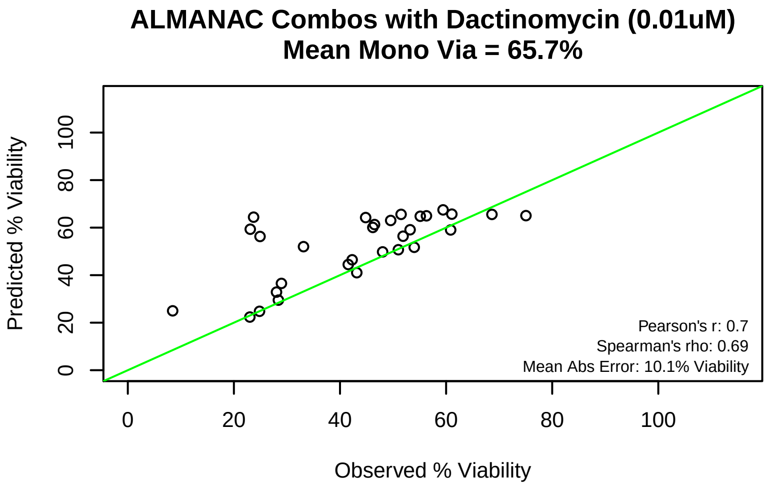
ALMANAC Combos with Dactinomycin (0.01uM)
Mean Mono Via = 65.7%
Pearson's r: 0.7
Spearman's rho: 0.69
Mean Abs Error: 10.1% Viability
Predicted % Viability
Observed % Viability

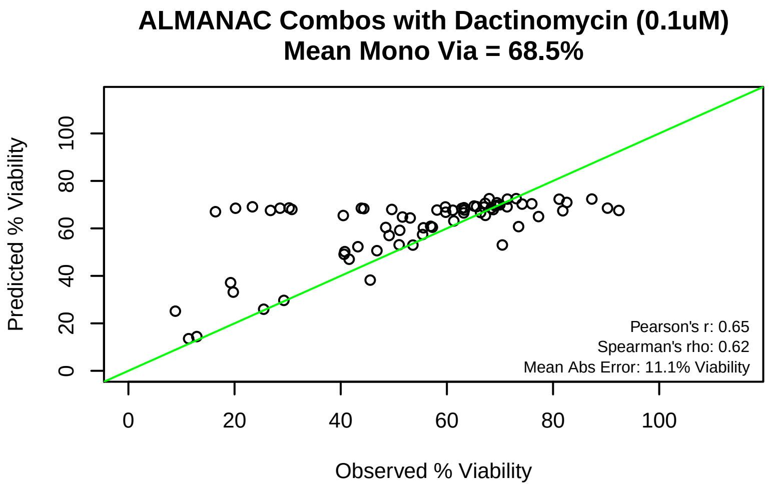
ALMANAC Combos with Dactinomycin (0.1uM)
Mean Mono Via = 68.5%
Pearson's r: 0.65
Spearman's rho: 0.62
Mean Abs Error: 11.1% Viability
Predicted % Viability
Observed % Viability

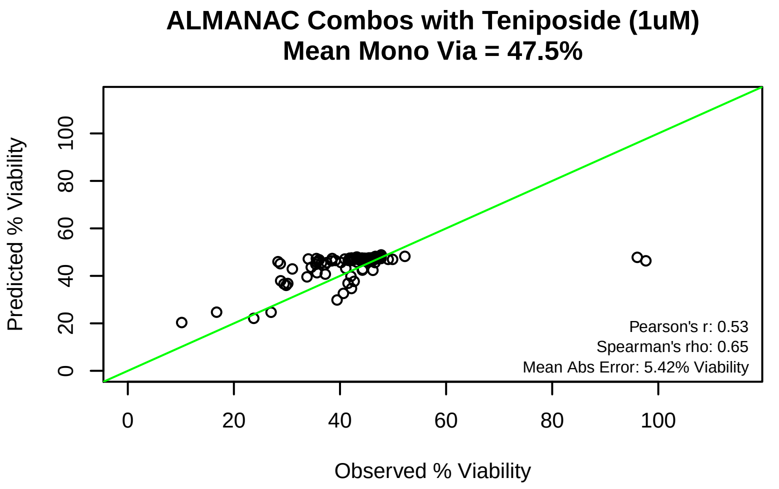
ALMANAC Combos with Teniposide (1uM)
Mean Mono Via = 47.5%
Pearson's r: 0.53
Spearman's rho: 0.65
Mean Abs Error: 5.42% Viability
Predicted % Viability
Observed % Viability

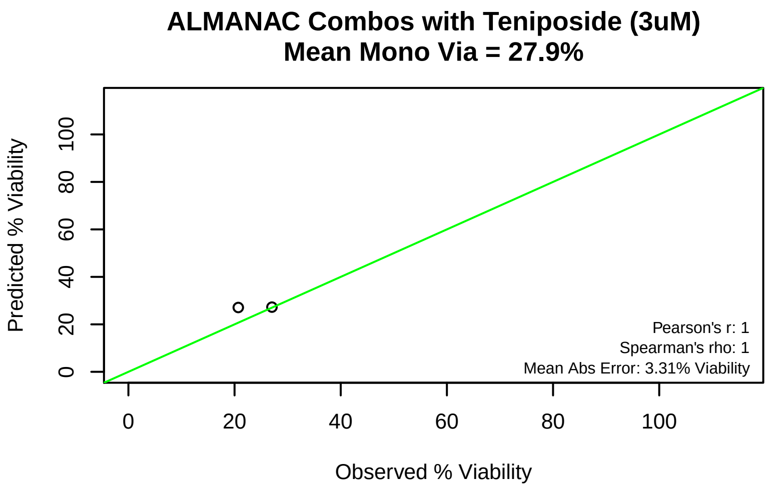
ALMANAC Combos with Teniposide (3uM)
Mean Mono Via = 27.9%
Pearson's r: 1
Spearman's rho: 1
Mean Abs Error: 3.31% Viability
Predicted % Viability
Observed % Viability

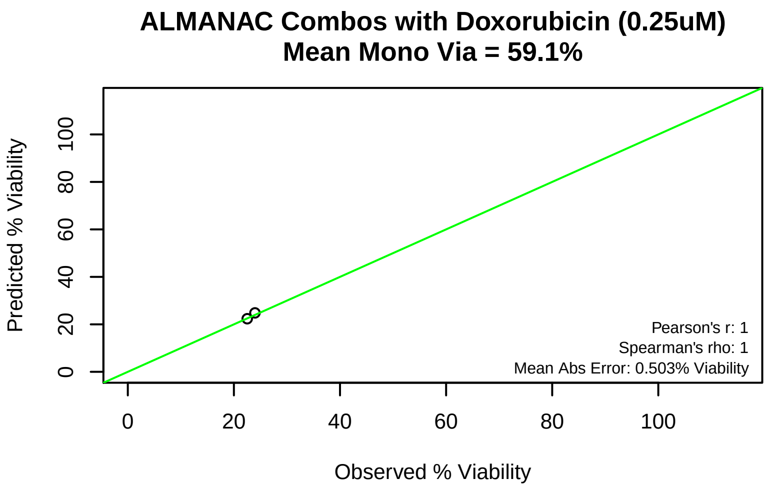
ALMANAC Combos with Doxorubicin (0.25uM)
Mean Mono Via = 59.1%
Pearson's r: 1
Spearman's rho: 1
Mean Abs Error: 0.503% Viability
Predicted % Viability
Observed % Viability

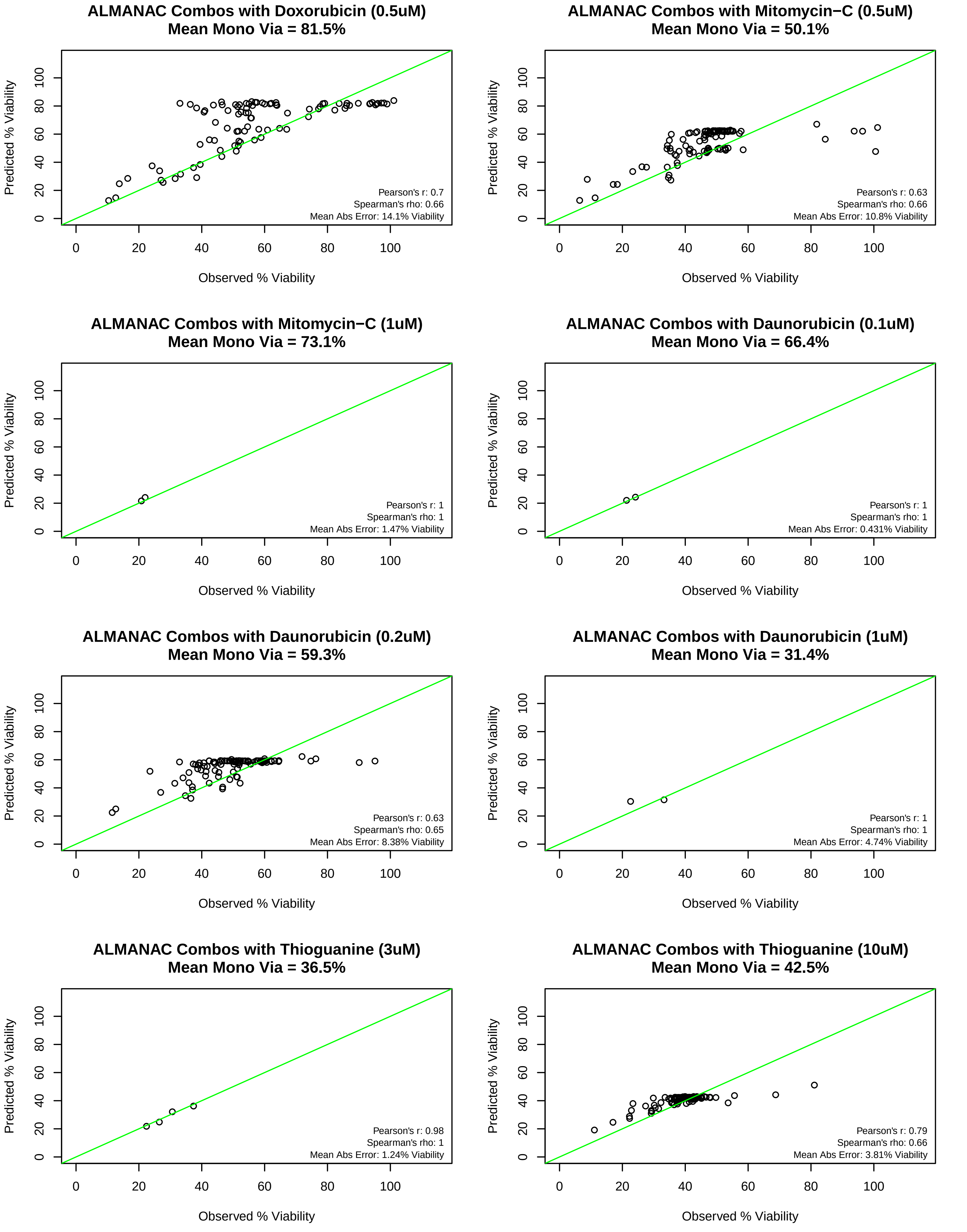
ALMANAC Combos with Doxorubicin (0.5uM)
Mean Mono Via = 81.5%
Pearson's r: 0.7
Spearman's rho: 0.66
Mean Abs Error: 14.1% Viability
ALMANAC Combos with Mitomycin−C (0.5uM)
Mean Mono Via = 50.1%
Pearson's r: 0.63
Spearman's rho: 0.66
Mean Abs Error: 10.8% Viability
ALMANAC Combos with Mitomycin−C (1uM)
Mean Mono Via = 73.1%
Pearson's r: 1
Spearman's rho: 1
Mean Abs Error: 1.47% Viability
ALMANAC Combos with Daunorubicin (0.1uM)
Mean Mono Via = 66.4%
Pearson's r: 1
Spearman's rho: 1
Mean Abs Error: 0.431% Viability
ALMANAC Combos with Daunorubicin (0.2uM)
Mean Mono Via = 59.3%
Pearson's r: 0.63
Spearman's rho: 0.65
Mean Abs Error: 8.38% Viability
ALMANAC Combos with Daunorubicin (1uM)
Mean Mono Via = 31.4%
Pearson's r: 1
Spearman's rho: 1
Mean Abs Error: 4.74% Viability
ALMANAC Combos with Thioguanine (3uM)
Mean Mono Via = 36.5%
Pearson's r: 0.98
Spearman's rho: 1
Mean Abs Error: 1.24% Viability
ALMANAC Combos with Thioguanine (10uM)
Mean Mono Via = 42.5%
Pearson's r: 0.79
Spearman's rho: 0.66
Mean Abs Error: 3.81% Viability
Predicted % Viability
Observed % Viability
0
20
40
60
80
100

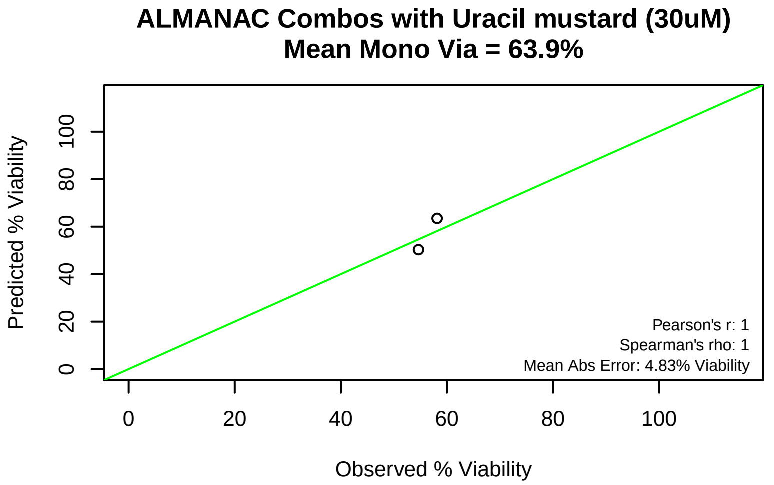
ALMANAC Combos with Uracil mustard (30uM)
Mean Mono Via = 63.9%
Predicted % Viability
Observed % Viability
Pearson's r: 1
Spearman's rho: 1
Mean Abs Error: 4.83% Viability

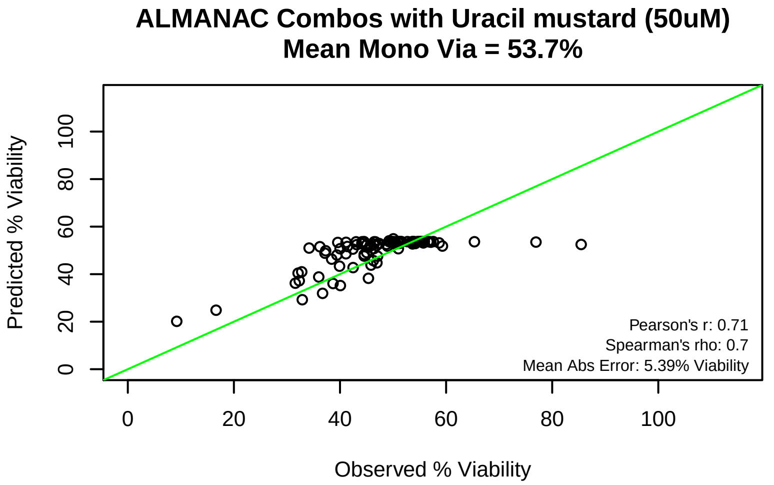
ALMANAC Combos with Uracil mustard (50uM)
Mean Mono Via = 53.7%
Predicted % Viability
Observed % Viability
Pearson's r: 0.71
Spearman's rho: 0.7
Mean Abs Error: 5.39% Viability

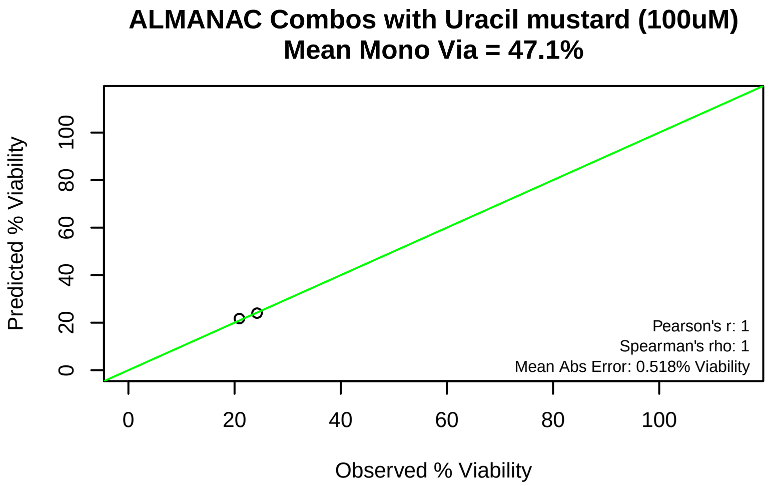
ALMANAC Combos with Uracil mustard (100uM)
Mean Mono Via = 47.1%
Predicted % Viability
Observed % Viability
Pearson's r: 1
Spearman's rho: 1
Mean Abs Error: 0.518% Viability

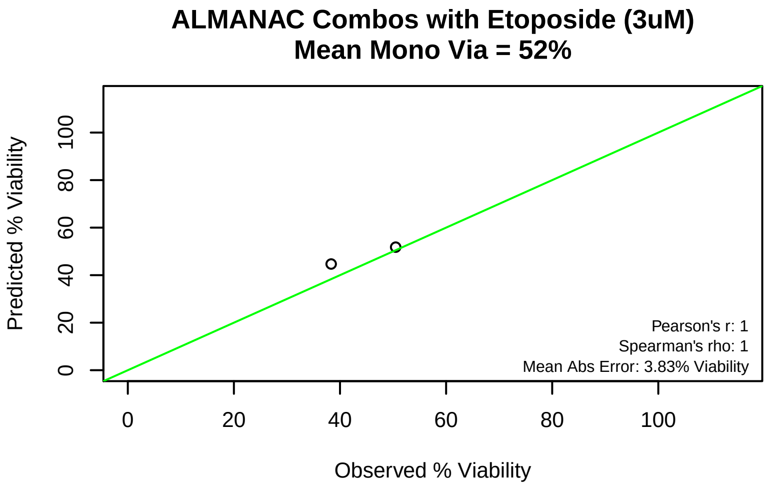
ALMANAC Combos with Etoposide (3uM)
Mean Mono Via = 52%
Predicted % Viability
Observed % Viability
Pearson's r: 1
Spearman's rho: 1
Mean Abs Error: 3.83% Viability

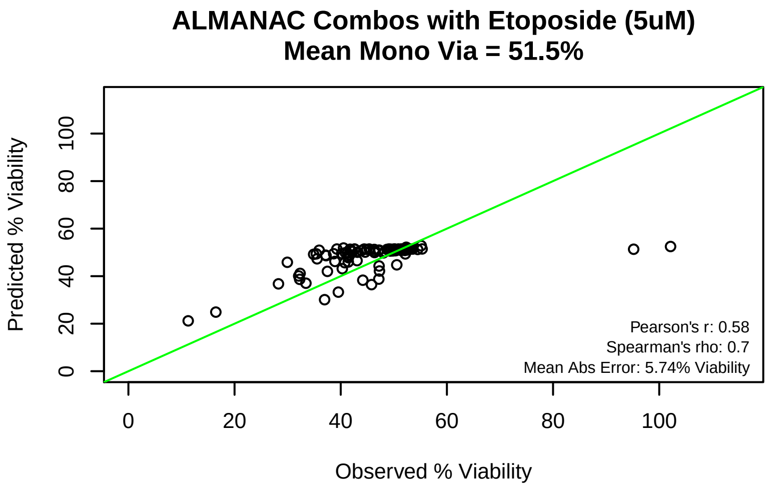
ALMANAC Combos with Etoposide (5uM)
Mean Mono Via = 51.5%
Predicted % Viability
Observed % Viability
Pearson's r: 0.58
Spearman's rho: 0.7
Mean Abs Error: 5.74% Viability

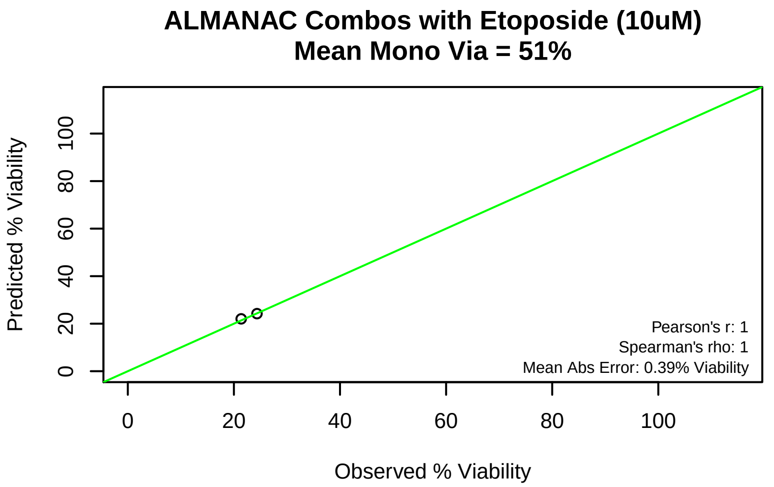
ALMANAC Combos with Etoposide (10uM)
Mean Mono Via = 51%
Predicted % Viability
Observed % Viability
Pearson's r: 1
Spearman's rho: 1
Mean Abs Error: 0.39% Viability

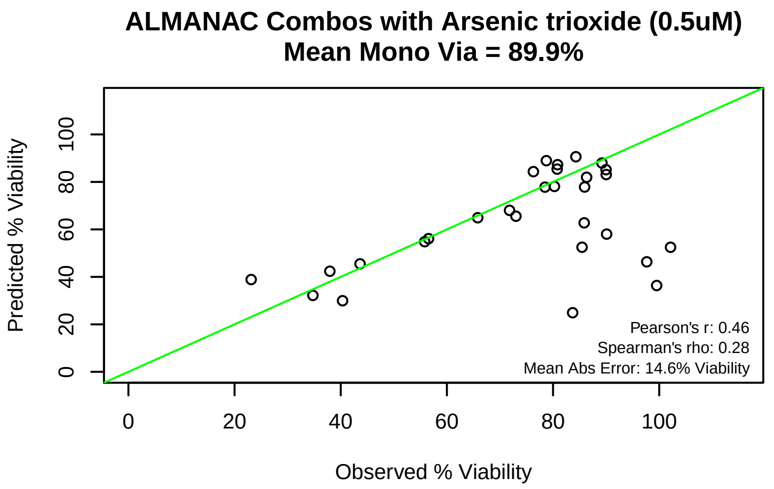
ALMANAC Combos with Arsenic trioxide (0.5uM)
Mean Mono Via = 89.9%
Predicted % Viability
Observed % Viability
Pearson's r: 0.46
Spearman's rho: 0.28
Mean Abs Error: 14.6% Viability

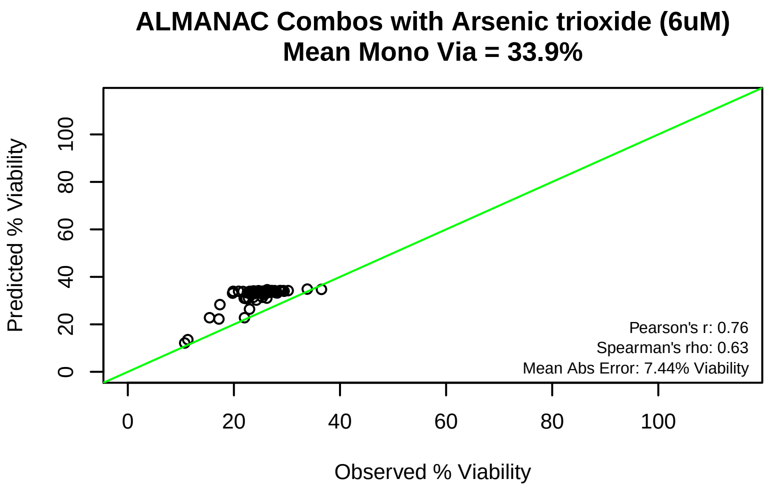
ALMANAC Combos with Arsenic trioxide (6uM)
Mean Mono Via = 33.9%
Predicted % Viability
Observed % Viability
Pearson's r: 0.76
Spearman's rho: 0.63
Mean Abs Error: 7.44% Viability

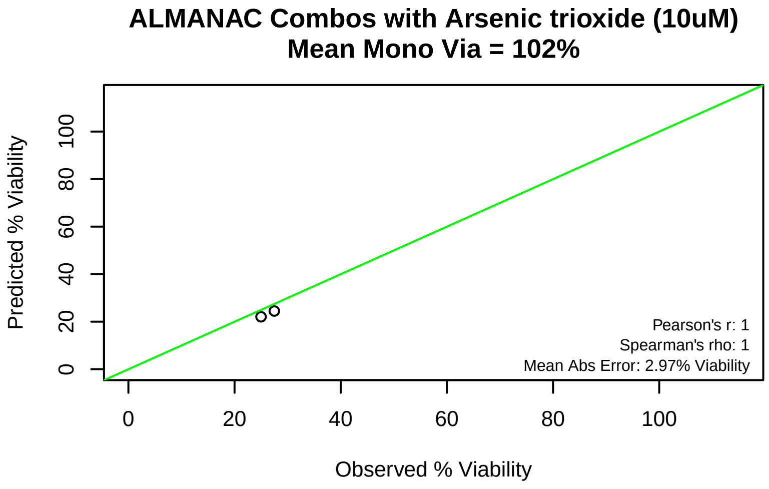
ALMANAC Combos with Arsenic trioxide (10uM)
Mean Mono Via = 102%
Predicted % Viability
Observed % Viability
Pearson's r: 1
Spearman's rho: 1
Mean Abs Error: 2.97% Viability

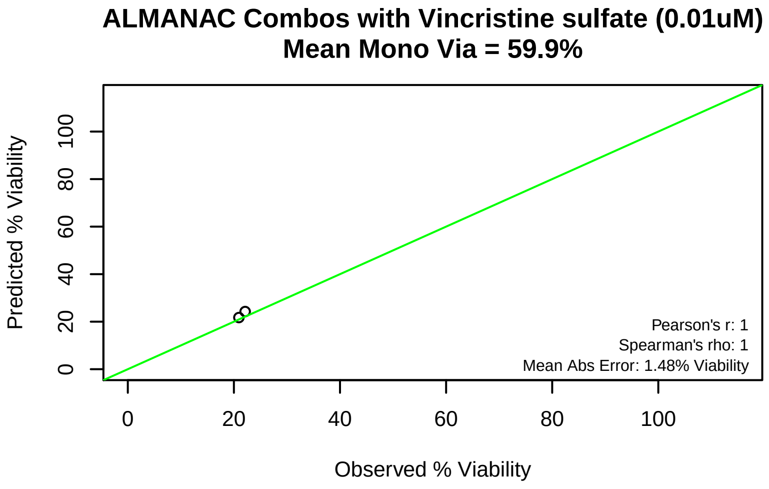
ALMANAC Combos with Vincristine sulfate (0.01uM)
Mean Mono Via = 59.9%
Predicted % Viability
Observed % Viability
Pearson's r: 1
Spearman's rho: 1
Mean Abs Error: 1.48% Viability

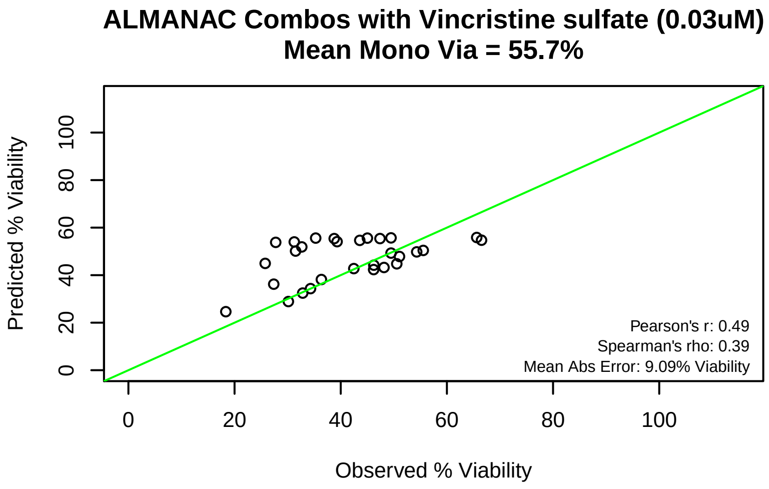
ALMANAC Combos with Vincristine sulfate (0.03uM)
Mean Mono Via = 55.7%
Predicted % Viability
Observed % Viability
Pearson's r: 0.49
Spearman's rho: 0.39
Mean Abs Error: 9.09% Viability

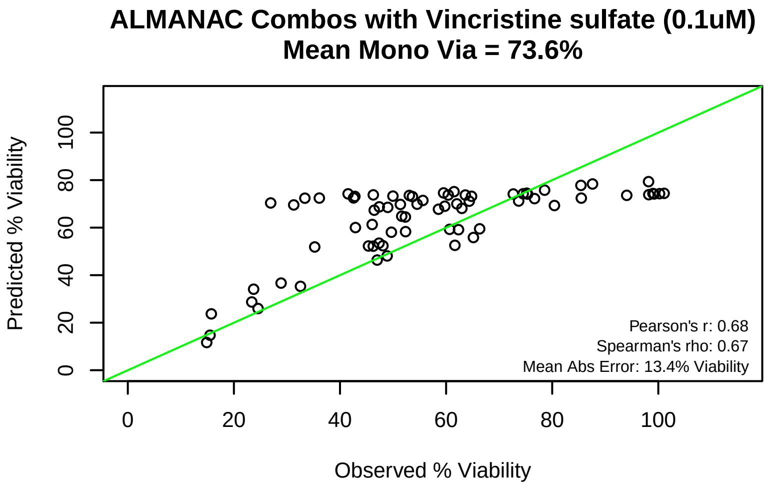
ALMANAC Combos with Vincristine sulfate (0.1uM)
Mean Mono Via = 73.6%
Predicted % Viability
Observed % Viability
Pearson's r: 0.68
Spearman's rho: 0.67
Mean Abs Error: 13.4% Viability

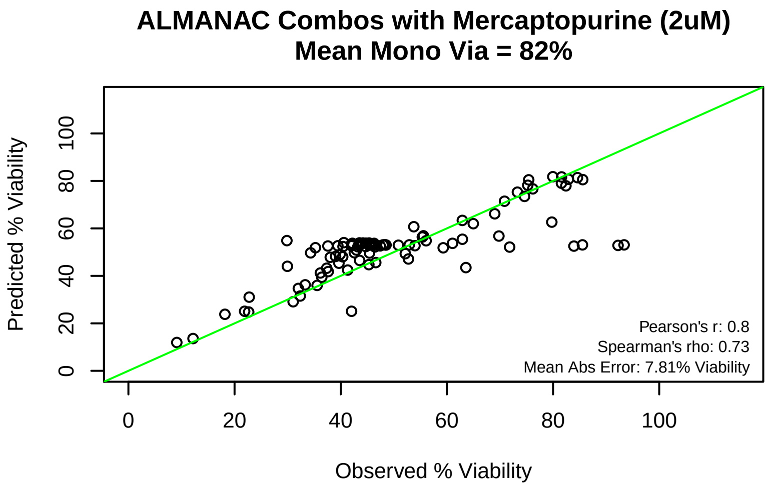
ALMANAC Combos with Mercaptopurine (2uM)
Mean Mono Via = 82%
Predicted % Viability
Observed % Viability
Pearson's r: 0.8
Spearman's rho: 0.73
Mean Abs Error: 7.81% Viability

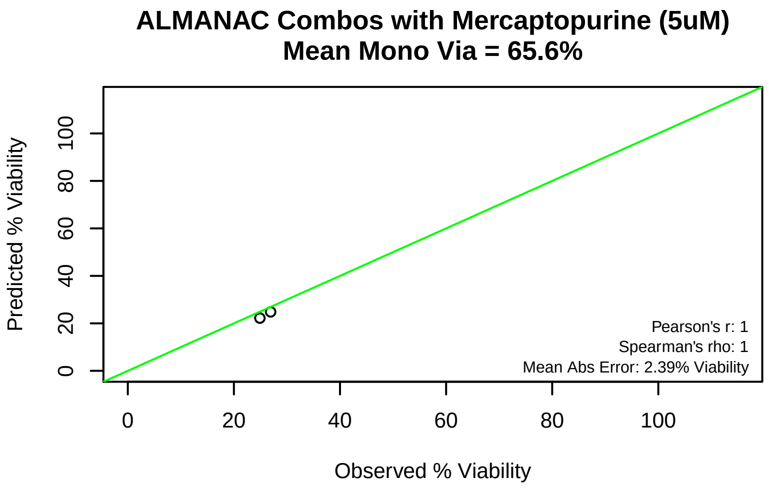
ALMANAC Combos with Mercaptopurine (5uM)
Mean Mono Via = 65.6%
Predicted % Viability
Observed % Viability
Pearson's r: 1
Spearman's rho: 1
Mean Abs Error: 2.39% Viability

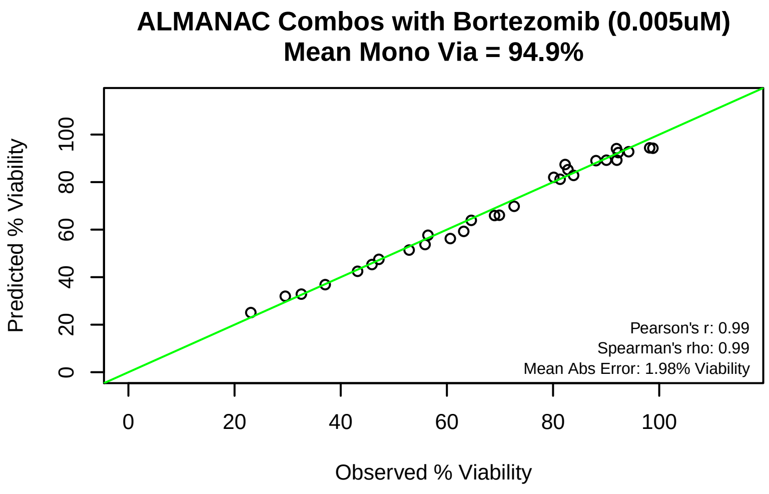
ALMANAC Combos with Bortezomib (0.005uM)
Mean Mono Via = 94.9%
Predicted % Viability
Observed % Viability
Pearson's r: 0.99
Spearman's rho: 0.99
Mean Abs Error: 1.98% Viability

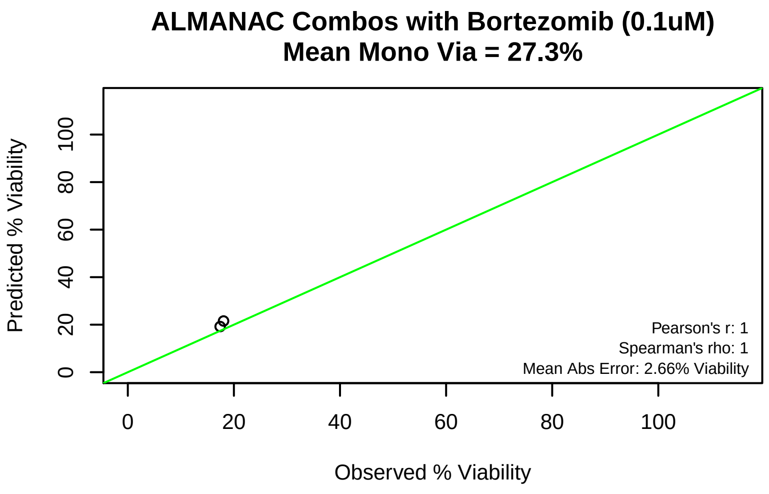
ALMANAC Combos with Bortezomib (0.1uM)
Mean Mono Via = 27.3%
Predicted % Viability
Observed % Viability
Pearson's r: 1
Spearman's rho: 1
Mean Abs Error: 2.66% Viability

ALMANAC Combos with Bortezomib (0.3uM)
Mean Mono Via = 12.8%

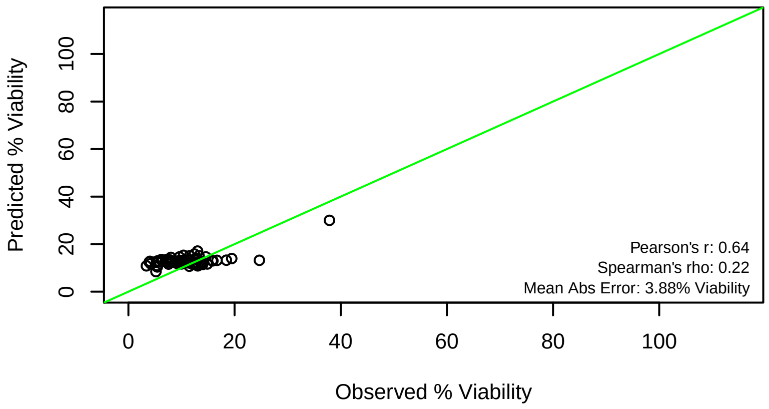

ALMANAC Combos with Bortezomib (1uM)
Mean Mono Via = 21.5%

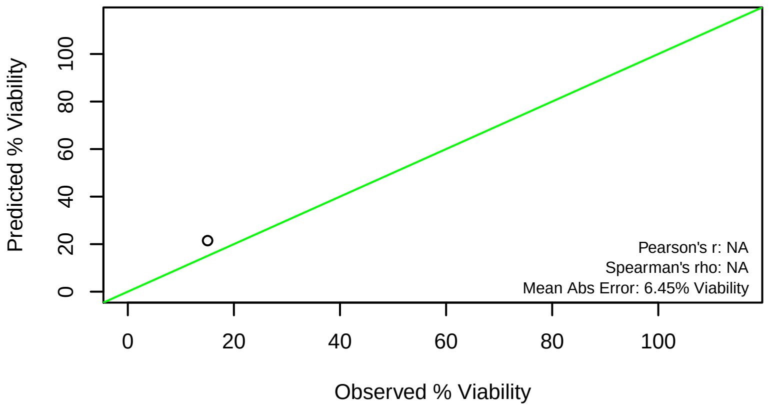

ALMANAC Combos with Docetaxel (0.01uM)
Mean Mono Via = 62.5%

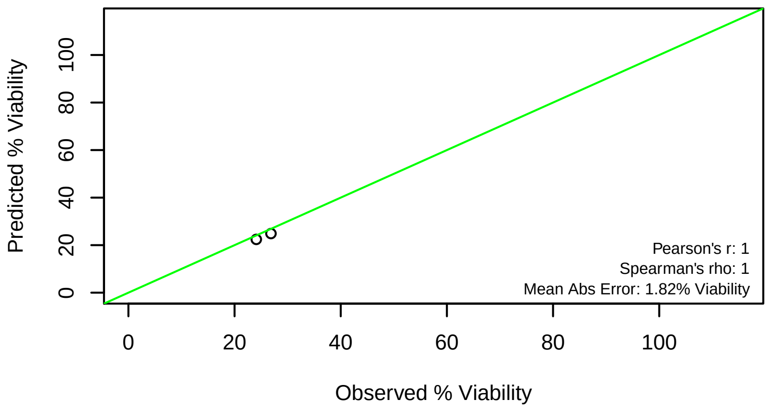

ALMANAC Combos with Docetaxel (0.03uM)
Mean Mono Via = 42.2%

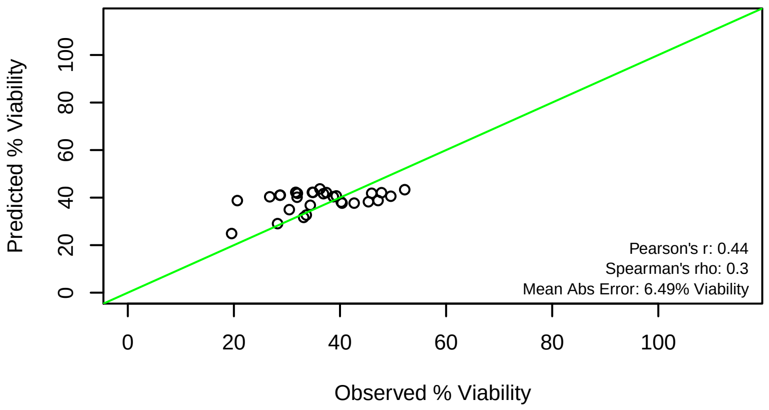

ALMANAC Combos with Docetaxel (0.04uM)
Mean Mono Via = 77.1%

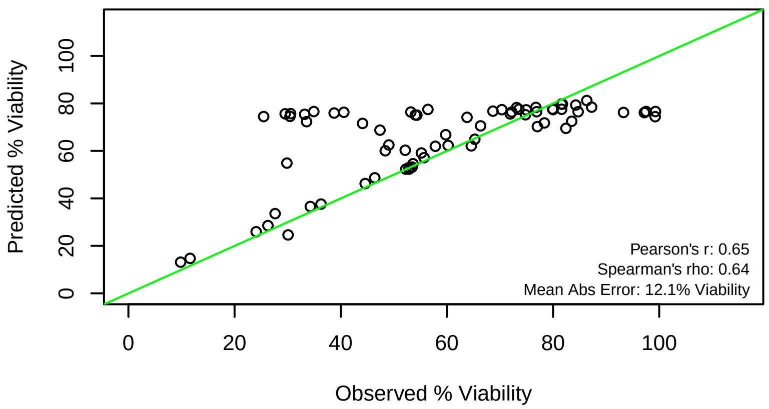

ALMANAC Combos with Lomustine (10uM)
Mean Mono Via = 89.8%

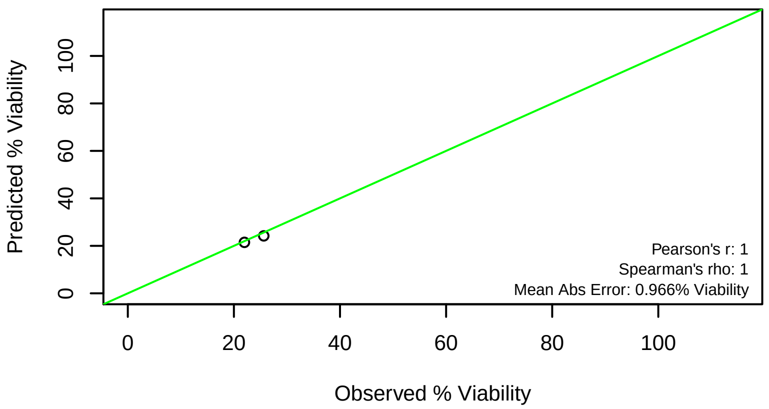

ALMANAC Combos with Lomustine (30uM)
Mean Mono Via = 77.1%

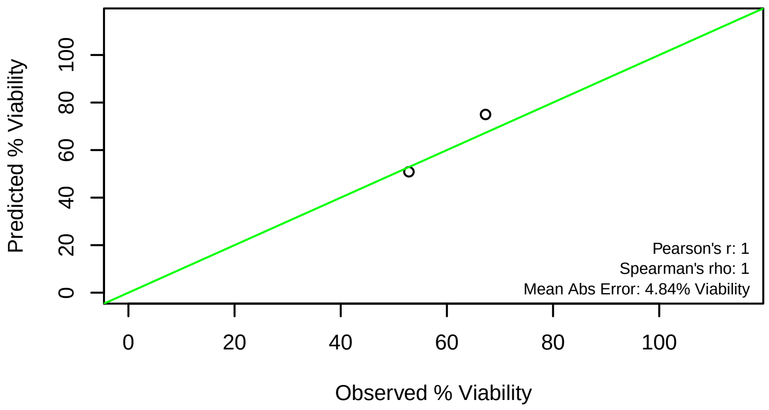

ALMANAC Combos with Lomustine (50uM)
Mean Mono Via = 63.9%

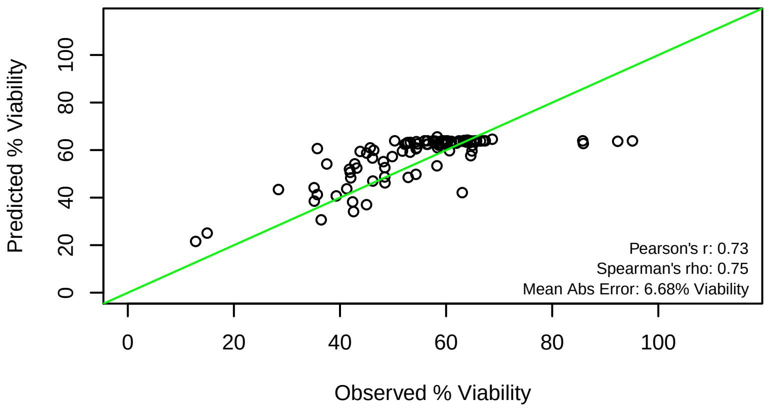

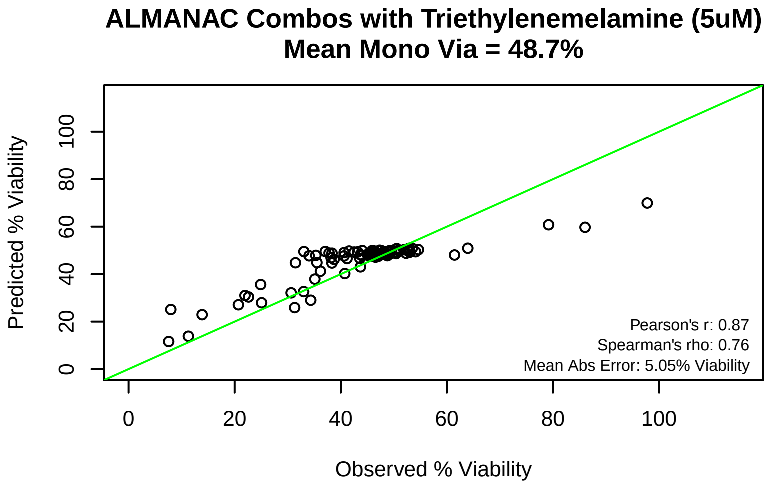
ALMANAC Combos with Triethylenemelamine (5uM)
Mean Mono Via = 48.7%
Predicted % Viability
Observed % Viability
Pearson's r: 0.87
Spearman's rho: 0.76
Mean Abs Error: 5.05% Viability

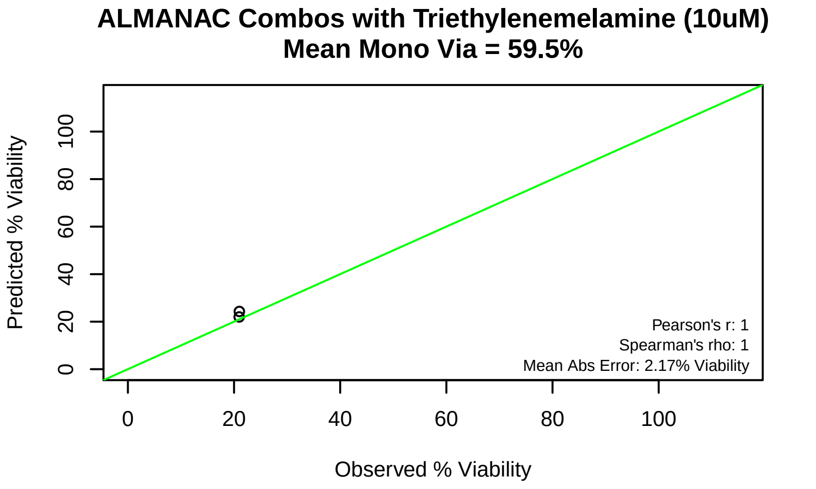
ALMANAC Combos with Triethylenemelamine (10uM)
Mean Mono Via = 59.5%
Predicted % Viability
Observed % Viability
Pearson's r: 1
Spearman's rho: 1
Mean Abs Error: 2.17% Viability

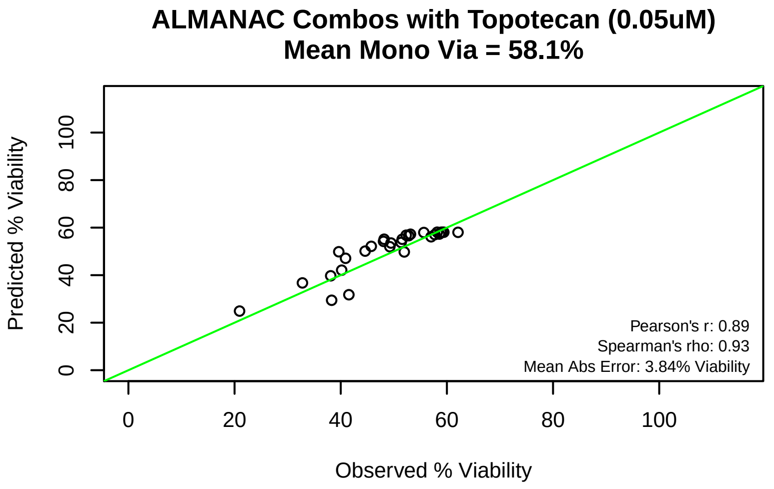
ALMANAC Combos with Topotecan (0.05uM)
Mean Mono Via = 58.1%
Predicted % Viability
Observed % Viability
Pearson's r: 0.89
Spearman's rho: 0.93
Mean Abs Error: 3.84% Viability

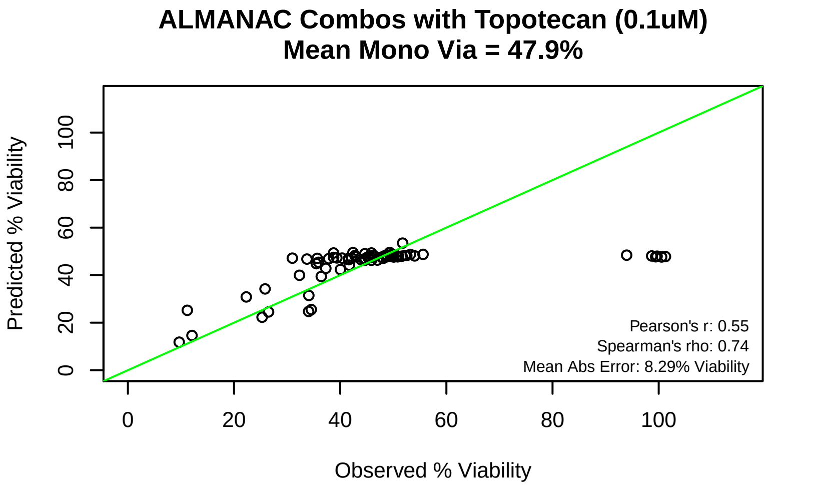
ALMANAC Combos with Topotecan (0.1uM)
Mean Mono Via = 47.9%
Predicted % Viability
Observed % Viability
Pearson's r: 0.55
Spearman's rho: 0.74
Mean Abs Error: 8.29% Viability

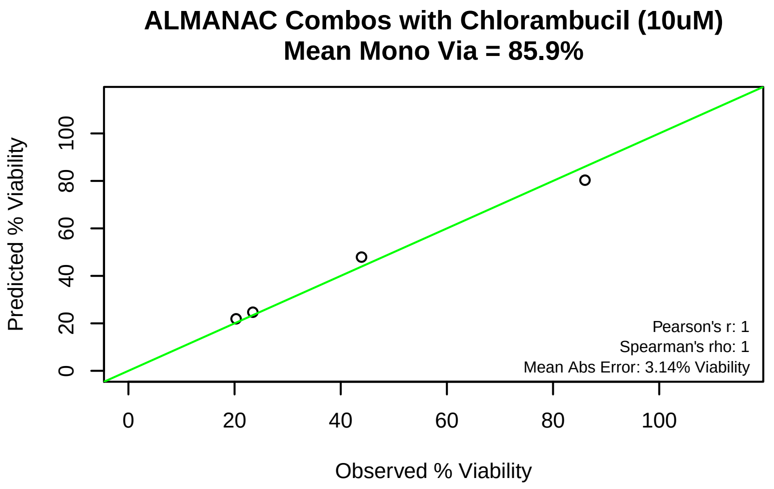
ALMANAC Combos with Chlorambucil (10uM)
Mean Mono Via = 85.9%
Predicted % Viability
Observed % Viability
Pearson's r: 1
Spearman's rho: 1
Mean Abs Error: 3.14% Viability

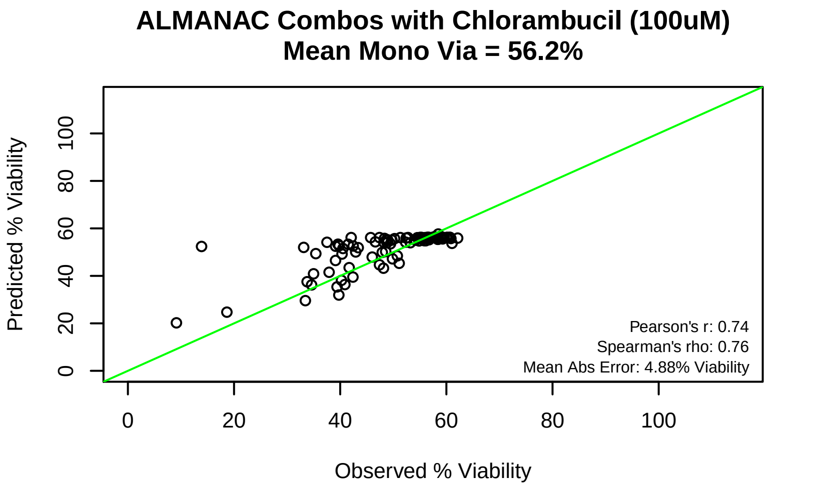
ALMANAC Combos with Chlorambucil (100uM)
Mean Mono Via = 56.2%
Predicted % Viability
Observed % Viability
Pearson's r: 0.74
Spearman's rho: 0.76
Mean Abs Error: 4.88% Viability

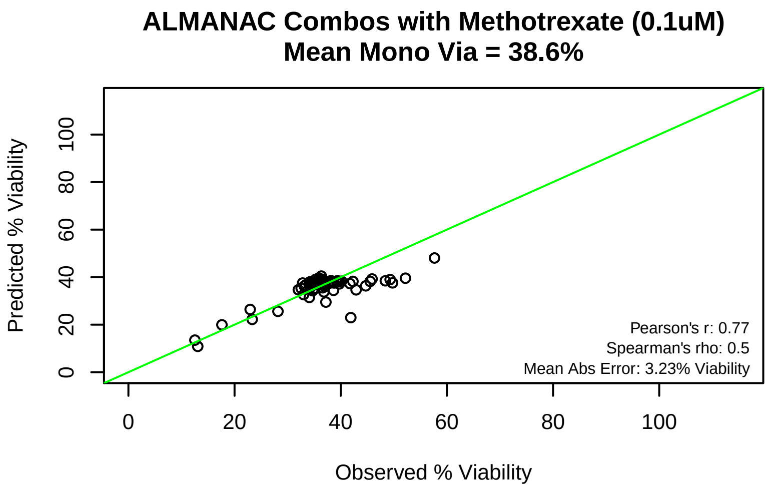
ALMANAC Combos with Methotrexate (0.1uM)
Mean Mono Via = 38.6%
Predicted % Viability
Observed % Viability
Pearson's r: 0.77
Spearman's rho: 0.5
Mean Abs Error: 3.23% Viability

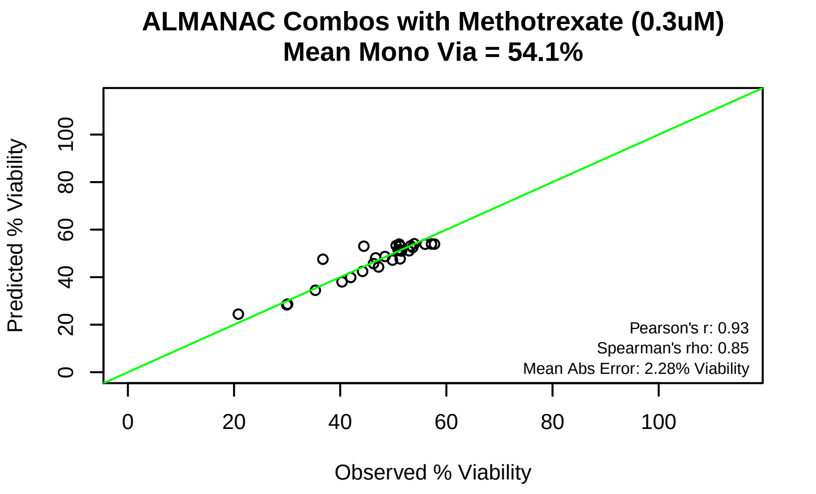
ALMANAC Combos with Methotrexate (0.3uM)
Mean Mono Via = 54.1%
Predicted % Viability
Observed % Viability
Pearson's r: 0.93
Spearman's rho: 0.85
Mean Abs Error: 2.28% Viability

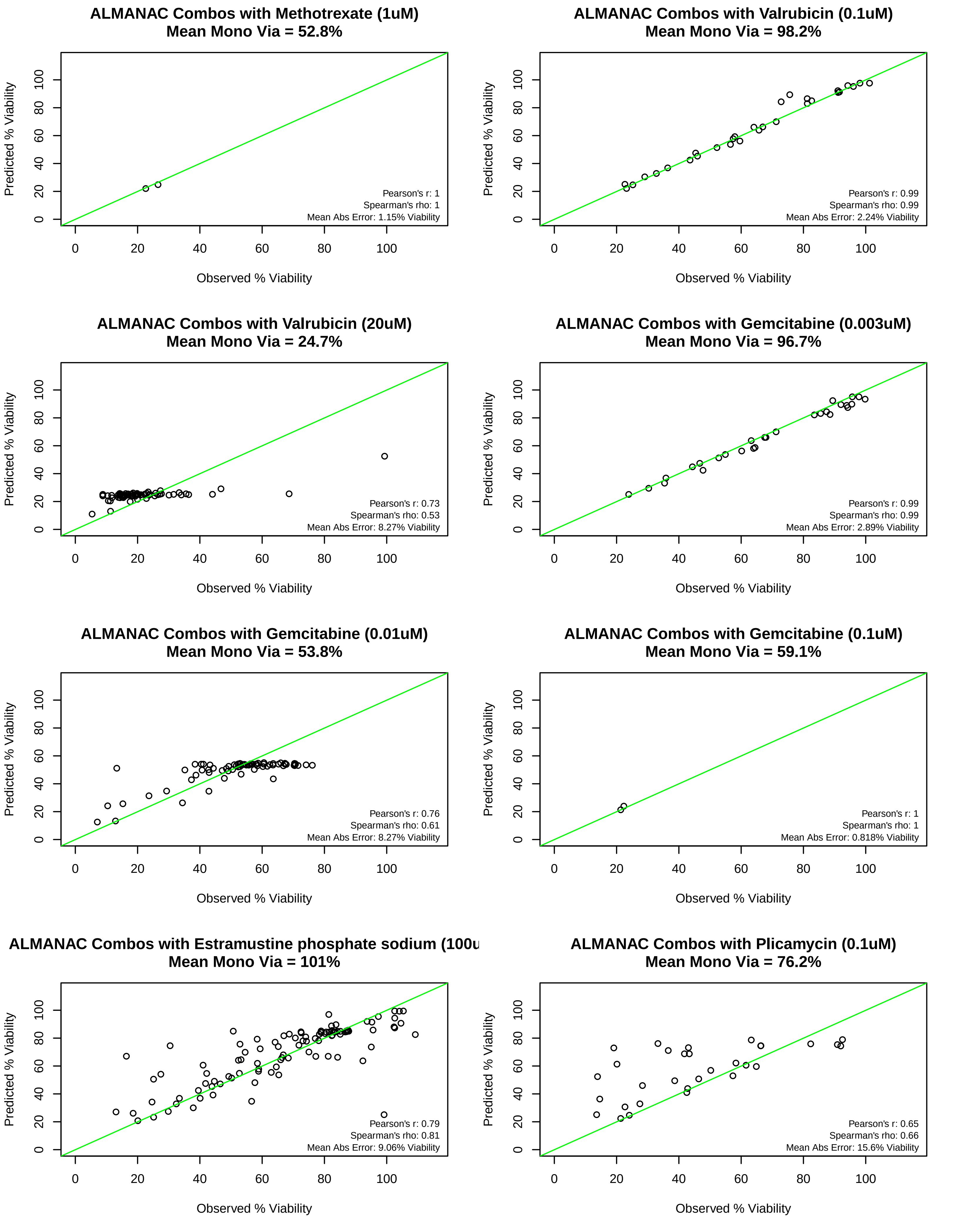
ALMANAC Combos with Methotrexate (1uM)
Mean Mono Via = 52.8%
Pearson's r: 1
Spearman's rho: 1
Mean Abs Error: 1.15% Viability
Predicted % Viability
Observed % Viability
ALMANAC Combos with Valrubicin (0.1uM)
Mean Mono Via = 98.2%
Pearson's r: 0.99
Spearman's rho: 0.99
Mean Abs Error: 2.24% Viability
Predicted % Viability
Observed % Viability
ALMANAC Combos with Valrubicin (20uM)
Mean Mono Via = 24.7%
Pearson's r: 0.73
Spearman's rho: 0.53
Mean Abs Error: 8.27% Viability
Predicted % Viability
Observed % Viability
ALMANAC Combos with Gemcitabine (0.003uM)
Mean Mono Via = 96.7%
Pearson's r: 0.99
Spearman's rho: 0.99
Mean Abs Error: 2.89% Viability
Predicted % Viability
Observed % Viability
ALMANAC Combos with Gemcitabine (0.01uM)
Mean Mono Via = 53.8%
Pearson's r: 0.76
Spearman's rho: 0.61
Mean Abs Error: 8.27% Viability
Predicted % Viability
Observed % Viability
ALMANAC Combos with Gemcitabine (0.1uM)
Mean Mono Via = 59.1%
Pearson's r: 1
Spearman's rho: 1
Mean Abs Error: 0.818% Viability
Predicted % Viability
Observed % Viability
ALMANAC Combos with Estramustine phosphate sodium (100u
Mean Mono Via = 101%
Pearson's r: 0.79
Spearman's rho: 0.81
Mean Abs Error: 9.06% Viability
Predicted % Viability
Observed % Viability
ALMANAC Combos with Plicamycin (0.1uM)
Mean Mono Via = 76.2%
Pearson's r: 0.65
Spearman's rho: 0.66
Mean Abs Error: 15.6% Viability
Predicted % Viability
Observed % Viability

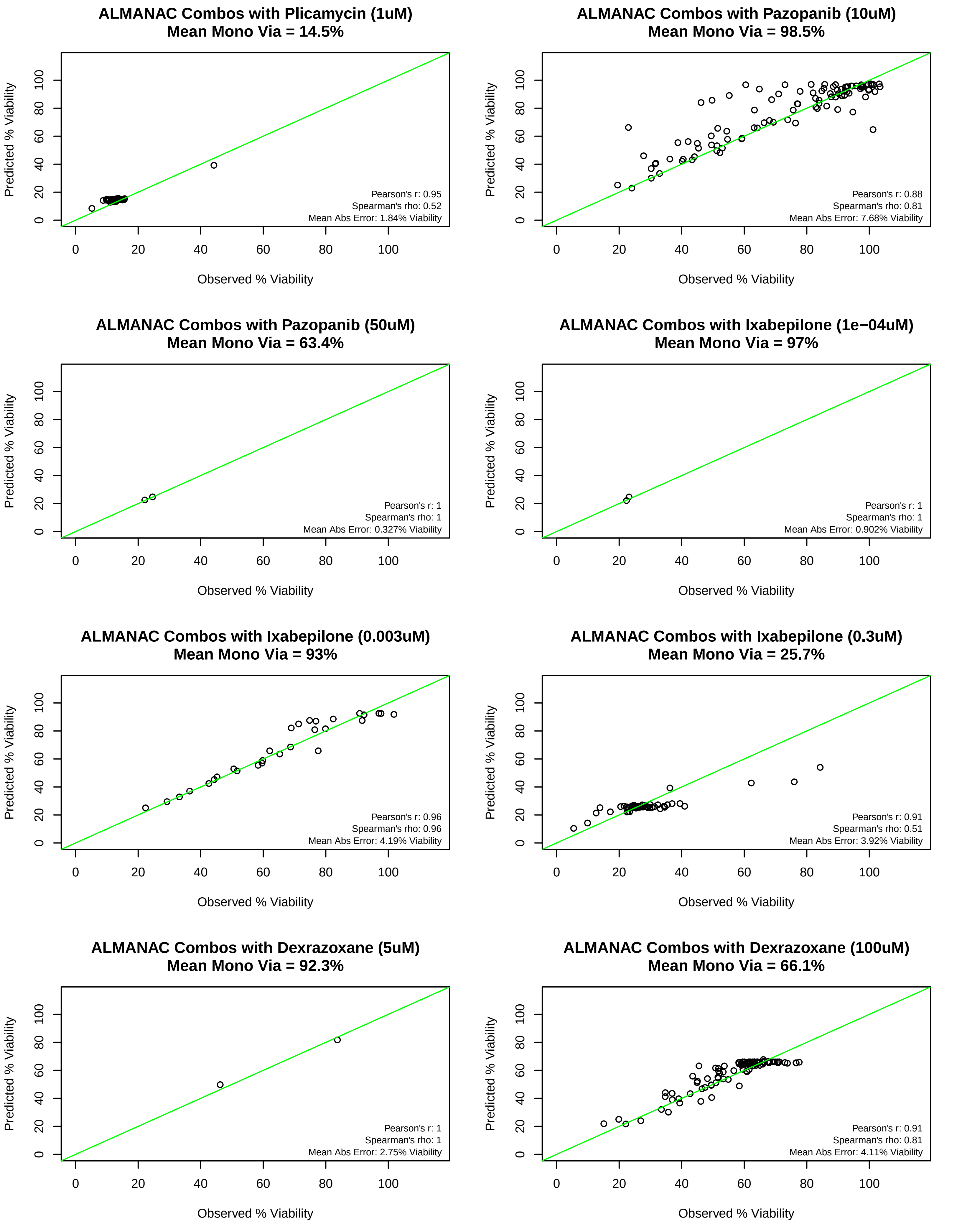
ALMANAC Combos with Plicamycin (1uM)
Mean Mono Via = 14.5%
Predicted % Viability
Observed % Viability
Pearson's r: 0.95
Spearman's rho: 0.52
Mean Abs Error: 1.84% Viability
ALMANAC Combos with Pazopanib (10uM)
Mean Mono Via = 98.5%
Predicted % Viability
Observed % Viability
Pearson's r: 0.88
Spearman's rho: 0.81
Mean Abs Error: 7.68% Viability
ALMANAC Combos with Pazopanib (50uM)
Mean Mono Via = 63.4%
Predicted % Viability
Observed % Viability
Pearson's r: 1
Spearman's rho: 1
Mean Abs Error: 0.327% Viability
ALMANAC Combos with Ixabepilone (1e−04uM)
Mean Mono Via = 97%
Predicted % Viability
Observed % Viability
Pearson's r: 1
Spearman's rho: 1
Mean Abs Error: 0.902% Viability
ALMANAC Combos with Ixabepilone (0.003uM)
Mean Mono Via = 93%
Predicted % Viability
Observed % Viability
Pearson's r: 0.96
Spearman's rho: 0.96
Mean Abs Error: 4.19% Viability
ALMANAC Combos with Ixabepilone (0.3uM)
Mean Mono Via = 25.7%
Predicted % Viability
Observed % Viability
Pearson's r: 0.91
Spearman's rho: 0.51
Mean Abs Error: 3.92% Viability
ALMANAC Combos with Dexrazoxane (5uM)
Mean Mono Via = 92.3%
Predicted % Viability
Observed % Viability
Pearson's r: 1
Spearman's rho: 1
Mean Abs Error: 2.75% Viability
ALMANAC Combos with Dexrazoxane (100uM)
Mean Mono Via = 66.1%
Predicted % Viability
Observed % Viability
Pearson's r: 0.91
Spearman's rho: 0.81
Mean Abs Error: 4.11% Viability

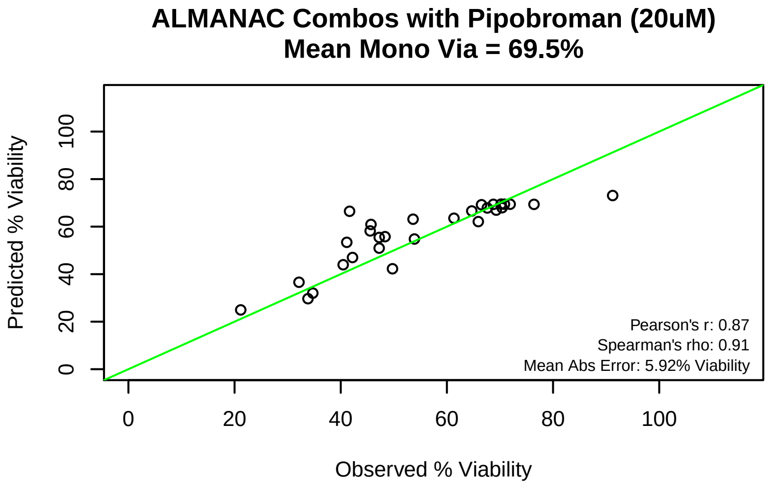
ALMANAC Combos with Pipobroman (20uM)
Mean Mono Via = 69.5%
Predicted % Viability
Observed % Viability
Pearson's r: 0.87
Spearman's rho: 0.91
Mean Abs Error: 5.92% Viability

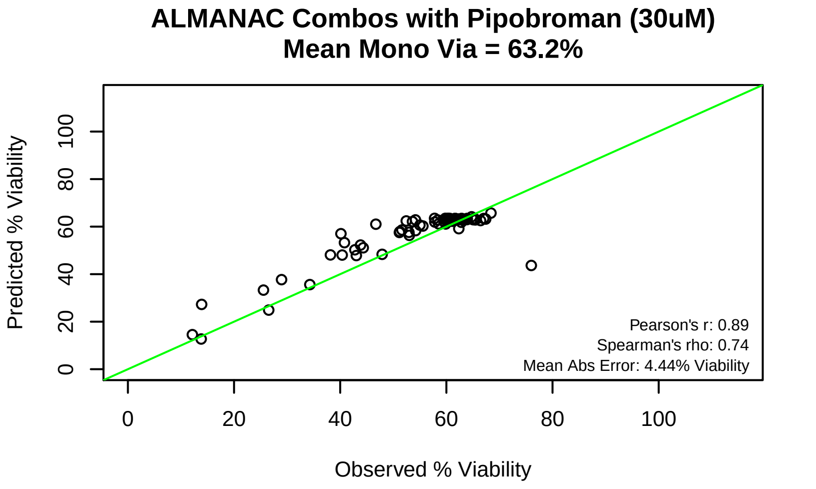
ALMANAC Combos with Pipobroman (30uM)
Mean Mono Via = 63.2%
Predicted % Viability
Observed % Viability
Pearson's r: 0.89
Spearman's rho: 0.74
Mean Abs Error: 4.44% Viability

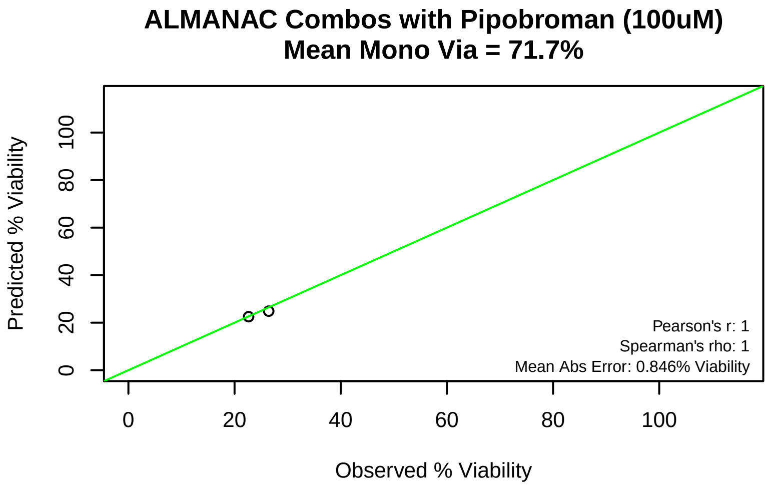
ALMANAC Combos with Pipobroman (100uM)
Mean Mono Via = 71.7%
Predicted % Viability
Observed % Viability
Pearson's r: 1
Spearman's rho: 1
Mean Abs Error: 0.846% Viability

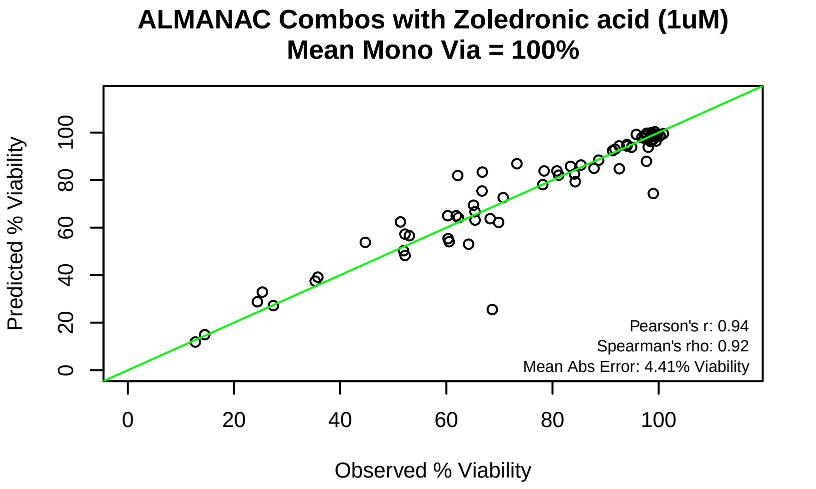
ALMANAC Combos with Zoledronic acid (1uM)
Mean Mono Via = 100%
Predicted % Viability
Observed % Viability
Pearson's r: 0.94
Spearman's rho: 0.92
Mean Abs Error: 4.41% Viability

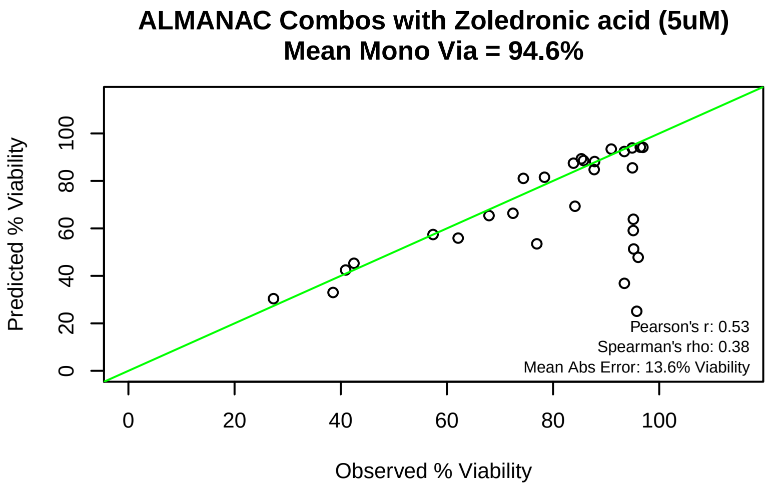
ALMANAC Combos with Zoledronic acid (5uM)
Mean Mono Via = 94.6%
Predicted % Viability
Observed % Viability
Pearson's r: 0.53
Spearman's rho: 0.38
Mean Abs Error: 13.6% Viability

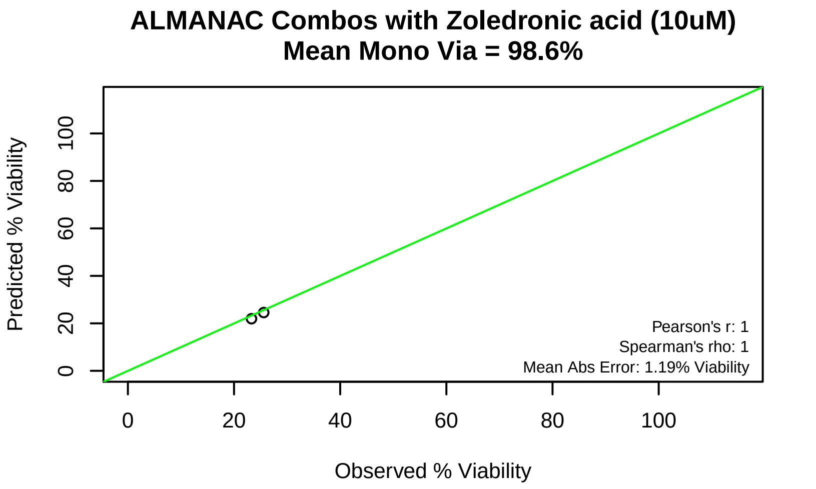
ALMANAC Combos with Zoledronic acid (10uM)
Mean Mono Via = 98.6%
Predicted % Viability
Observed % Viability
Pearson's r: 1
Spearman's rho: 1
Mean Abs Error: 1.19% Viability

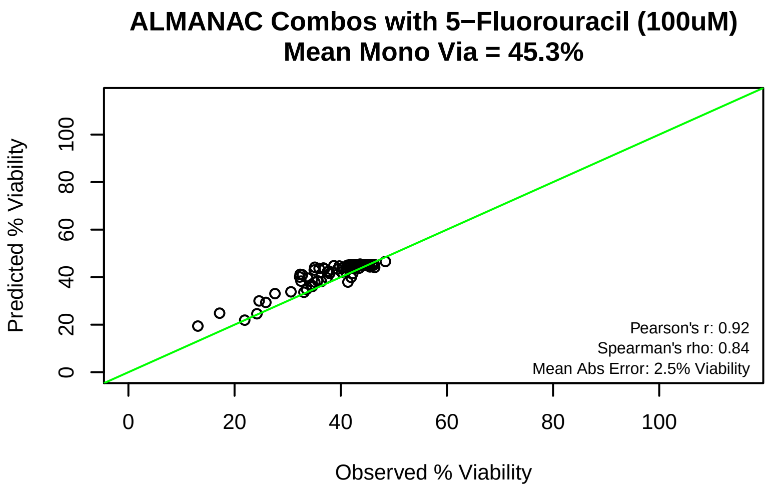
ALMANAC Combos with 5−Fluorouracil (100uM)
Mean Mono Via = 45.3%
Predicted % Viability
Observed % Viability
Pearson's r: 0.92
Spearman's rho: 0.84
Mean Abs Error: 2.5% Viability

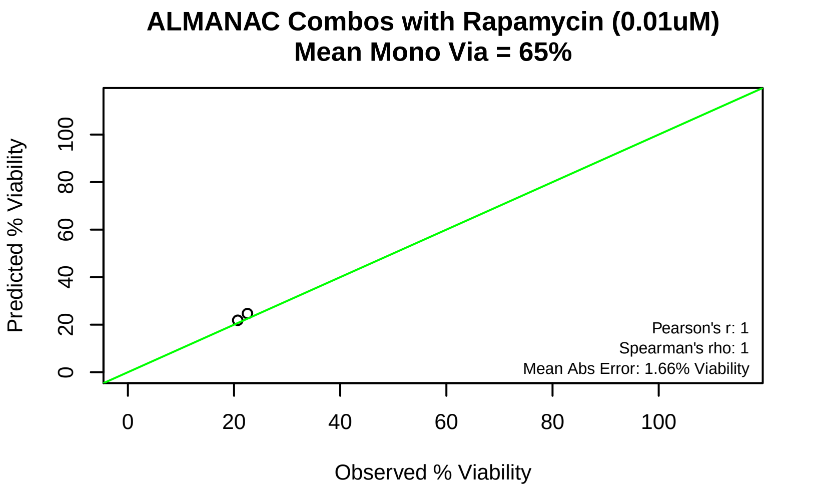
ALMANAC Combos with Rapamycin (0.01uM)
Mean Mono Via = 65%
Predicted % Viability
Observed % Viability
Pearson's r: 1
Spearman's rho: 1
Mean Abs Error: 1.66% Viability

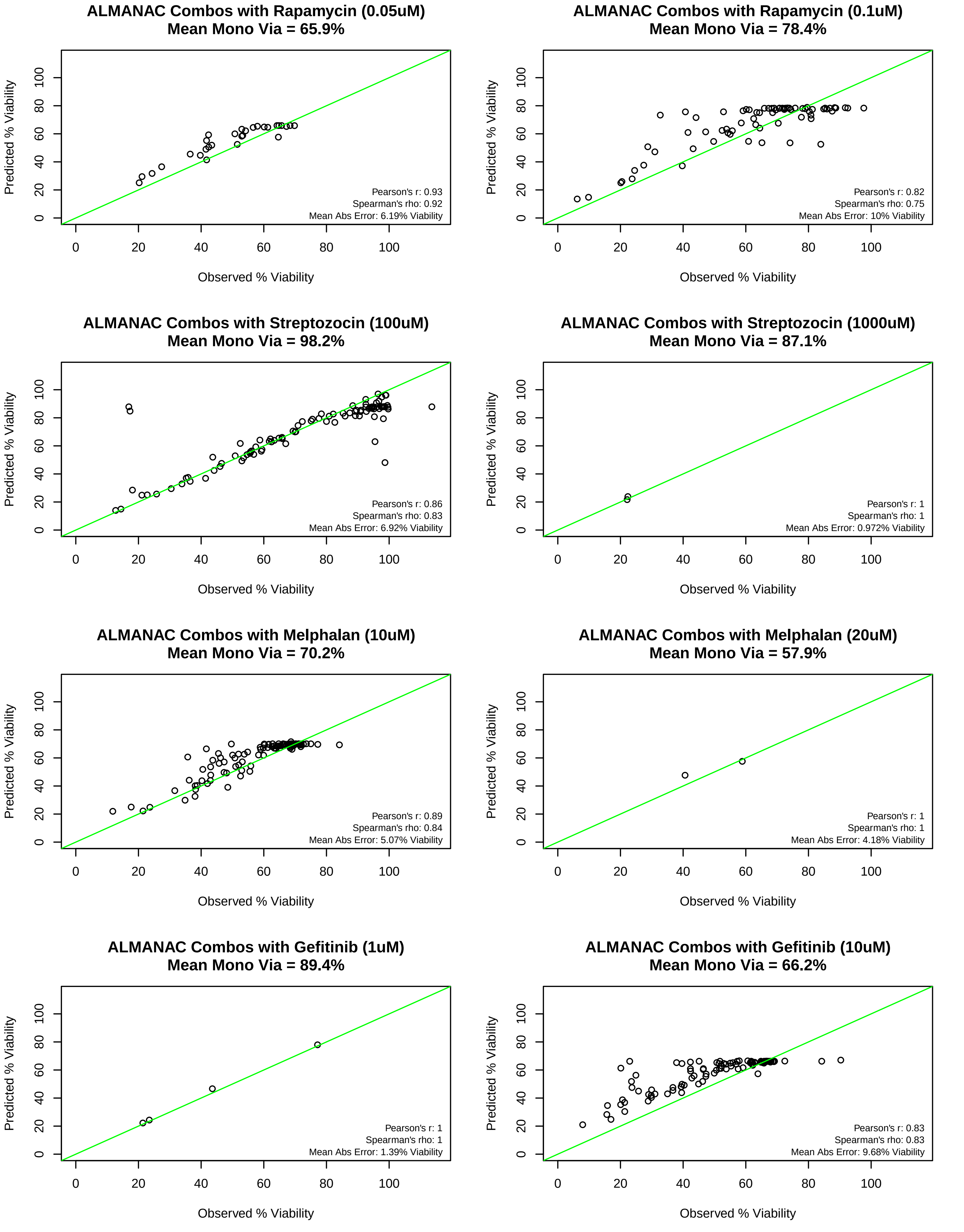
ALMANAC Combos with Rapamycin (0.05uM)
Mean Mono Via = 65.9%
Predicted % Viability
Observed % Viability
Pearson's r: 0.93
Spearman's rho: 0.92
Mean Abs Error: 6.19% Viability
ALMANAC Combos with Rapamycin (0.1uM)
Mean Mono Via = 78.4%
Predicted % Viability
Observed % Viability
Pearson's r: 0.82
Spearman's rho: 0.75
Mean Abs Error: 10% Viability
ALMANAC Combos with Streptozocin (100uM)
Mean Mono Via = 98.2%
Predicted % Viability
Observed % Viability
Pearson's r: 0.86
Spearman's rho: 0.83
Mean Abs Error: 6.92% Viability
ALMANAC Combos with Streptozocin (1000uM)
Mean Mono Via = 87.1%
Predicted % Viability
Observed % Viability
Pearson's r: 1
Spearman's rho: 1
Mean Abs Error: 0.972% Viability
ALMANAC Combos with Melphalan (10uM)
Mean Mono Via = 70.2%
Predicted % Viability
Observed % Viability
Pearson's r: 0.89
Spearman's rho: 0.84
Mean Abs Error: 5.07% Viability
ALMANAC Combos with Melphalan (20uM)
Mean Mono Via = 57.9%
Predicted % Viability
Observed % Viability
Pearson's r: 1
Spearman's rho: 1
Mean Abs Error: 4.18% Viability
ALMANAC Combos with Gefitinib (1uM)
Mean Mono Via = 89.4%
Predicted % Viability
Observed % Viability
Pearson's r: 1
Spearman's rho: 1
Mean Abs Error: 1.39% Viability
ALMANAC Combos with Gefitinib (10uM)
Mean Mono Via = 66.2%
Predicted % Viability
Observed % Viability
Pearson's r: 0.83
Spearman's rho: 0.83
Mean Abs Error: 9.68% Viability

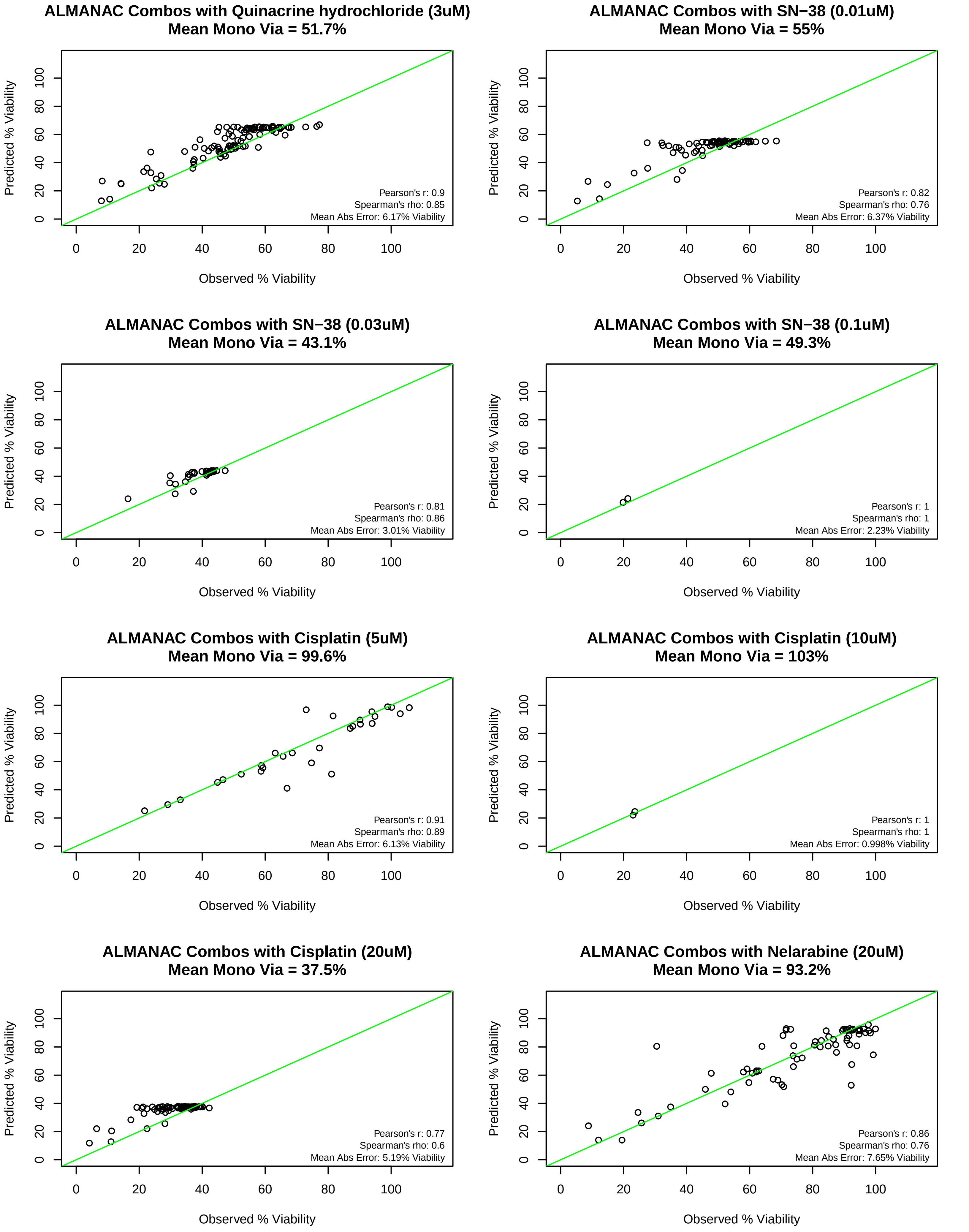
ALMANAC Combos with Quinacrine hydrochloride (3uM)
Mean Mono Via = 51.7%
Pearson's r: 0.9
Spearman's rho: 0.85
Mean Abs Error: 6.17% Viability
ALMANAC Combos with SN−38 (0.01uM)
Mean Mono Via = 55%
Pearson's r: 0.82
Spearman's rho: 0.76
Mean Abs Error: 6.37% Viability
ALMANAC Combos with SN−38 (0.03uM)
Mean Mono Via = 43.1%
Pearson's r: 0.81
Spearman's rho: 0.86
Mean Abs Error: 3.01% Viability
ALMANAC Combos with SN−38 (0.1uM)
Mean Mono Via = 49.3%
Pearson's r: 1
Spearman's rho: 1
Mean Abs Error: 2.23% Viability
ALMANAC Combos with Cisplatin (5uM)
Mean Mono Via = 99.6%
Pearson's r: 0.91
Spearman's rho: 0.89
Mean Abs Error: 6.13% Viability
ALMANAC Combos with Cisplatin (10uM)
Mean Mono Via = 103%
Pearson's r: 1
Spearman's rho: 1
Mean Abs Error: 0.998% Viability
ALMANAC Combos with Cisplatin (20uM)
Mean Mono Via = 37.5%
Pearson's r: 0.77
Spearman's rho: 0.6
Mean Abs Error: 5.19% Viability
ALMANAC Combos with Nelarabine (20uM)
Mean Mono Via = 93.2%
Pearson's r: 0.86
Spearman's rho: 0.76
Mean Abs Error: 7.65% Viability
Predicted % Viability
Observed % Viability
0
20
40
60
80
100

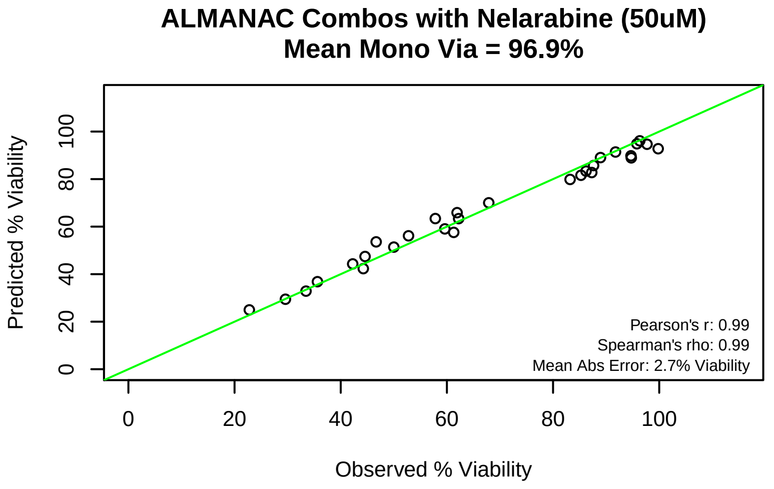
ALMANAC Combos with Nelarabine (50uM)
Mean Mono Via = 96.9%
Predicted % Viability
Observed % Viability
Pearson's r: 0.99
Spearman's rho: 0.99
Mean Abs Error: 2.7% Viability

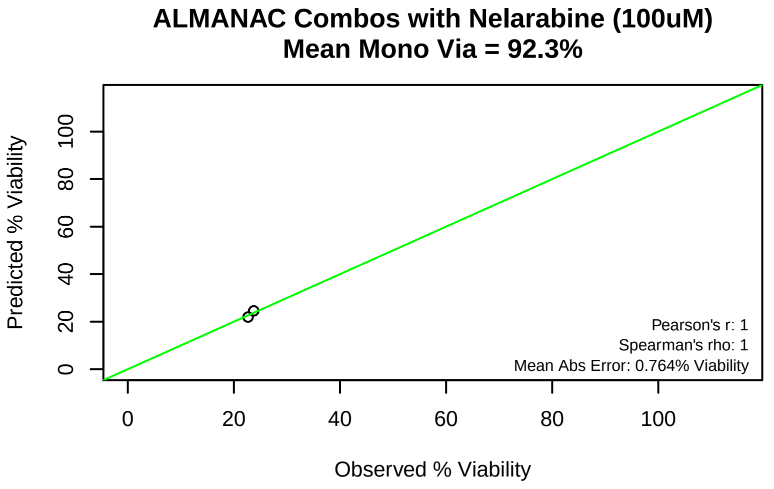
ALMANAC Combos with Nelarabine (100uM)
Mean Mono Via = 92.3%
Predicted % Viability
Observed % Viability
Pearson's r: 1
Spearman's rho: 1
Mean Abs Error: 0.764% Viability

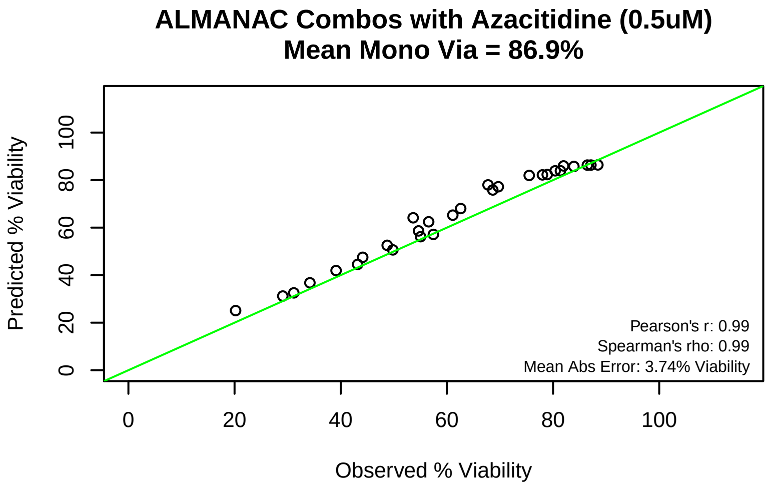
ALMANAC Combos with Azacitidine (0.5uM)
Mean Mono Via = 86.9%
Predicted % Viability
Observed % Viability
Pearson's r: 0.99
Spearman's rho: 0.99
Mean Abs Error: 3.74% Viability

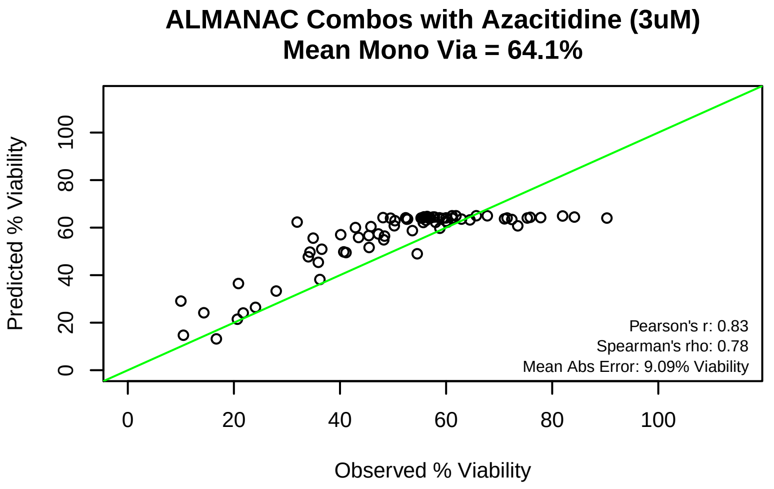
ALMANAC Combos with Azacitidine (3uM)
Mean Mono Via = 64.1%
Predicted % Viability
Observed % Viability
Pearson's r: 0.83
Spearman's rho: 0.78
Mean Abs Error: 9.09% Viability

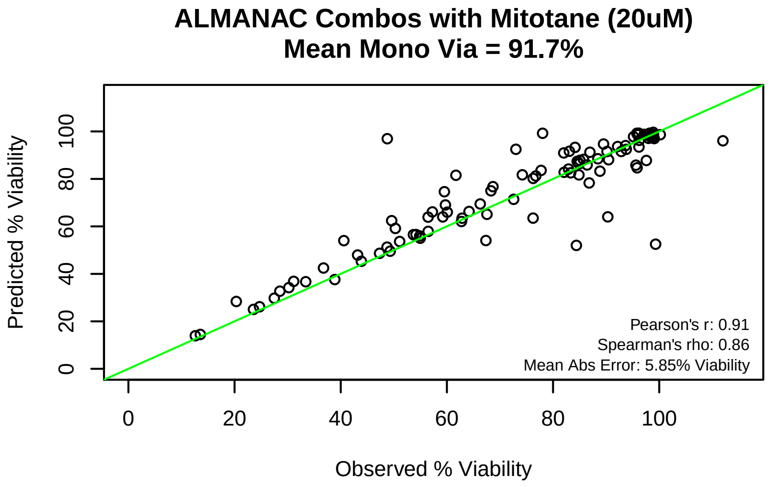
ALMANAC Combos with Mitotane (20uM)
Mean Mono Via = 91.7%
Predicted % Viability
Observed % Viability
Pearson's r: 0.91
Spearman's rho: 0.86
Mean Abs Error: 5.85% Viability

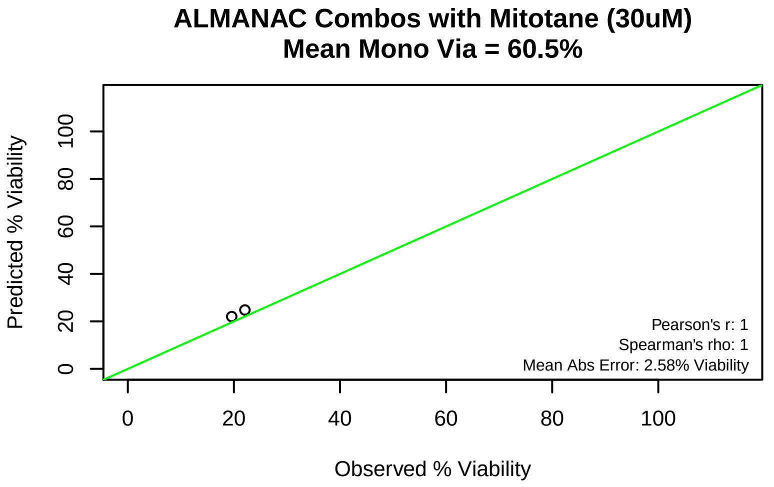
ALMANAC Combos with Mitotane (30uM)
Mean Mono Via = 60.5%
Predicted % Viability
Observed % Viability
Pearson's r: 1
Spearman's rho: 1
Mean Abs Error: 2.58% Viability

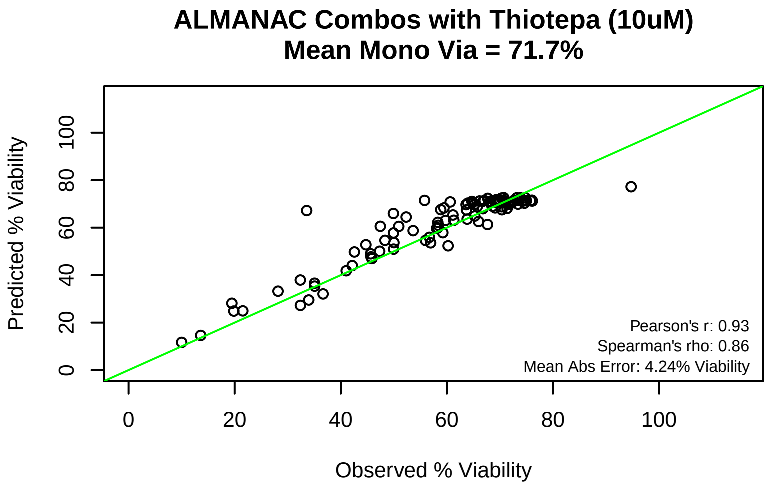
ALMANAC Combos with Thiotepa (10uM)
Mean Mono Via = 71.7%
Predicted % Viability
Observed % Viability
Pearson's r: 0.93
Spearman's rho: 0.86
Mean Abs Error: 4.24% Viability

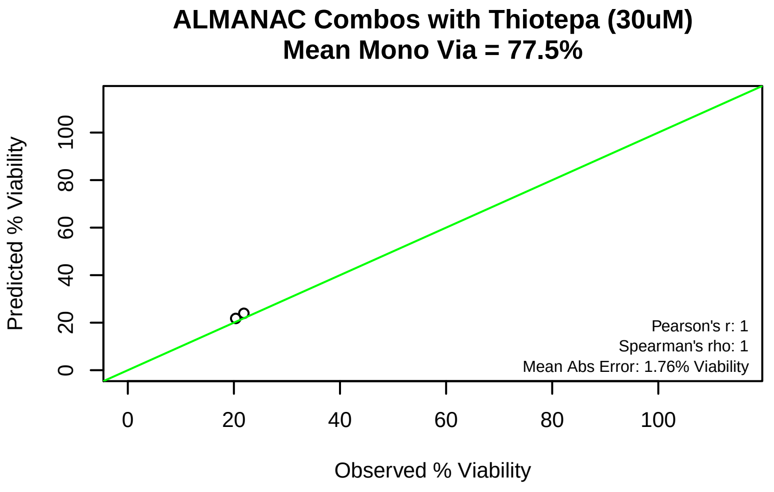
ALMANAC Combos with Thiotepa (30uM)
Mean Mono Via = 77.5%
Predicted % Viability
Observed % Viability
Pearson's r: 1
Spearman's rho: 1
Mean Abs Error: 1.76% Viability

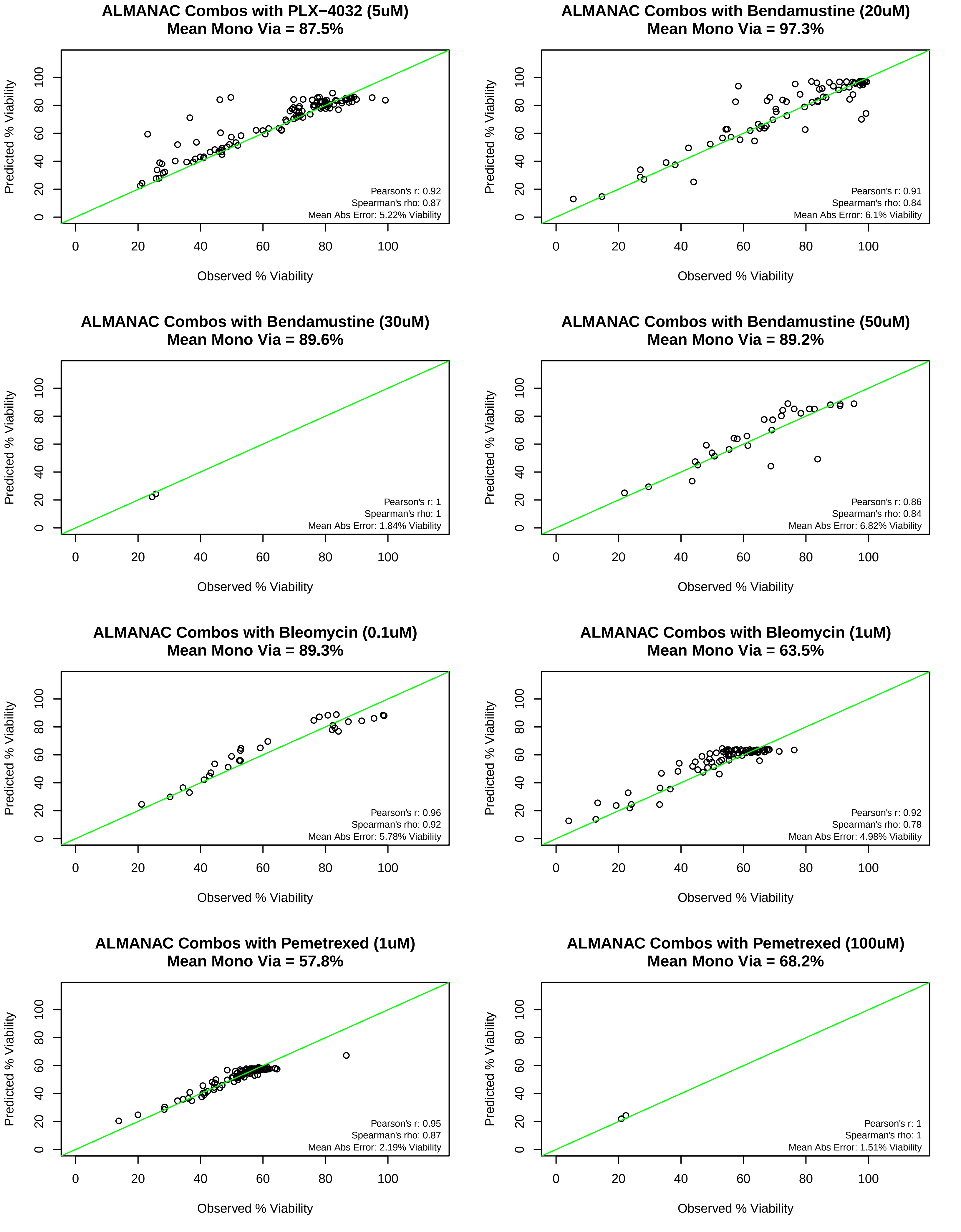
ALMANAC Combos with PLX−4032 (5uM)
Mean Mono Via = 87.5%
Pearson's r: 0.92
Spearman's rho: 0.87
Mean Abs Error: 5.22% Viability
ALMANAC Combos with Bendamustine (20uM)
Mean Mono Via = 97.3%
Pearson's r: 0.91
Spearman's rho: 0.84
Mean Abs Error: 6.1% Viability
ALMANAC Combos with Bendamustine (30uM)
Mean Mono Via = 89.6%
Pearson's r: 1
Spearman's rho: 1
Mean Abs Error: 1.84% Viability
ALMANAC Combos with Bendamustine (50uM)
Mean Mono Via = 89.2%
Pearson's r: 0.86
Spearman's rho: 0.84
Mean Abs Error: 6.82% Viability
ALMANAC Combos with Bleomycin (0.1uM)
Mean Mono Via = 89.3%
Pearson's r: 0.96
Spearman's rho: 0.92
Mean Abs Error: 5.78% Viability
ALMANAC Combos with Bleomycin (1uM)
Mean Mono Via = 63.5%
Pearson's r: 0.92
Spearman's rho: 0.78
Mean Abs Error: 4.98% Viability
ALMANAC Combos with Pemetrexed (1uM)
Mean Mono Via = 57.8%
Pearson's r: 0.95
Spearman's rho: 0.87
Mean Abs Error: 2.19% Viability
ALMANAC Combos with Pemetrexed (100uM)
Mean Mono Via = 68.2%
Pearson's r: 1
Spearman's rho: 1
Mean Abs Error: 1.51% Viability
Predicted % Viability
Observed % Viability
0
20
40
60
80
100

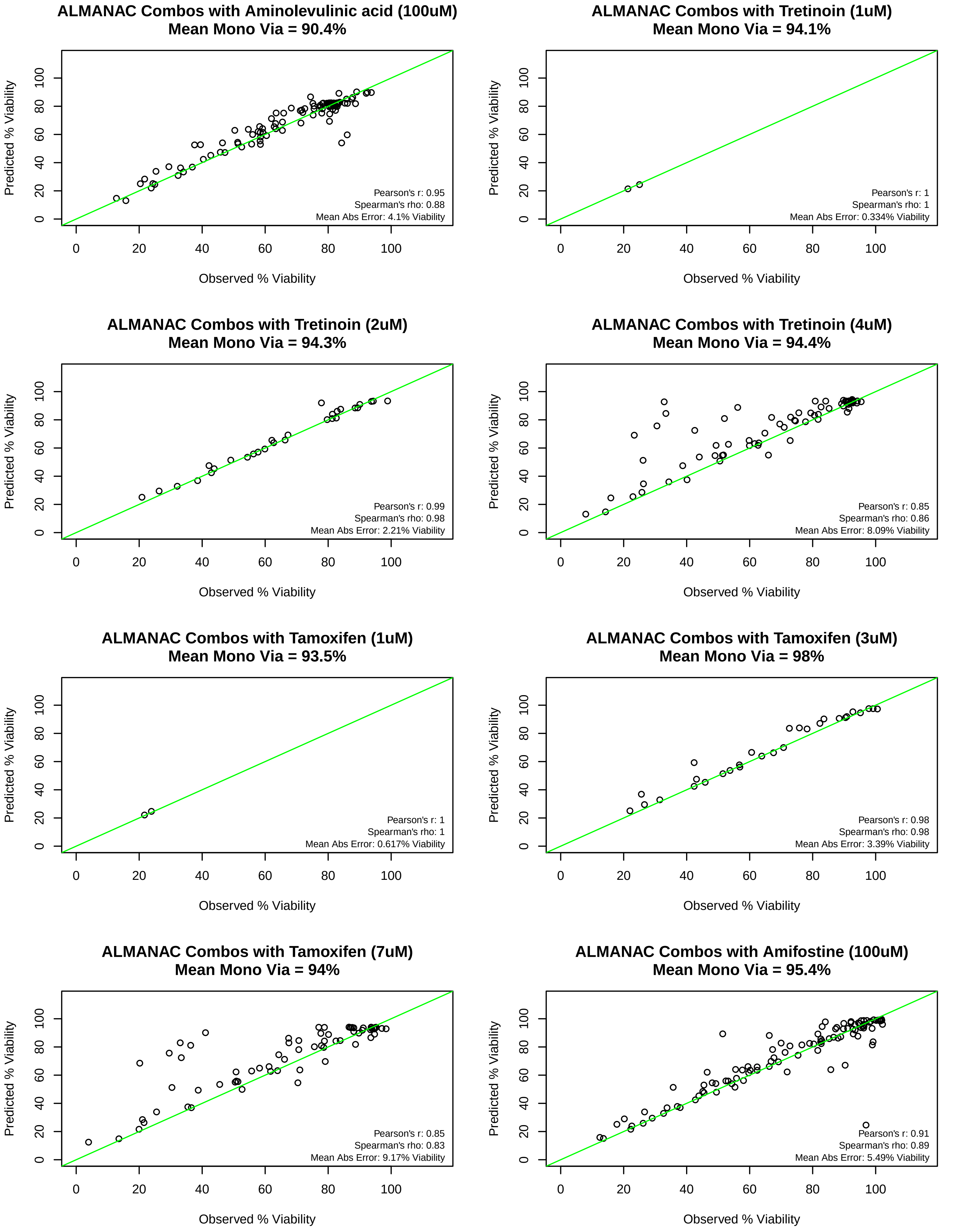
ALMANAC Combos with Aminolevulinic acid (100uM)
Mean Mono Via = 90.4%
Pearson's r: 0.95
Spearman's rho: 0.88
Mean Abs Error: 4.1% Viability
ALMANAC Combos with Tretinoin (1uM)
Mean Mono Via = 94.1%
Pearson's r: 1
Spearman's rho: 1
Mean Abs Error: 0.334% Viability
ALMANAC Combos with Tretinoin (2uM)
Mean Mono Via = 94.3%
Pearson's r: 0.99
Spearman's rho: 0.98
Mean Abs Error: 2.21% Viability
ALMANAC Combos with Tretinoin (4uM)
Mean Mono Via = 94.4%
Pearson's r: 0.85
Spearman's rho: 0.86
Mean Abs Error: 8.09% Viability
ALMANAC Combos with Tamoxifen (1uM)
Mean Mono Via = 93.5%
Pearson's r: 1
Spearman's rho: 1
Mean Abs Error: 0.617% Viability
ALMANAC Combos with Tamoxifen (3uM)
Mean Mono Via = 98%
Pearson's r: 0.98
Spearman's rho: 0.98
Mean Abs Error: 3.39% Viability
ALMANAC Combos with Tamoxifen (7uM)
Mean Mono Via = 94%
Pearson's r: 0.85
Spearman's rho: 0.83
Mean Abs Error: 9.17% Viability
ALMANAC Combos with Amifostine (100uM)
Mean Mono Via = 95.4%
Pearson's r: 0.91
Spearman's rho: 0.89
Mean Abs Error: 5.49% Viability
Predicted % Viability
Observed % Viability
0
20
40
60
80
100

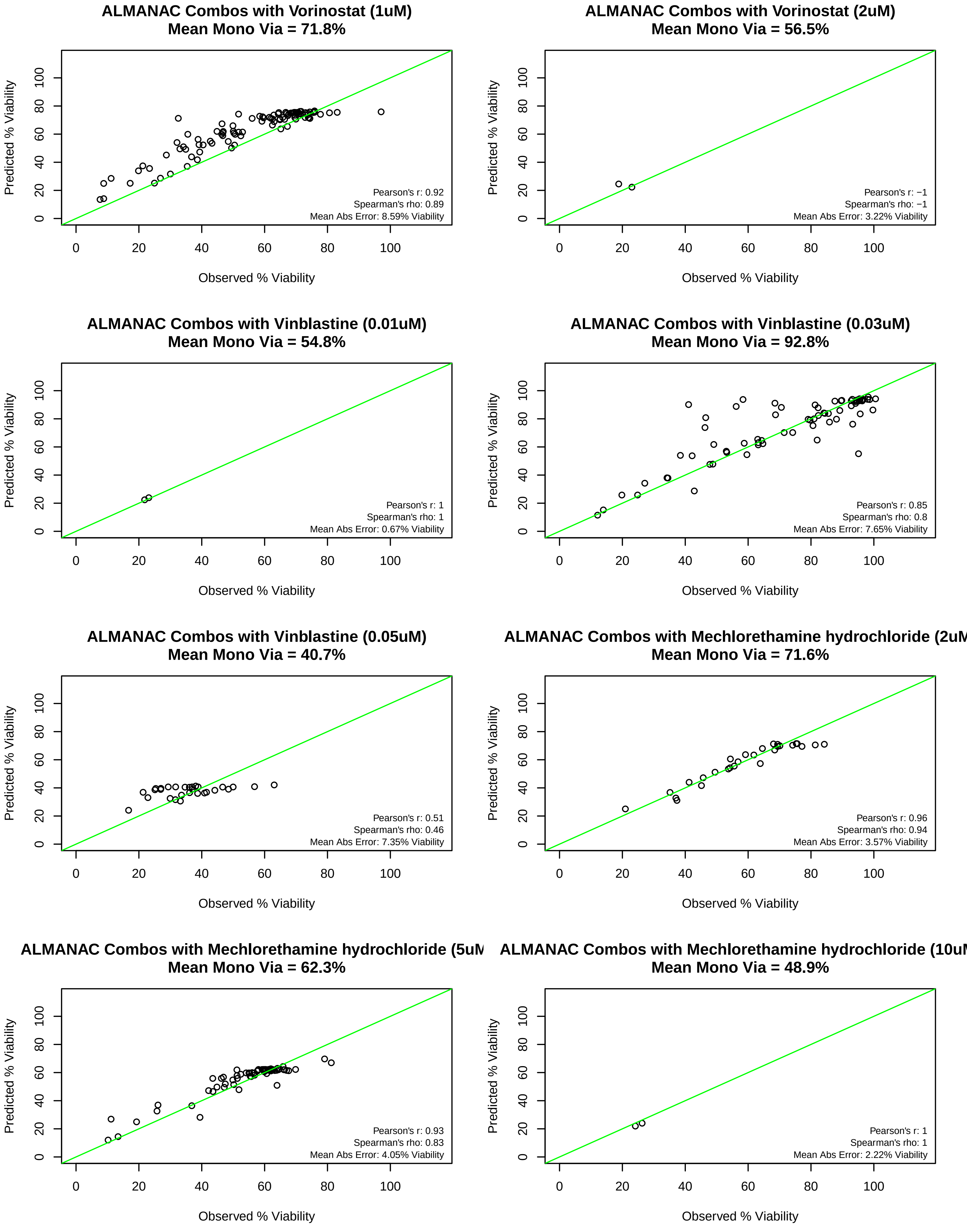
ALMANAC Combos with Vorinostat (1uM)
Mean Mono Via = 71.8%
Pearson's r: 0.92
Spearman's rho: 0.89
Mean Abs Error: 8.59% Viability
ALMANAC Combos with Vorinostat (2uM)
Mean Mono Via = 56.5%
Pearson's r: −1
Spearman's rho: −1
Mean Abs Error: 3.22% Viability
ALMANAC Combos with Vinblastine (0.01uM)
Mean Mono Via = 54.8%
Pearson's r: 1
Spearman's rho: 1
Mean Abs Error: 0.67% Viability
ALMANAC Combos with Vinblastine (0.03uM)
Mean Mono Via = 92.8%
Pearson's r: 0.85
Spearman's rho: 0.8
Mean Abs Error: 7.65% Viability
ALMANAC Combos with Vinblastine (0.05uM)
Mean Mono Via = 40.7%
Pearson's r: 0.51
Spearman's rho: 0.46
Mean Abs Error: 7.35% Viability
ALMANAC Combos with Mechlorethamine hydrochloride (2uM
Mean Mono Via = 71.6%
Pearson's r: 0.96
Spearman's rho: 0.94
Mean Abs Error: 3.57% Viability
ALMANAC Combos with Mechlorethamine hydrochloride (5uM
Mean Mono Via = 62.3%
Pearson's r: 0.93
Spearman's rho: 0.83
Mean Abs Error: 4.05% Viability
ALMANAC Combos with Mechlorethamine hydrochloride (10uM
Mean Mono Via = 48.9%
Pearson's r: 1
Spearman's rho: 1
Mean Abs Error: 2.22% Viability
Predicted % Viability
Observed % Viability

ALMANAC Combos with Dacarbazine (30uM)
Mean Mono Via = 75.4%

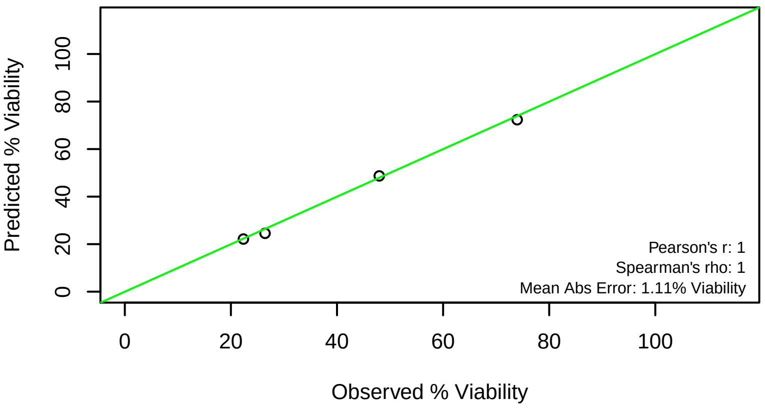

ALMANAC Combos with Dacarbazine (50uM)
Mean Mono Via = 92.4%

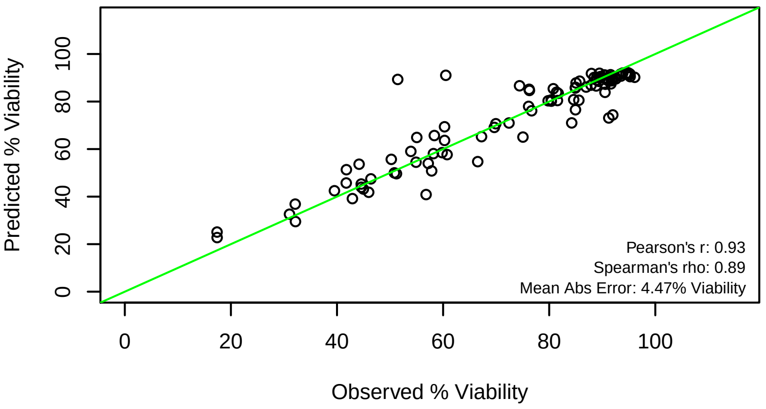

ALMANAC Combos with Carmustine (15uM)
Mean Mono Via = 92.2%

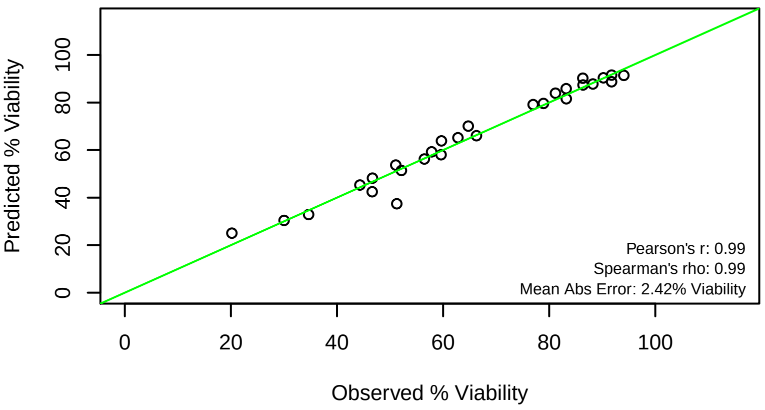

ALMANAC Combos with Carmustine (20uM)
Mean Mono Via = 88.3%

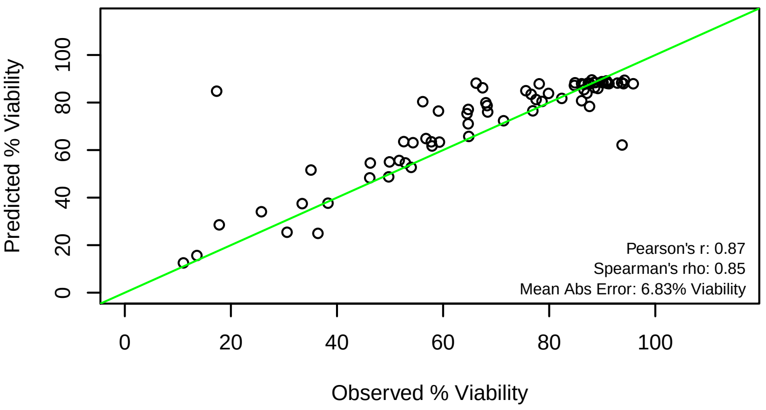

ALMANAC Combos with Carmustine (50uM)
Mean Mono Via = 77.8%

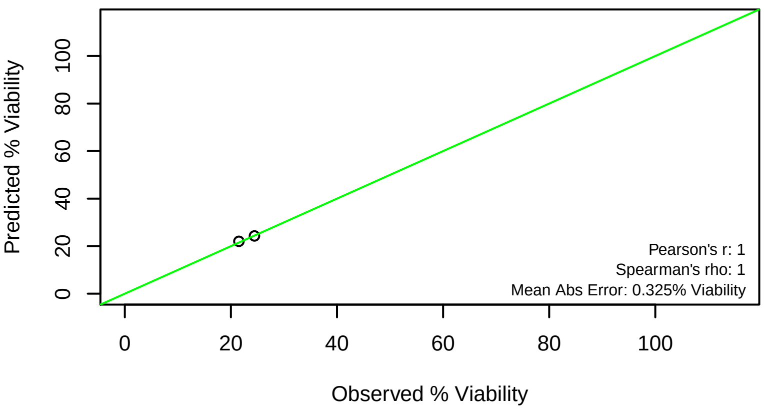

ALMANAC Combos with Oxaliplatin (1uM)
Mean Mono Via = 78.4%

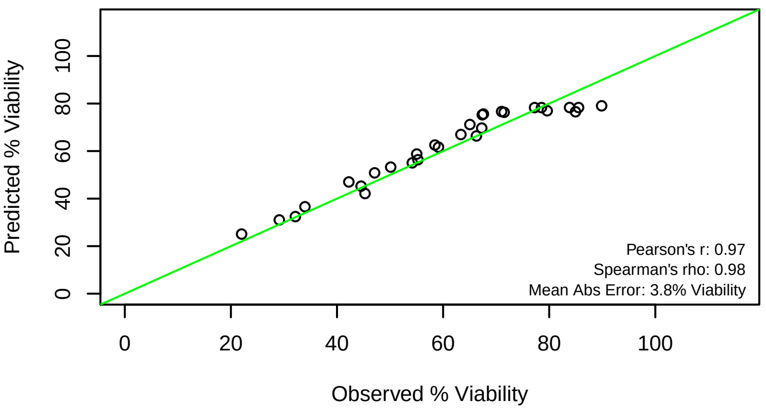

ALMANAC Combos with Oxaliplatin (5uM)
Mean Mono Via = 63.4%

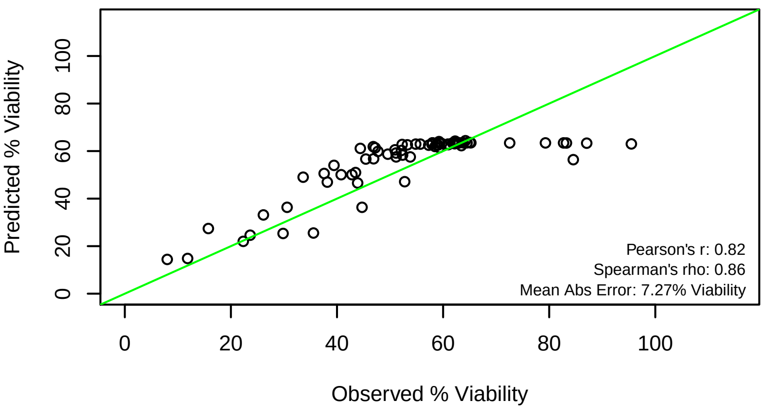

ALMANAC Combos with Sorafenib (10uM)
Mean Mono Via = 45.8%

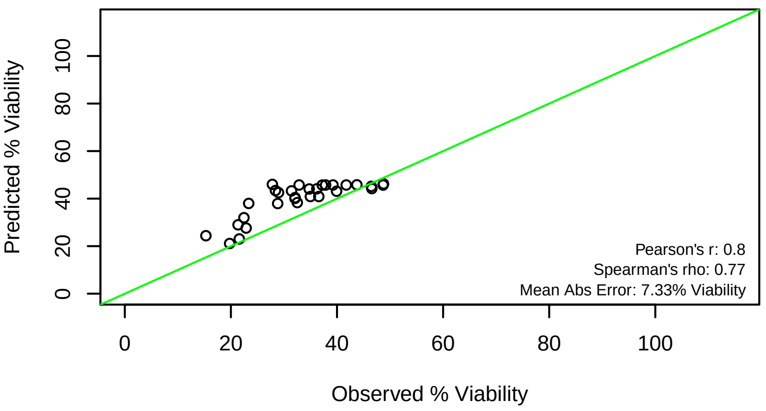

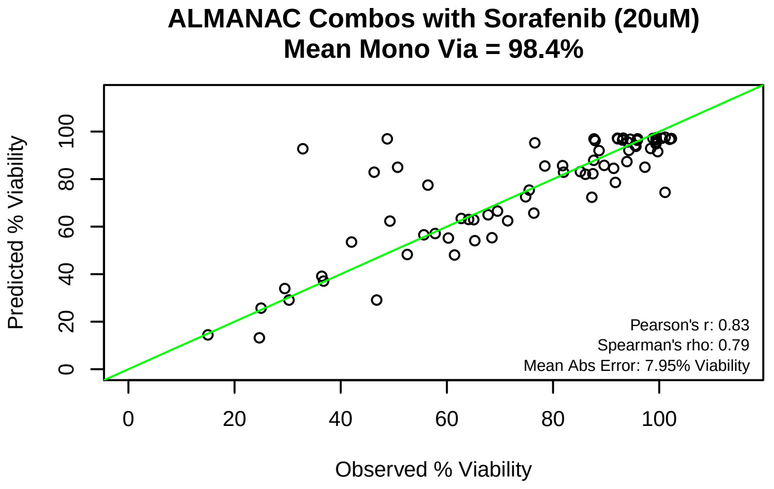
ALMANAC Combos with Sorafenib (20uM)
Mean Mono Via = 98.4%
Pearson's r: 0.83
Spearman's rho: 0.79
Mean Abs Error: 7.95% Viability
Predicted % Viability
Observed % Viability

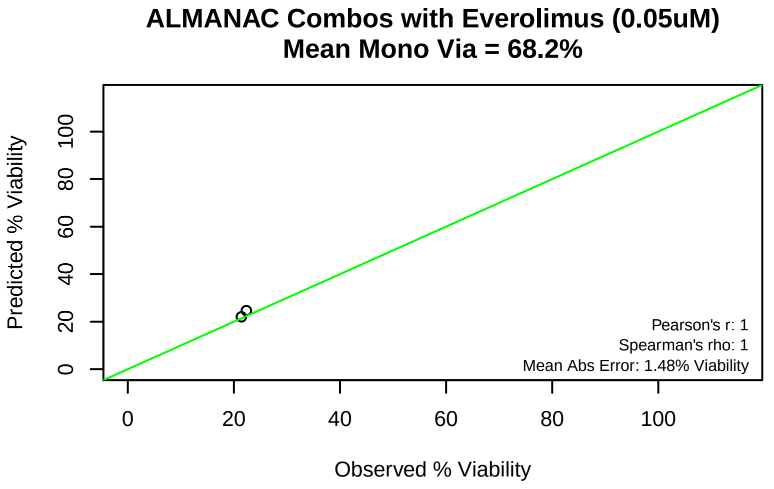
ALMANAC Combos with Everolimus (0.05uM)
Mean Mono Via = 68.2%
Pearson's r: 1
Spearman's rho: 1
Mean Abs Error: 1.48% Viability
Predicted % Viability
Observed % Viability

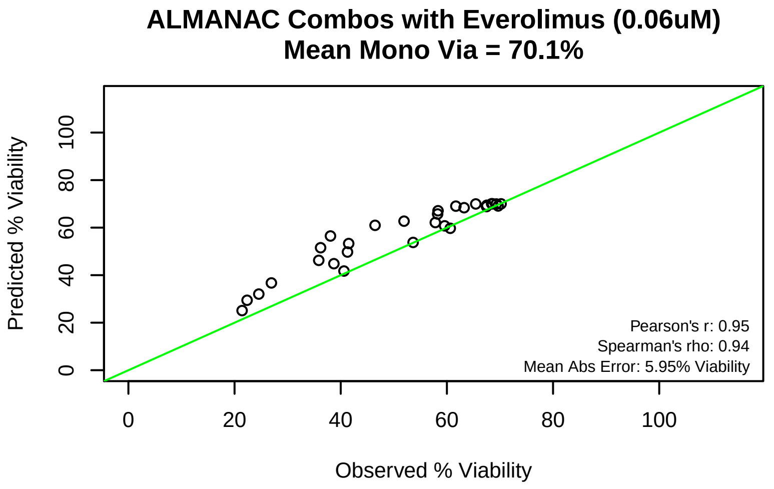
ALMANAC Combos with Everolimus (0.06uM)
Mean Mono Via = 70.1%
Pearson's r: 0.95
Spearman's rho: 0.94
Mean Abs Error: 5.95% Viability
Predicted % Viability
Observed % Viability

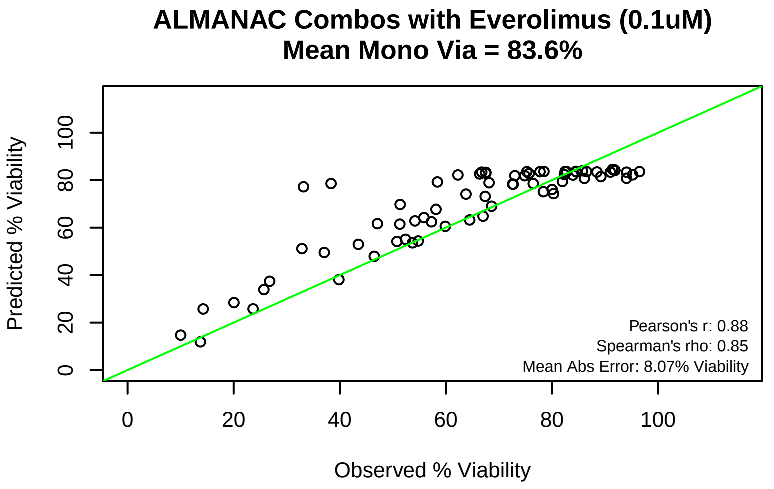
ALMANAC Combos with Everolimus (0.1uM)
Mean Mono Via = 83.6%
Pearson's r: 0.88
Spearman's rho: 0.85
Mean Abs Error: 8.07% Viability
Predicted % Viability
Observed % Viability

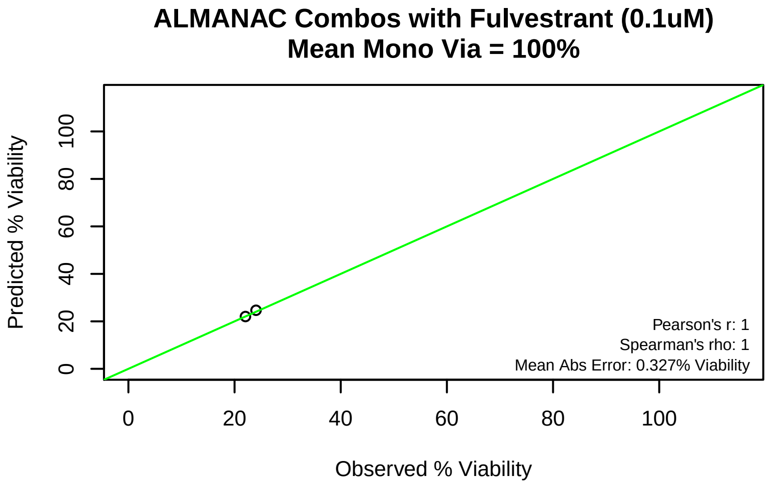
ALMANAC Combos with Fulvestrant (0.1uM)
Mean Mono Via = 100%
Pearson's r: 1
Spearman's rho: 1
Mean Abs Error: 0.327% Viability
Predicted % Viability
Observed % Viability

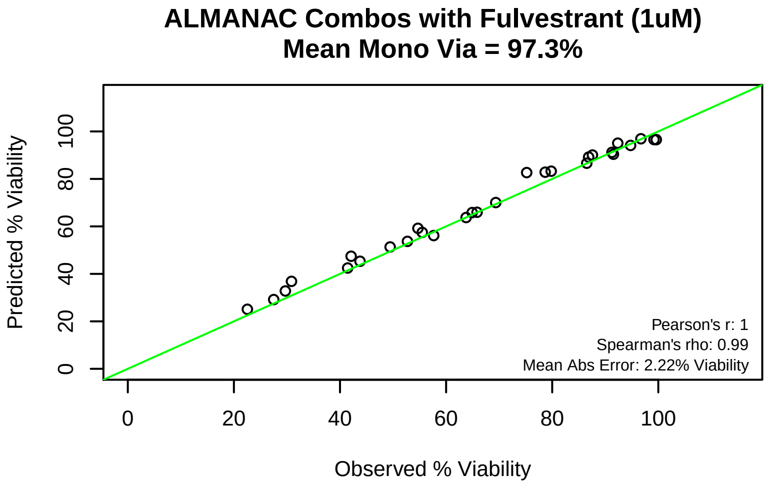
ALMANAC Combos with Fulvestrant (1uM)
Mean Mono Via = 97.3%
Pearson's r: 1
Spearman's rho: 0.99
Mean Abs Error: 2.22% Viability
Predicted % Viability
Observed % Viability

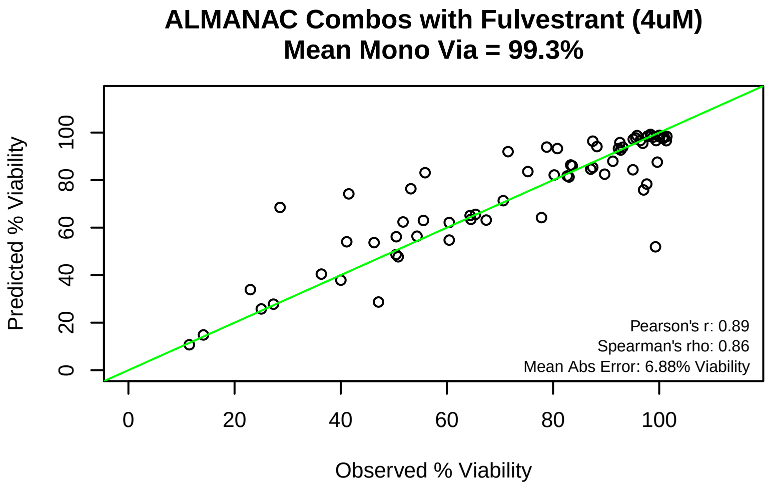
ALMANAC Combos with Fulvestrant (4uM)
Mean Mono Via = 99.3%
Pearson's r: 0.89
Spearman's rho: 0.86
Mean Abs Error: 6.88% Viability
Predicted % Viability
Observed % Viability

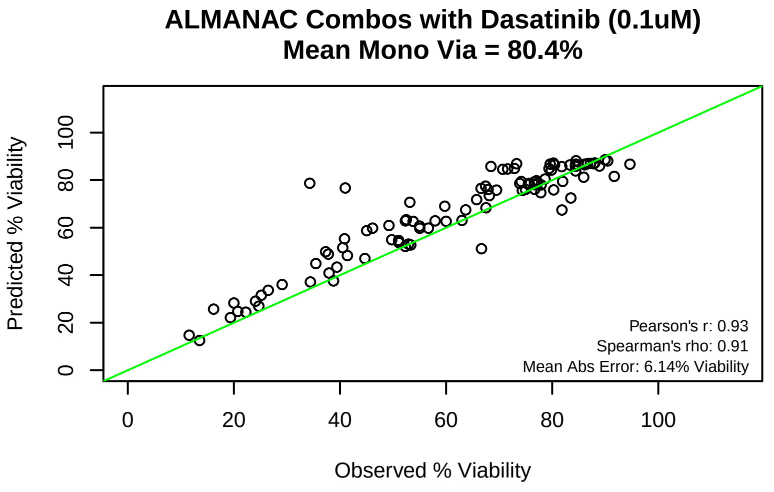
ALMANAC Combos with Dasatinib (0.1uM)
Mean Mono Via = 80.4%
Pearson's r: 0.93
Spearman's rho: 0.91
Mean Abs Error: 6.14% Viability
Predicted % Viability
Observed % Viability

ALMANAC Combos with Vismodegib (25uM)
Mean Mono Via = 89.7%

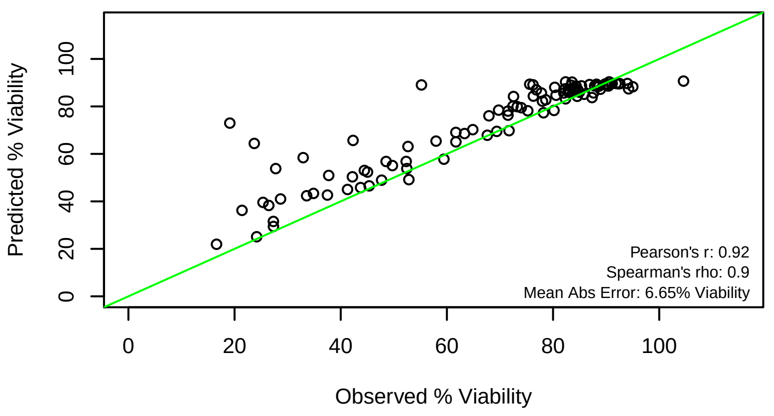

ALMANAC Combos with Vismodegib (30uM)
Mean Mono Via = 87.6%

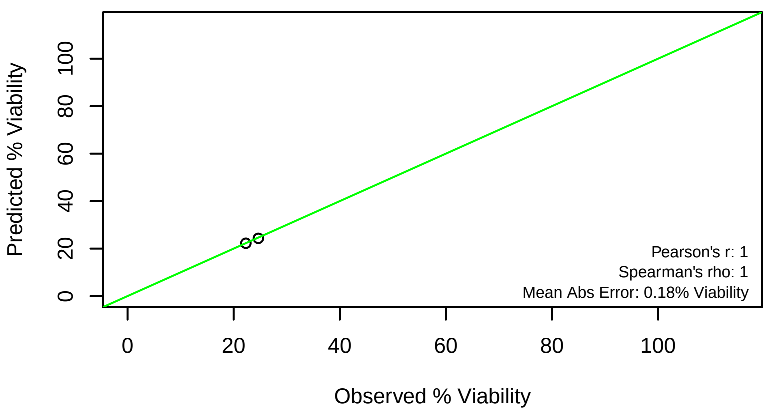

ALMANAC Combos with Floxuridine (0.07uM)
Mean Mono Via = 66.3%

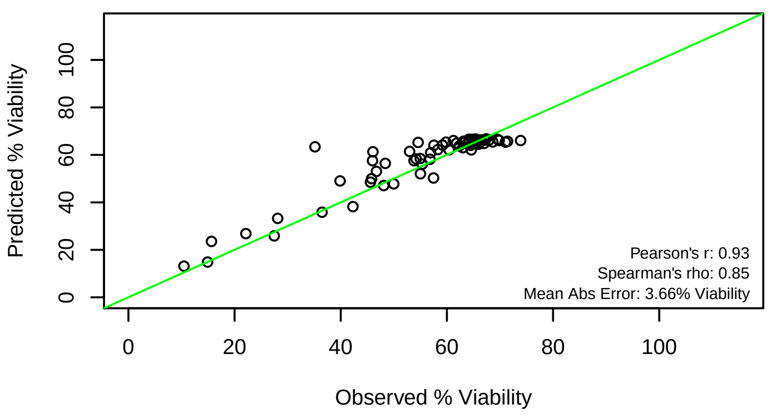

ALMANAC Combos with Floxuridine (0.7uM)
Mean Mono Via = 49.8%

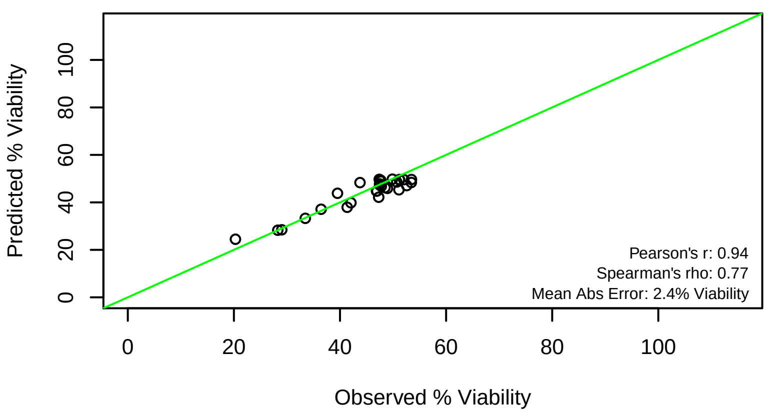

ALMANAC Combos with Floxuridine (1uM)
Mean Mono Via = 59.8%

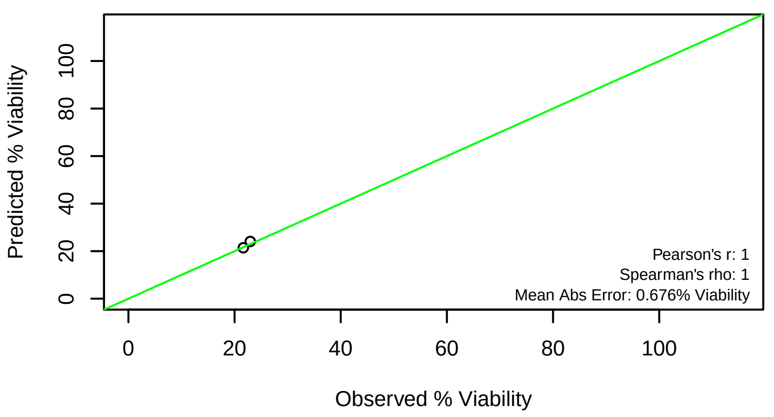

ALMANAC Combos with Procarbazine hydrochloride (3uM)
Mean Mono Via = 96%

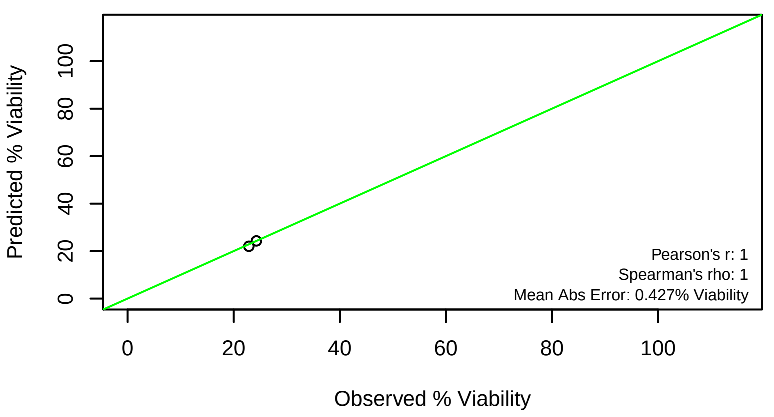

ALMANAC Combos with Procarbazine hydrochloride (10uM)
Mean Mono Via = 98.9%

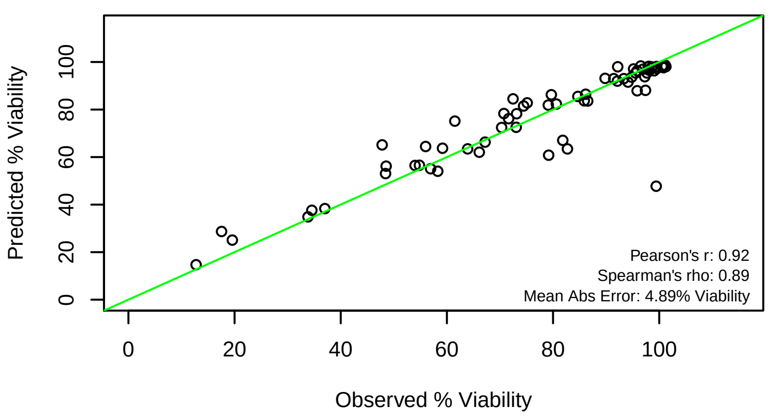

ALMANAC Combos with Procarbazine hydrochloride (20uM)
Mean Mono Via = 98.6%

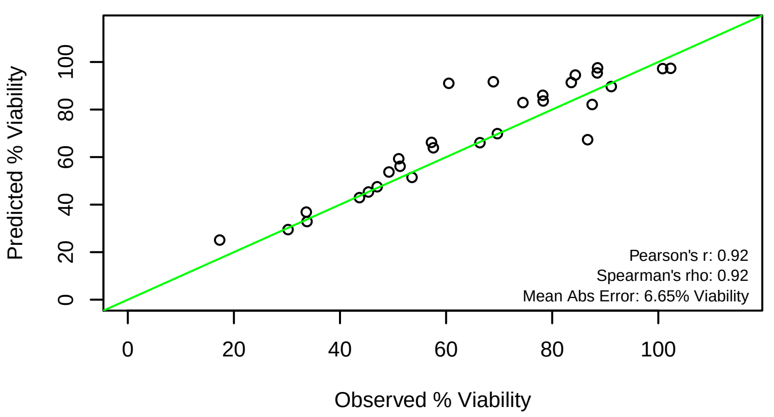

ALMANAC Combos with Cladribine (0.2uM)
Mean Mono Via = 84.8%

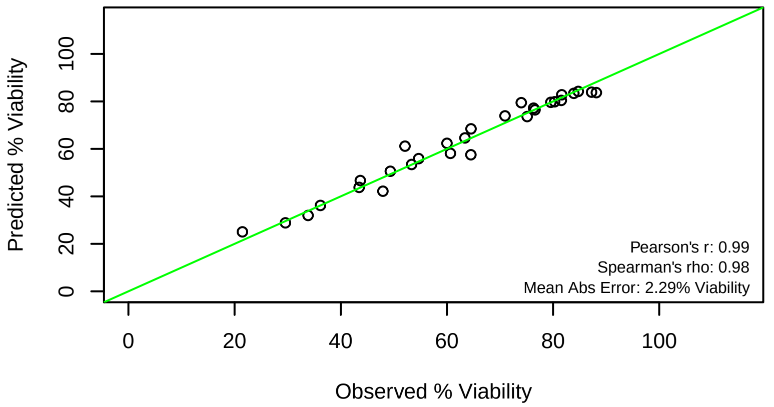

ALMANAC Combos with Cladribine (1uM)
Mean Mono Via = 56.4%

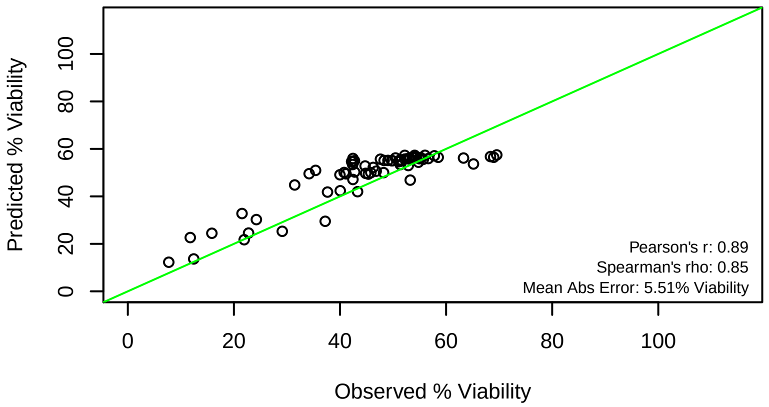

ALMANAC Combos with Abiraterone (5uM)
Mean Mono Via = 86.6%

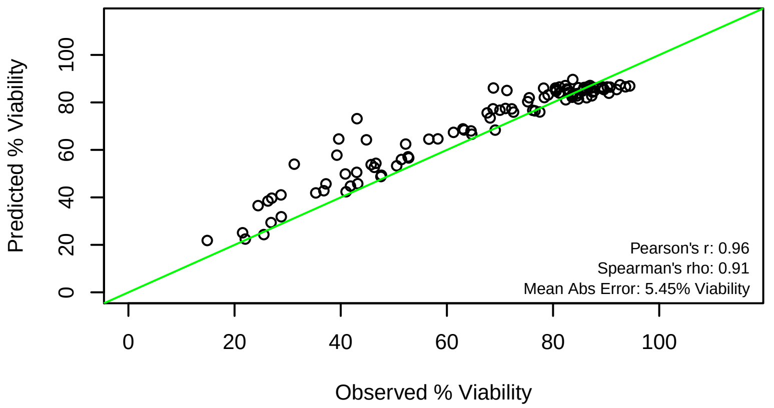

ALMANAC Combos with Cytarabine (0.5uM)
Mean Mono Via = 58.4%

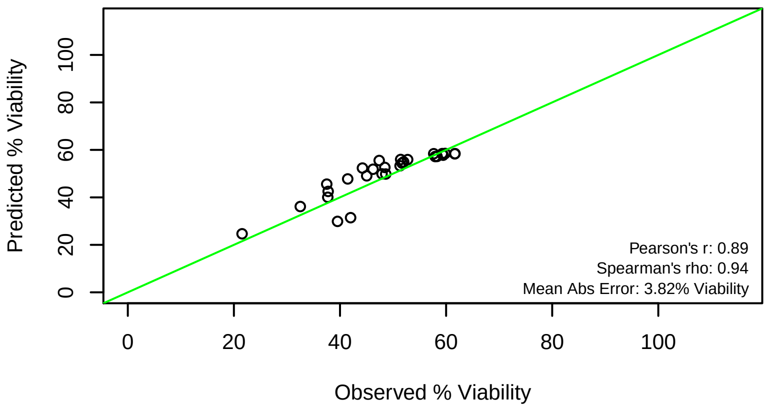

ALMANAC Combos with Cytarabine (1uM)
Mean Mono Via = 56.2%

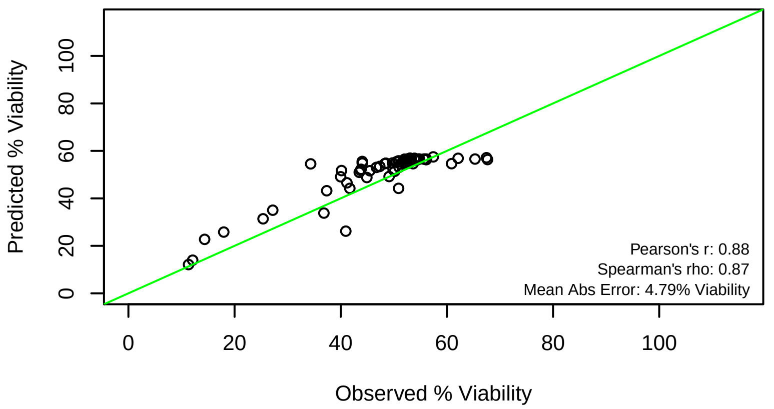

ALMANAC Combos with Cytarabine (10uM)
Mean Mono Via = 61.4%

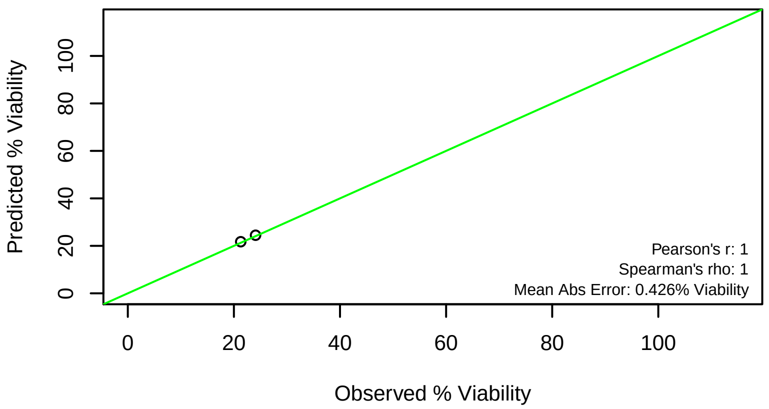

ALMANAC Combos with Cyclophosphamide (100uM)
Mean Mono Via = 102%

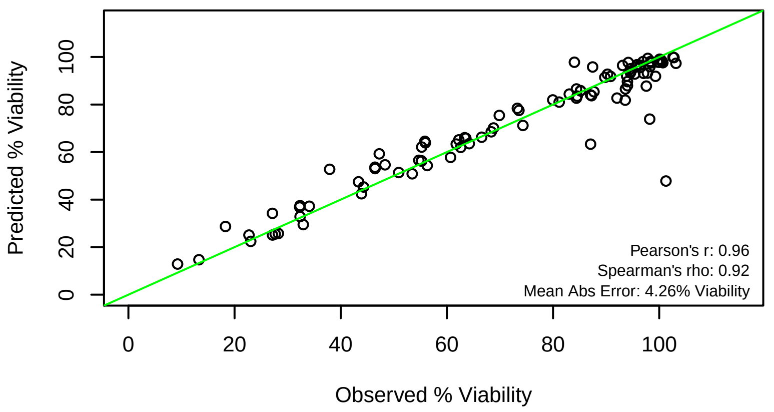

ALMANAC Combos with Vandetanib (2uM)
Mean Mono Via = 84%

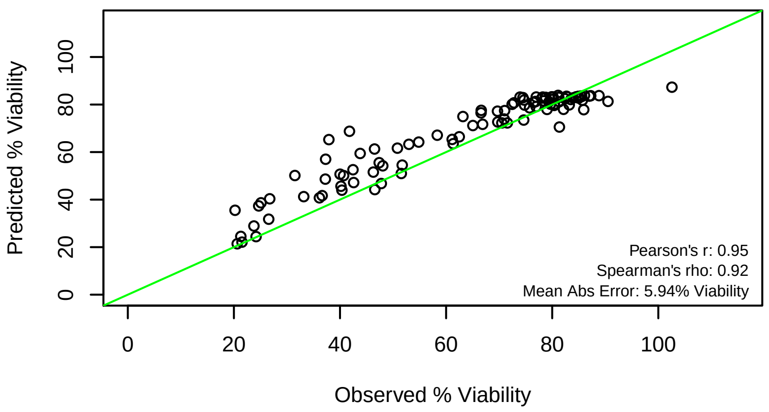

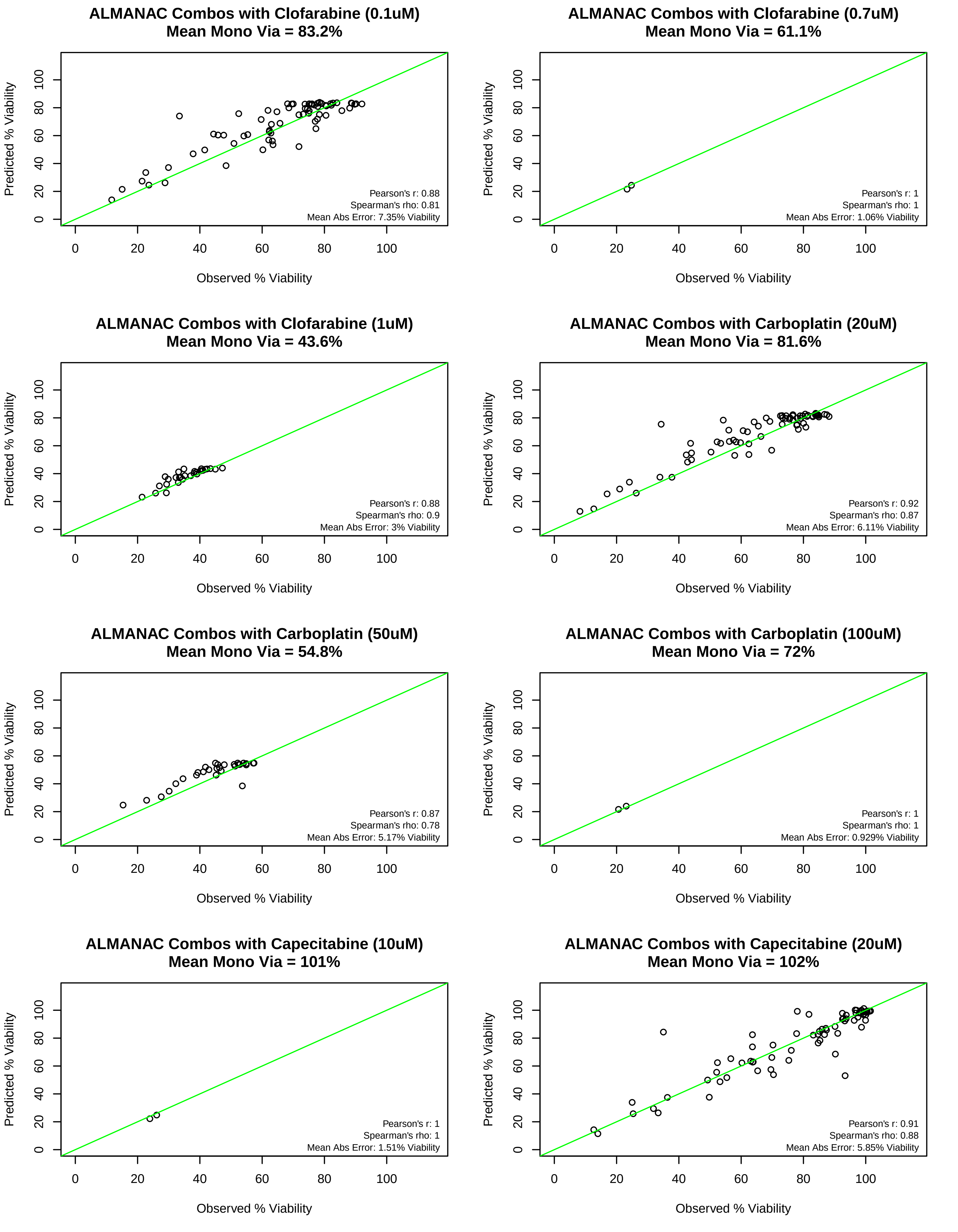
ALMANAC Combos with Clofarabine (0.1uM)
Mean Mono Via = 83.2%
Pearson's r: 0.88
Spearman's rho: 0.81
Mean Abs Error: 7.35% Viability
ALMANAC Combos with Clofarabine (0.7uM)
Mean Mono Via = 61.1%
Pearson's r: 1
Spearman's rho: 1
Mean Abs Error: 1.06% Viability
ALMANAC Combos with Clofarabine (1uM)
Mean Mono Via = 43.6%
Pearson's r: 0.88
Spearman's rho: 0.9
Mean Abs Error: 3% Viability
ALMANAC Combos with Carboplatin (20uM)
Mean Mono Via = 81.6%
Pearson's r: 0.92
Spearman's rho: 0.87
Mean Abs Error: 6.11% Viability
ALMANAC Combos with Carboplatin (50uM)
Mean Mono Via = 54.8%
Pearson's r: 0.87
Spearman's rho: 0.78
Mean Abs Error: 5.17% Viability
ALMANAC Combos with Carboplatin (100uM)
Mean Mono Via = 72%
Pearson's r: 1
Spearman's rho: 1
Mean Abs Error: 0.929% Viability
ALMANAC Combos with Capecitabine (10uM)
Mean Mono Via = 101%
Pearson's r: 1
Spearman's rho: 1
Mean Abs Error: 1.51% Viability
ALMANAC Combos with Capecitabine (20uM)
Mean Mono Via = 102%
Pearson's r: 0.91
Spearman's rho: 0.88
Mean Abs Error: 5.85% Viability
Predicted % Viability
Observed % Viability
0
20
40
60
80
100

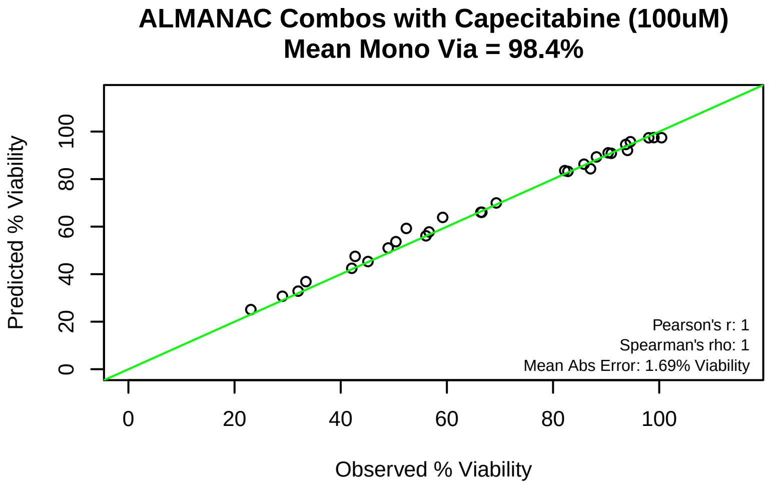
ALMANAC Combos with Capecitabine (100uM)
Mean Mono Via = 98.4%
Predicted % Viability
Observed % Viability
Pearson's r: 1
Spearman's rho: 1
Mean Abs Error: 1.69% Viability

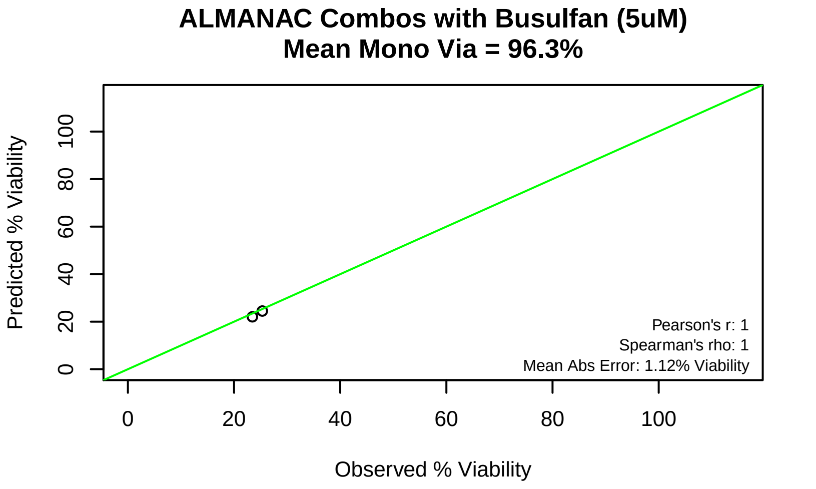
ALMANAC Combos with Busulfan (5uM)
Mean Mono Via = 96.3%
Predicted % Viability
Observed % Viability
Pearson's r: 1
Spearman's rho: 1
Mean Abs Error: 1.12% Viability

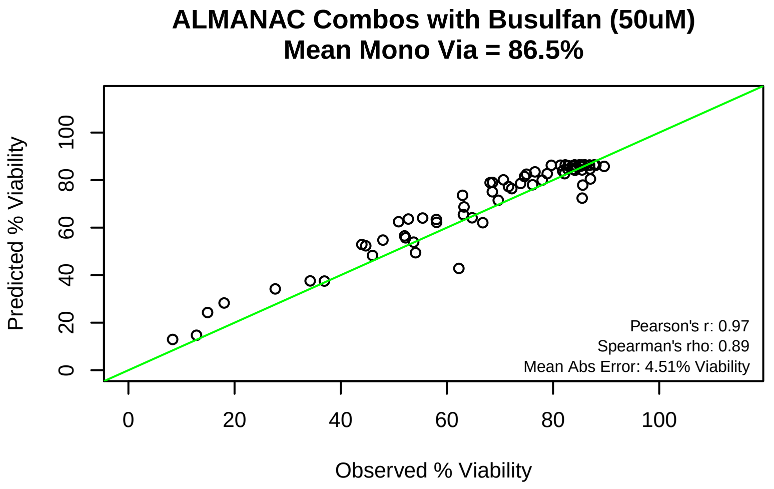
ALMANAC Combos with Busulfan (50uM)
Mean Mono Via = 86.5%
Predicted % Viability
Observed % Viability
Pearson's r: 0.97
Spearman's rho: 0.89
Mean Abs Error: 4.51% Viability

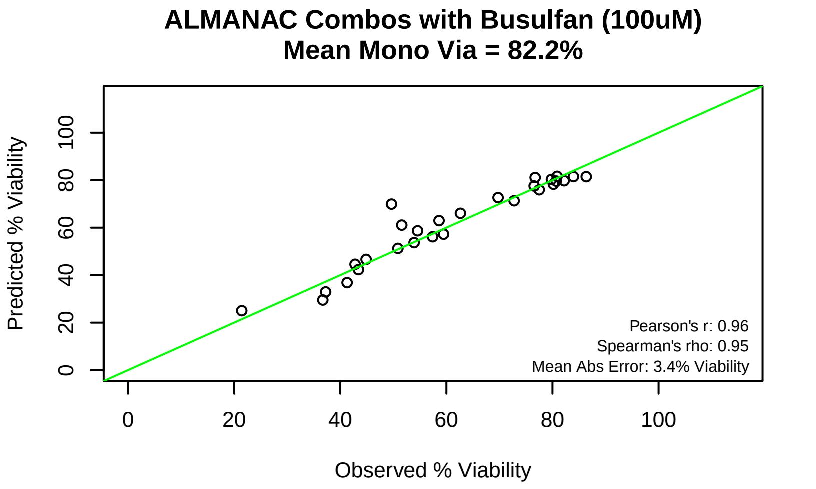
ALMANAC Combos with Busulfan (100uM)
Mean Mono Via = 82.2%
Predicted % Viability
Observed % Viability
Pearson's r: 0.96
Spearman's rho: 0.95
Mean Abs Error: 3.4% Viability

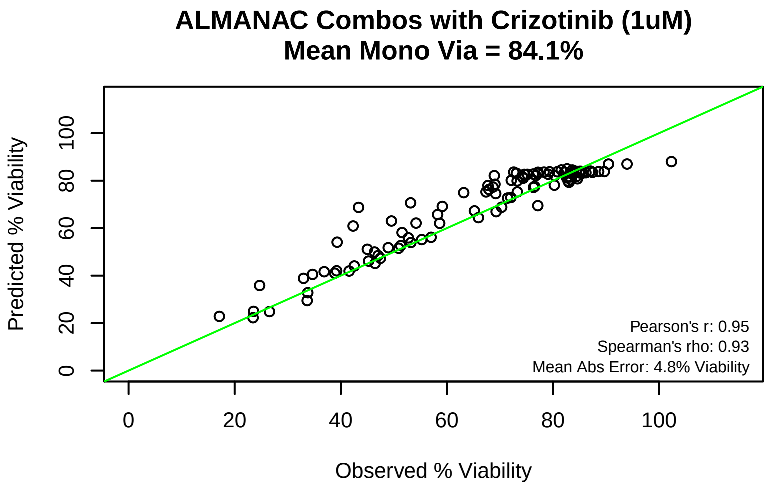
ALMANAC Combos with Crizotinib (1uM)
Mean Mono Via = 84.1%
Predicted % Viability
Observed % Viability
Pearson's r: 0.95
Spearman's rho: 0.93
Mean Abs Error: 4.8% Viability

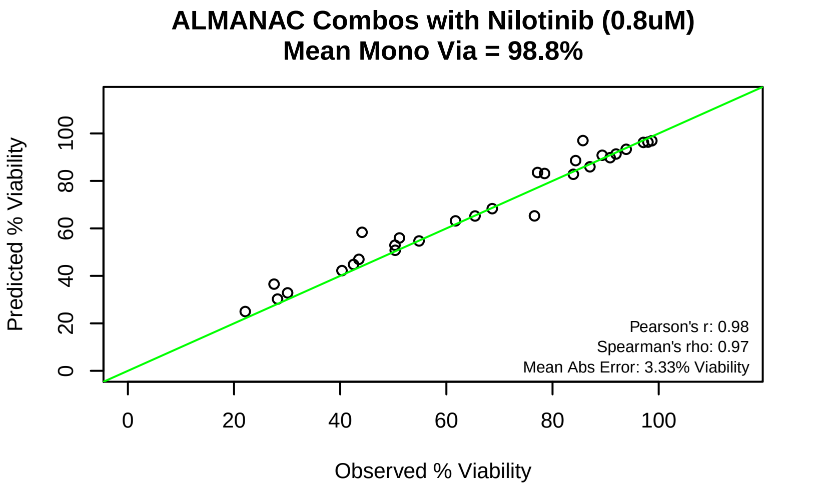
ALMANAC Combos with Nilotinib (0.8uM)
Mean Mono Via = 98.8%
Predicted % Viability
Observed % Viability
Pearson's r: 0.98
Spearman's rho: 0.97
Mean Abs Error: 3.33% Viability

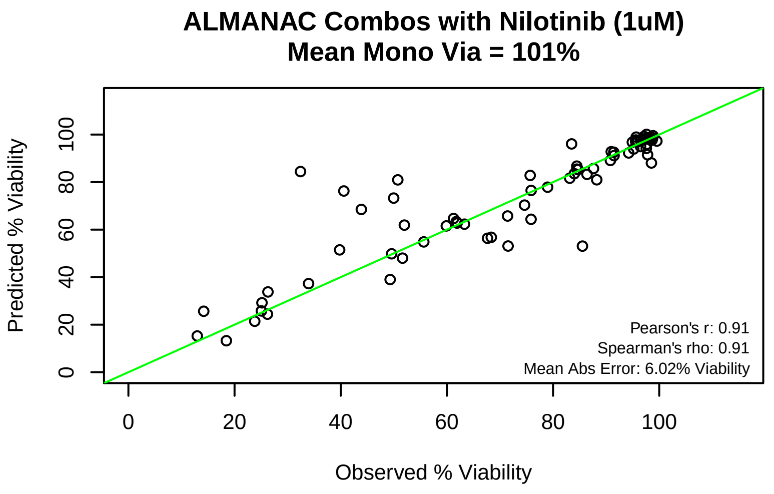
ALMANAC Combos with Nilotinib (1uM)
Mean Mono Via = 101%
Predicted % Viability
Observed % Viability
Pearson's r: 0.91
Spearman's rho: 0.91
Mean Abs Error: 6.02% Viability

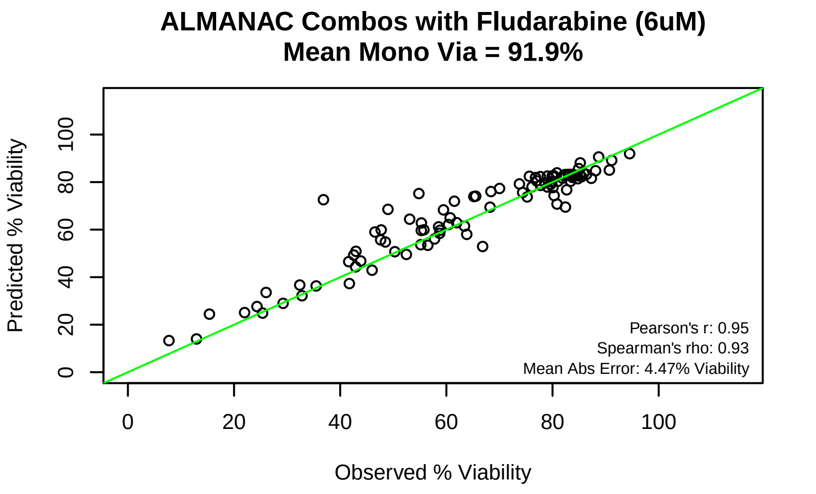
ALMANAC Combos with Fludarabine (6uM)
Mean Mono Via = 91.9%
Predicted % Viability
Observed % Viability
Pearson's r: 0.95
Spearman's rho: 0.93
Mean Abs Error: 4.47% Viability

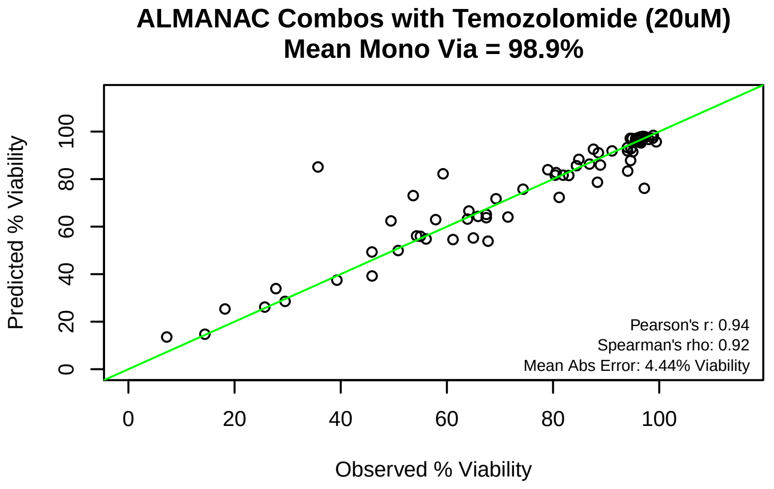
ALMANAC Combos with Temozolomide (20uM)
Mean Mono Via = 98.9%
Predicted % Viability
Observed % Viability
Pearson's r: 0.94
Spearman's rho: 0.92
Mean Abs Error: 4.44% Viability

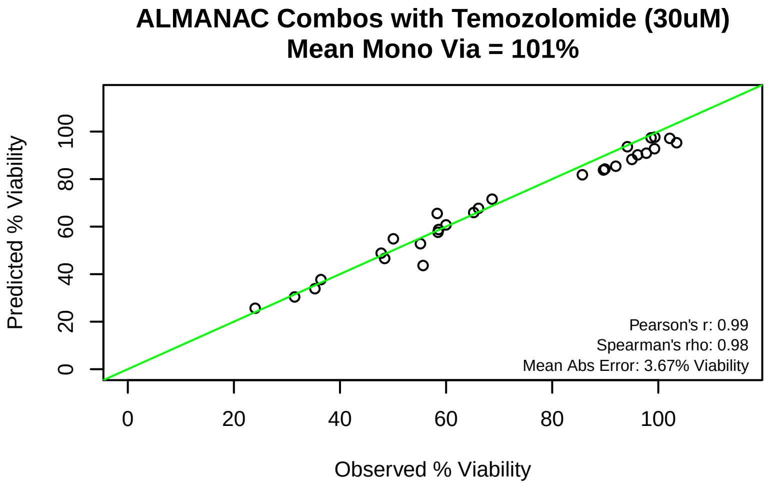
ALMANAC Combos with Temozolomide (30uM)
Mean Mono Via = 101%
Predicted % Viability
Observed % Viability
Pearson's r: 0.99
Spearman's rho: 0.98
Mean Abs Error: 3.67% Viability

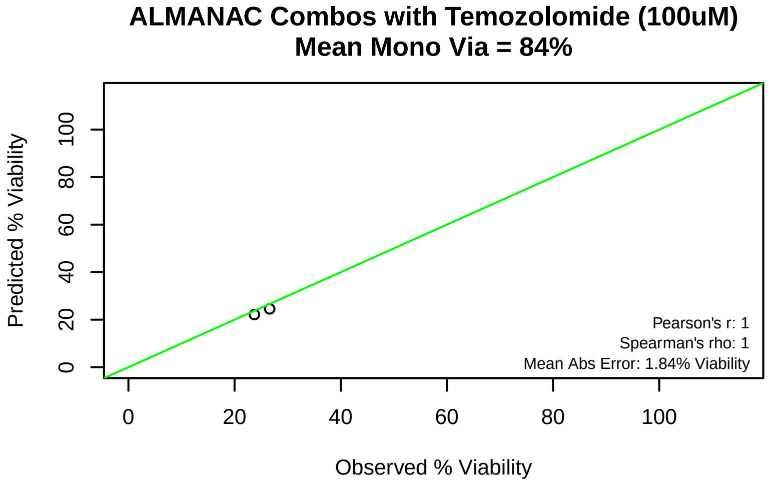
ALMANAC Combos with Temozolomide (100uM)
Mean Mono Via = 84%
Predicted % Viability
Observed % Viability
Pearson's r: 1
Spearman's rho: 1
Mean Abs Error: 1.84% Viability

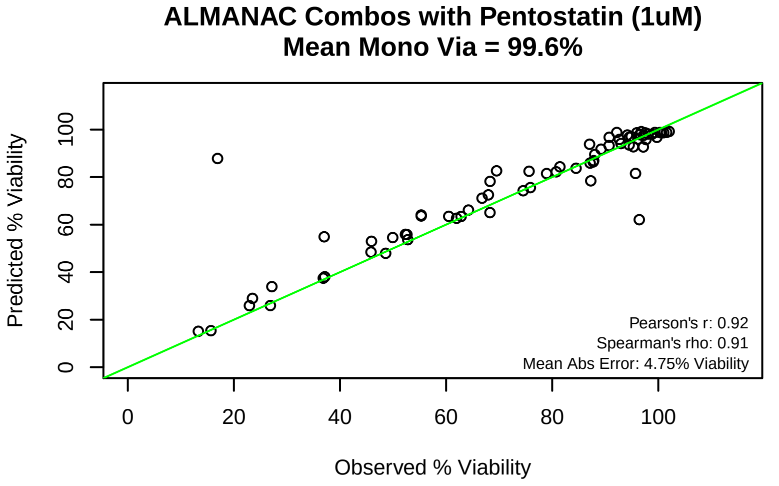
ALMANAC Combos with Pentostatin (1uM)
Mean Mono Via = 99.6%
Predicted % Viability
Observed % Viability
Pearson's r: 0.92
Spearman's rho: 0.91
Mean Abs Error: 4.75% Viability

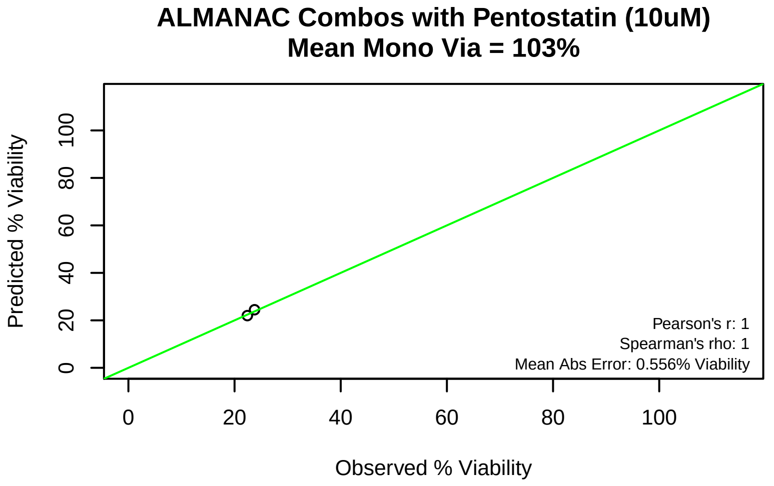
ALMANAC Combos with Pentostatin (10uM)
Mean Mono Via = 103%
Predicted % Viability
Observed % Viability
Pearson's r: 1
Spearman's rho: 1
Mean Abs Error: 0.556% Viability

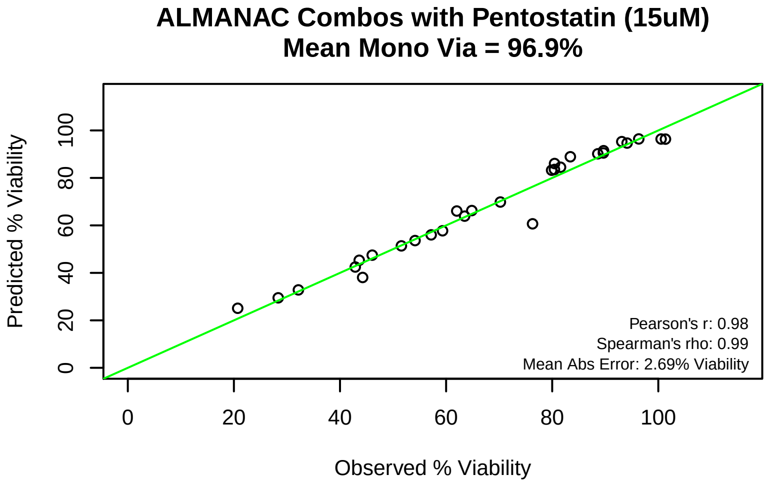
ALMANAC Combos with Pentostatin (15uM)
Mean Mono Via = 96.9%
Predicted % Viability
Observed % Viability
Pearson's r: 0.98
Spearman's rho: 0.99
Mean Abs Error: 2.69% Viability

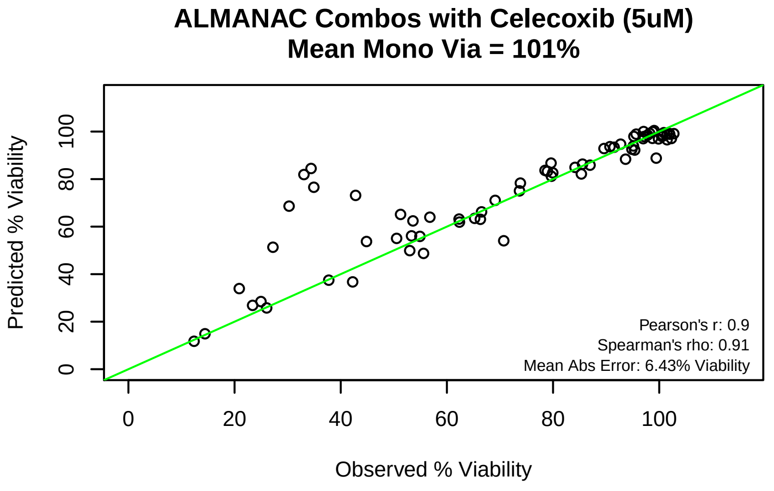
ALMANAC Combos with Celecoxib (5uM)
Mean Mono Via = 101%
Predicted % Viability
Observed % Viability
Pearson's r: 0.9
Spearman's rho: 0.91
Mean Abs Error: 6.43% Viability

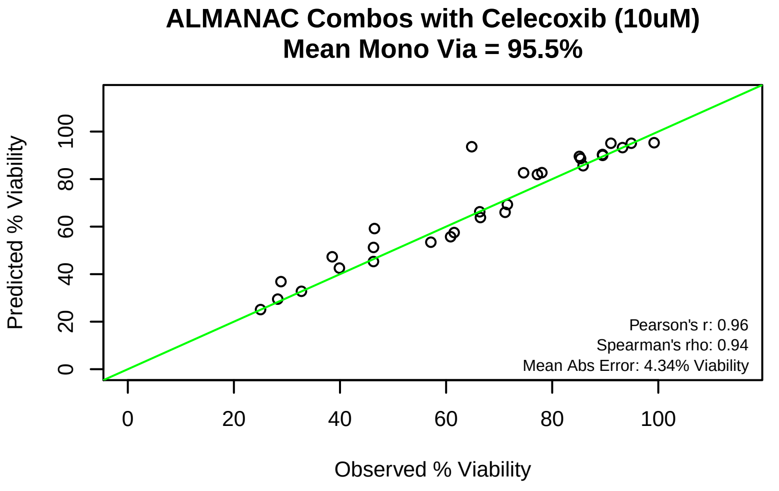
ALMANAC Combos with Celecoxib (10uM)
Mean Mono Via = 95.5%
Predicted % Viability
Observed % Viability
Pearson's r: 0.96
Spearman's rho: 0.94
Mean Abs Error: 4.34% Viability

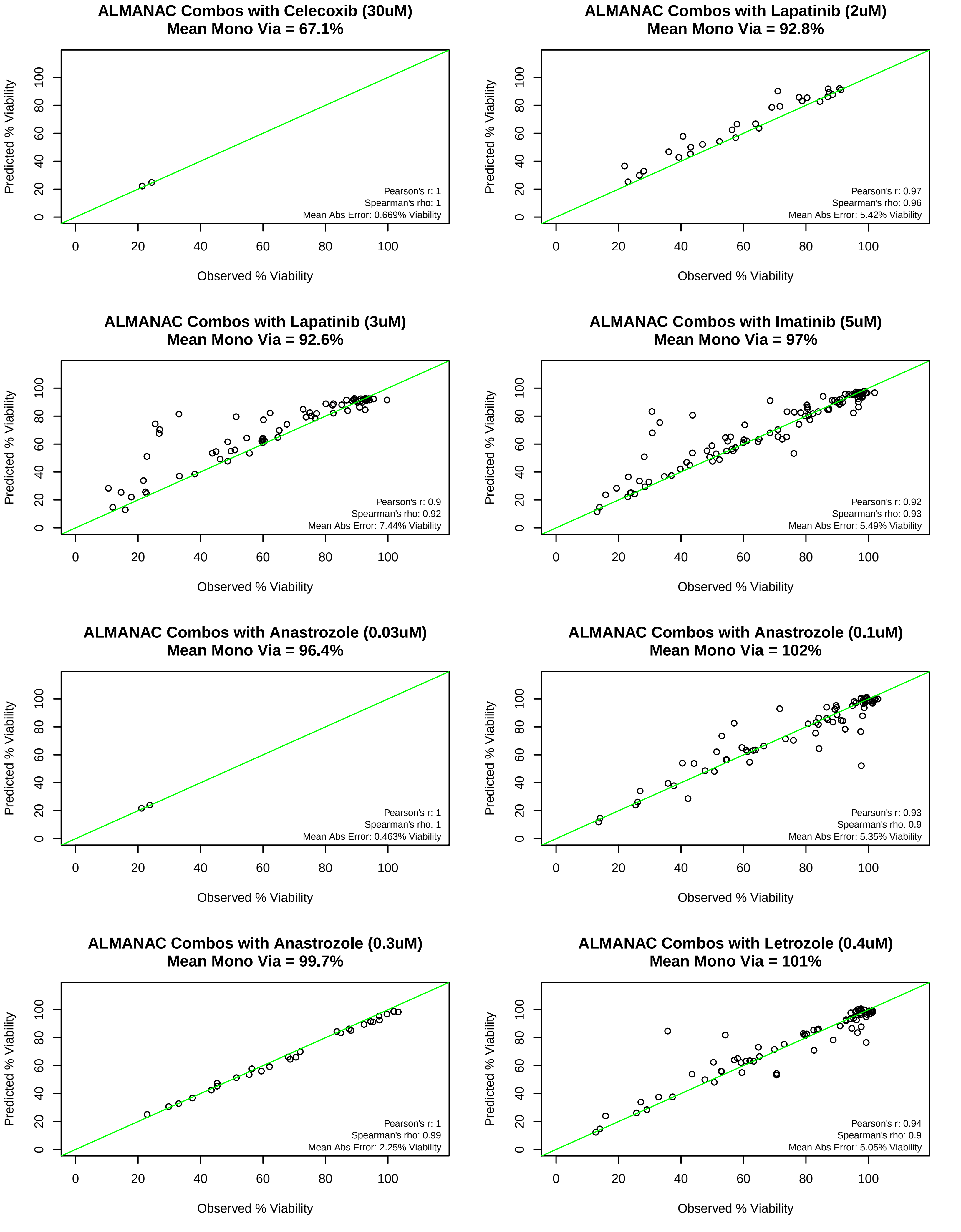
ALMANAC Combos with Celecoxib (30uM)
Mean Mono Via = 67.1%
Pearson's r: 1
Spearman's rho: 1
Mean Abs Error: 0.669% Viability
ALMANAC Combos with Lapatinib (2uM)
Mean Mono Via = 92.8%
Pearson's r: 0.97
Spearman's rho: 0.96
Mean Abs Error: 5.42% Viability
ALMANAC Combos with Lapatinib (3uM)
Mean Mono Via = 92.6%
Pearson's r: 0.9
Spearman's rho: 0.92
Mean Abs Error: 7.44% Viability
ALMANAC Combos with Imatinib (5uM)
Mean Mono Via = 97%
Pearson's r: 0.92
Spearman's rho: 0.93
Mean Abs Error: 5.49% Viability
ALMANAC Combos with Anastrozole (0.03uM)
Mean Mono Via = 96.4%
Pearson's r: 1
Spearman's rho: 1
Mean Abs Error: 0.463% Viability
ALMANAC Combos with Anastrozole (0.1uM)
Mean Mono Via = 102%
Pearson's r: 0.93
Spearman's rho: 0.9
Mean Abs Error: 5.35% Viability
ALMANAC Combos with Anastrozole (0.3uM)
Mean Mono Via = 99.7%
Pearson's r: 1
Spearman's rho: 0.99
Mean Abs Error: 2.25% Viability
ALMANAC Combos with Letrozole (0.4uM)
Mean Mono Via = 101%
Pearson's r: 0.94
Spearman's rho: 0.9
Mean Abs Error: 5.05% Viability
Predicted % Viability
Observed % Viability
0
20
40
60
80
100

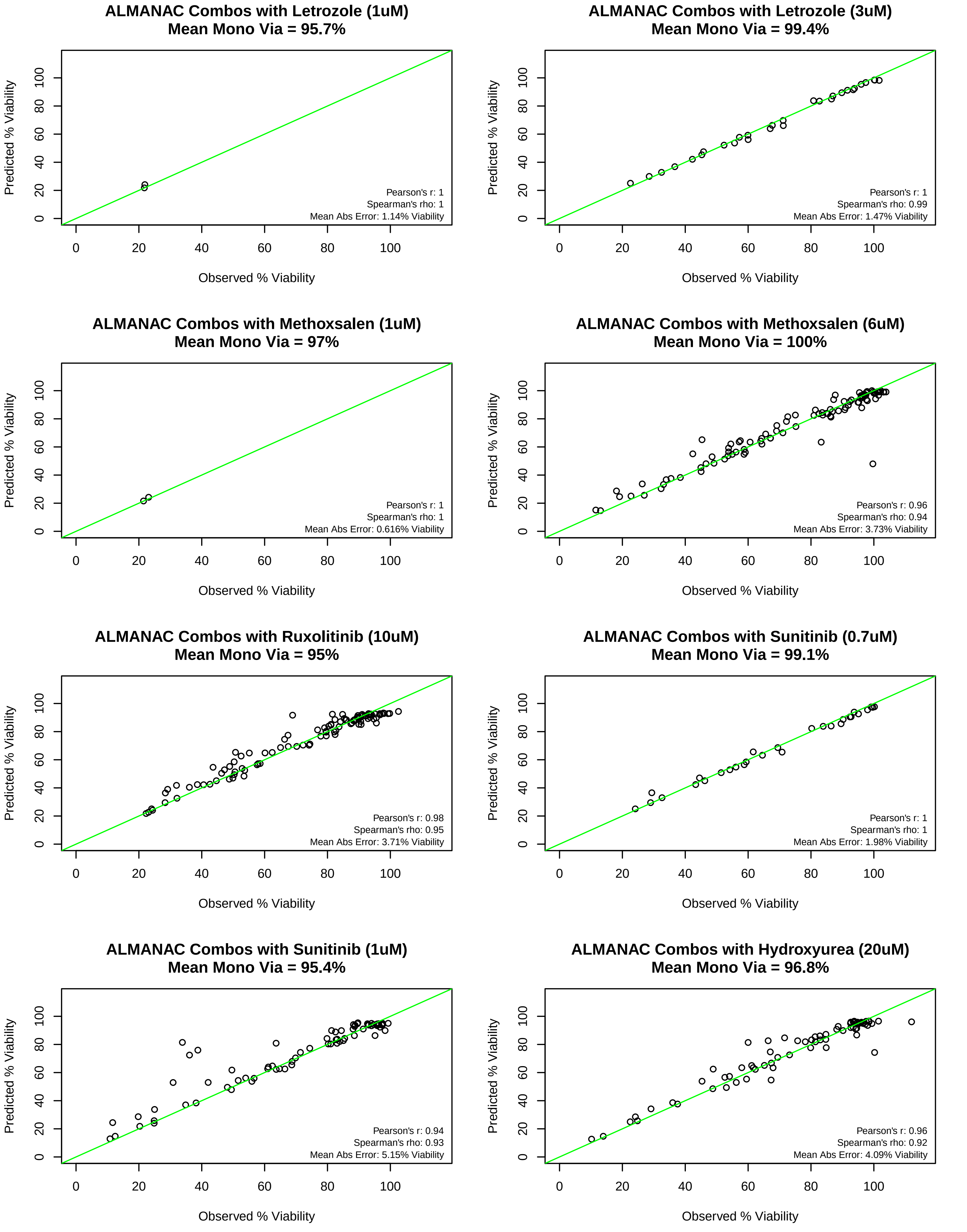
ALMANAC Combos with Letrozole (1uM)
Mean Mono Via = 95.7%
Pearson's r: 1
Spearman's rho: 1
Mean Abs Error: 1.14% Viability
ALMANAC Combos with Letrozole (3uM)
Mean Mono Via = 99.4%
Pearson's r: 1
Spearman's rho: 0.99
Mean Abs Error: 1.47% Viability
ALMANAC Combos with Methoxsalen (1uM)
Mean Mono Via = 97%
Pearson's r: 1
Spearman's rho: 1
Mean Abs Error: 0.616% Viability
ALMANAC Combos with Methoxsalen (6uM)
Mean Mono Via = 100%
Pearson's r: 0.96
Spearman's rho: 0.94
Mean Abs Error: 3.73% Viability
ALMANAC Combos with Ruxolitinib (10uM)
Mean Mono Via = 95%
Pearson's r: 0.98
Spearman's rho: 0.95
Mean Abs Error: 3.71% Viability
ALMANAC Combos with Sunitinib (0.7uM)
Mean Mono Via = 99.1%
Pearson's r: 1
Spearman's rho: 1
Mean Abs Error: 1.98% Viability
ALMANAC Combos with Sunitinib (1uM)
Mean Mono Via = 95.4%
Pearson's r: 0.94
Spearman's rho: 0.93
Mean Abs Error: 5.15% Viability
ALMANAC Combos with Hydroxyurea (20uM)
Mean Mono Via = 96.8%
Pearson's r: 0.96
Spearman's rho: 0.92
Mean Abs Error: 4.09% Viability
Predicted % Viability
Observed % Viability
0
20
40
60
80
100

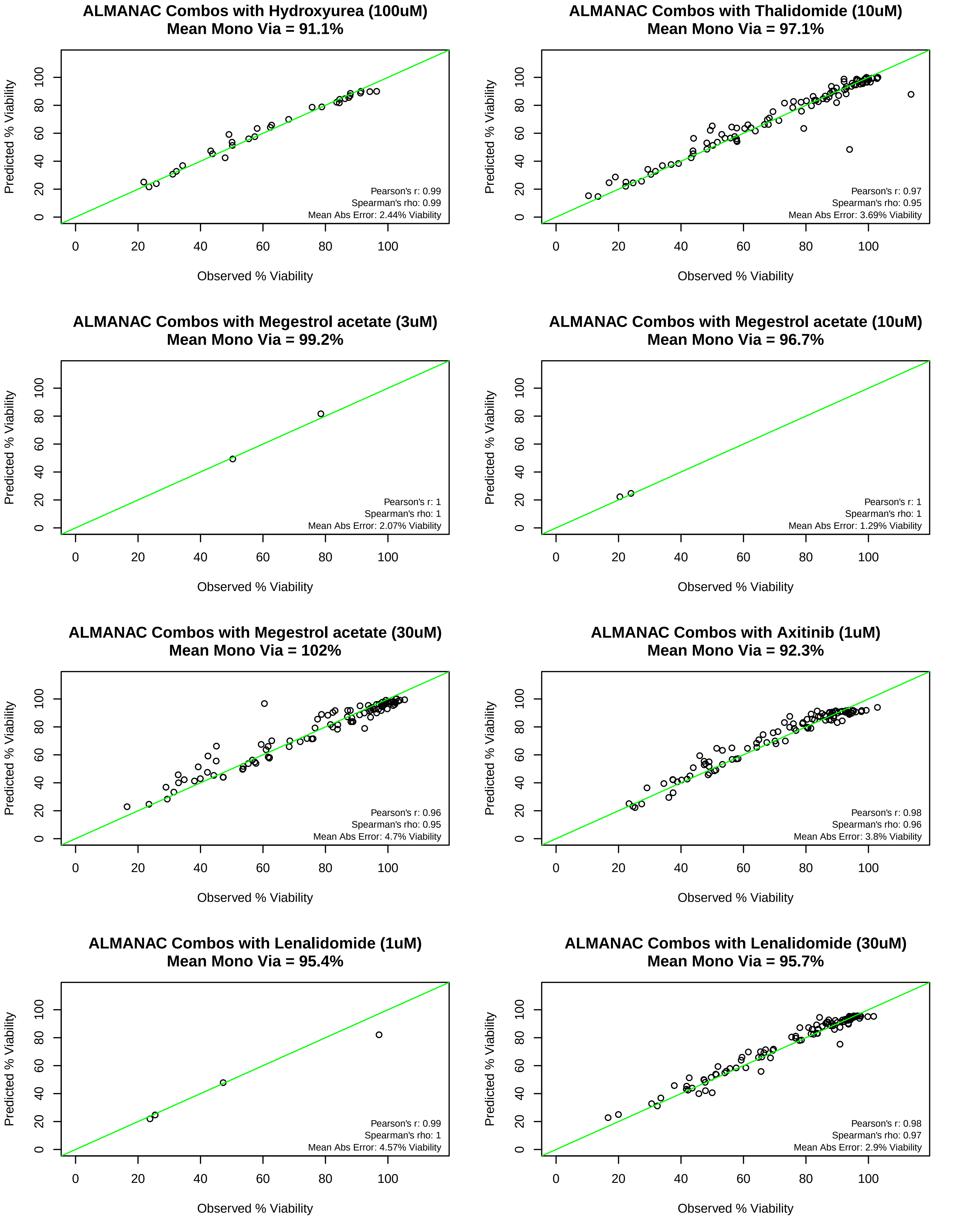
ALMANAC Combos with Hydroxyurea (100uM)
Mean Mono Via = 91.1%
Pearson's r: 0.99
Spearman's rho: 0.99
Mean Abs Error: 2.44% Viability
ALMANAC Combos with Thalidomide (10uM)
Mean Mono Via = 97.1%
Pearson's r: 0.97
Spearman's rho: 0.95
Mean Abs Error: 3.69% Viability
ALMANAC Combos with Megestrol acetate (3uM)
Mean Mono Via = 99.2%
Pearson's r: 1
Spearman's rho: 1
Mean Abs Error: 2.07% Viability
ALMANAC Combos with Megestrol acetate (10uM)
Mean Mono Via = 96.7%
Pearson's r: 1
Spearman's rho: 1
Mean Abs Error: 1.29% Viability
ALMANAC Combos with Megestrol acetate (30uM)
Mean Mono Via = 102%
Pearson's r: 0.96
Spearman's rho: 0.95
Mean Abs Error: 4.7% Viability
ALMANAC Combos with Axitinib (1uM)
Mean Mono Via = 92.3%
Pearson's r: 0.98
Spearman's rho: 0.96
Mean Abs Error: 3.8% Viability
ALMANAC Combos with Lenalidomide (1uM)
Mean Mono Via = 95.4%
Pearson's r: 0.99
Spearman's rho: 1
Mean Abs Error: 4.57% Viability
ALMANAC Combos with Lenalidomide (30uM)
Mean Mono Via = 95.7%
Pearson's r: 0.98
Spearman's rho: 0.97
Mean Abs Error: 2.9% Viability
Predicted % Viability
Observed % Viability
0
20
40
60
80
100

ALMANAC Combos with Allopurinol (10uM)
Mean Mono Via = 98.1%

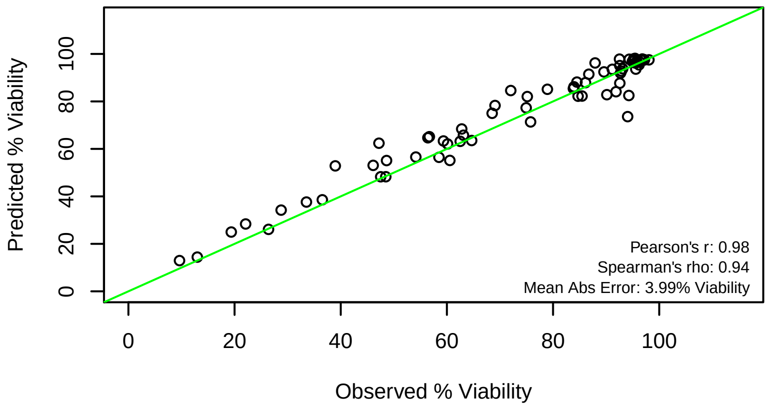

ALMANAC Combos with Allopurinol (100uM)
Mean Mono Via = 90.6%

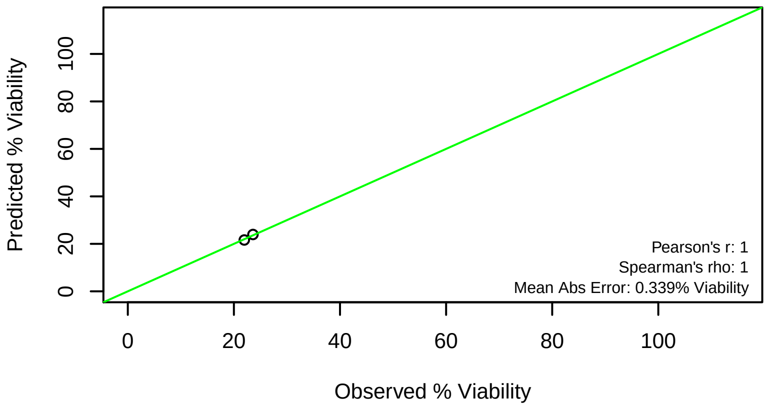

ALMANAC Combos with Allopurinol (200uM)
Mean Mono Via = 93%

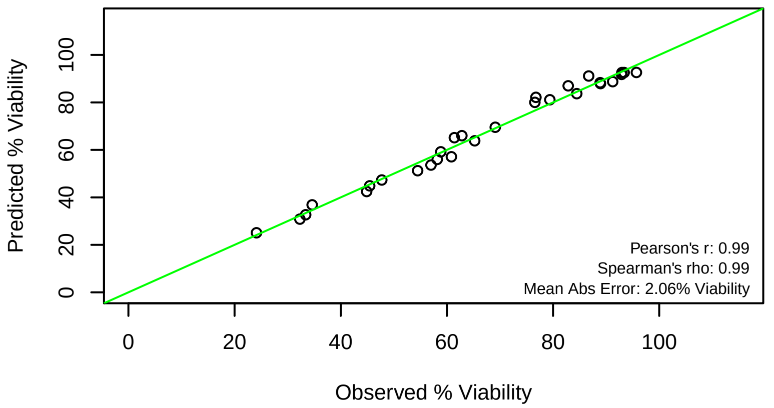

ALMANAC Combos with Decitabine (0.3uM)
Mean Mono Via = 86.1%

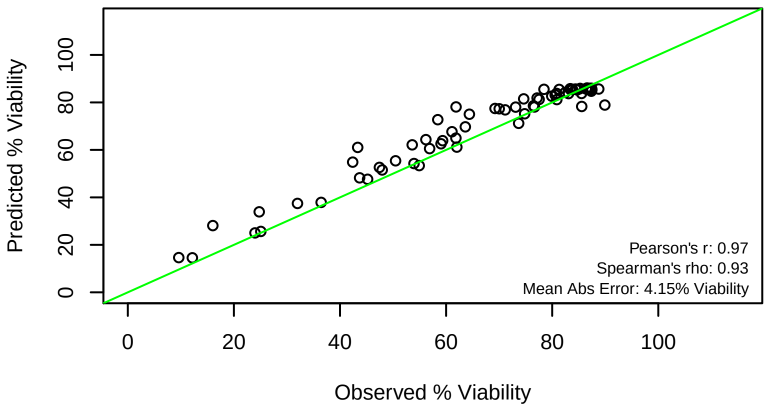

ALMANAC Combos with Decitabine (1uM)
Mean Mono Via = 80.4%

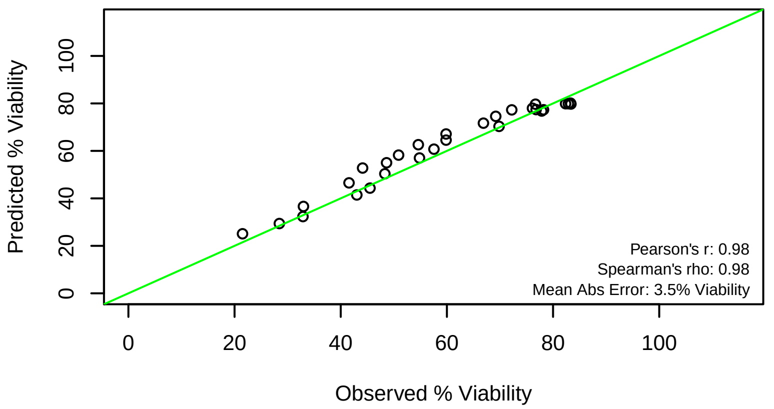

ALMANAC Combos with Decitabine (3uM)
Mean Mono Via = 89.6%

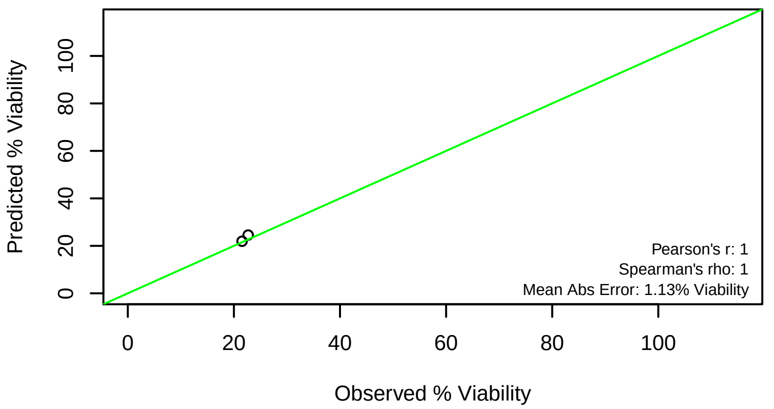

ALMANAC Combos with Erlotinib (2uM)
Mean Mono Via = 87.6%

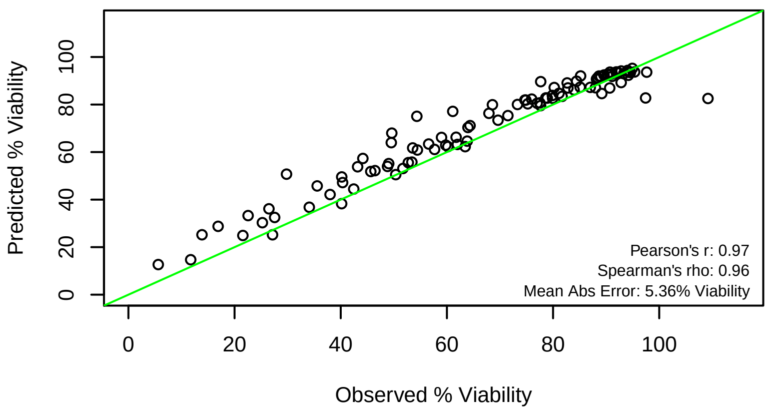

ALMANAC Combos with Erlotinib (10uM)
Mean Mono Via = 66.5%

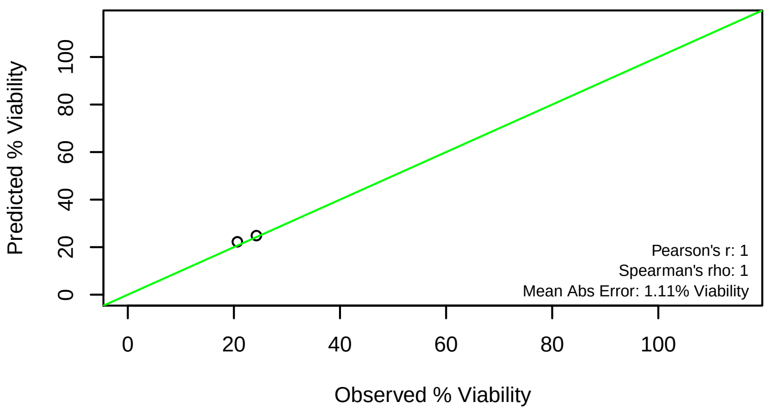

ALMANAC Combos with Raloxifene hydrochloride (0.01uM)
Mean Mono Via = 99.9%

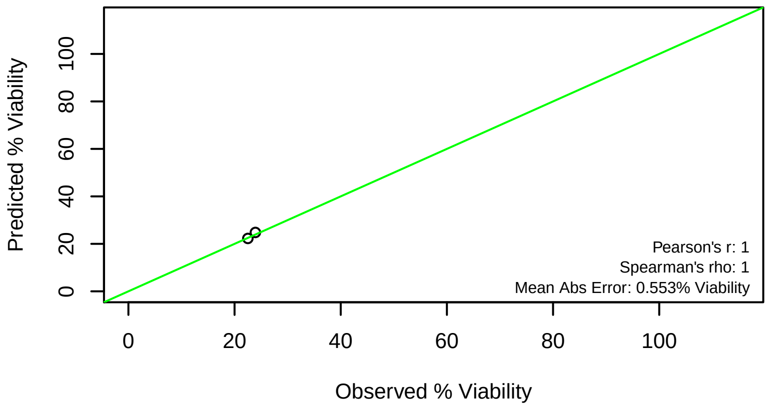

ALMANAC Combos with Raloxifene hydrochloride (0.1uM)
Mean Mono Via = 100%

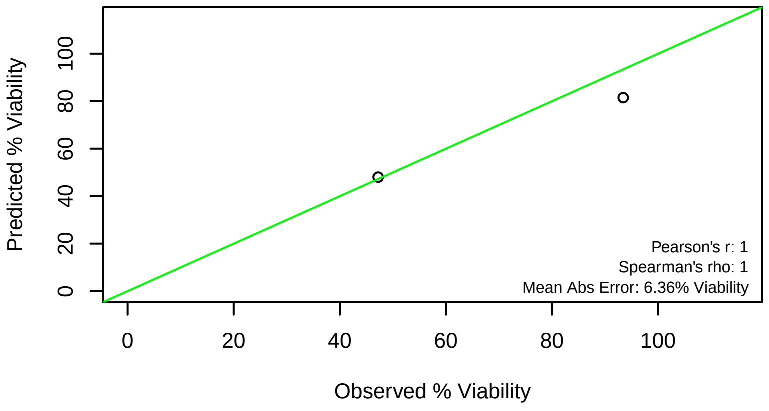

ALMANAC Combos with Raloxifene hydrochloride (1uM)
Mean Mono Via = 101%

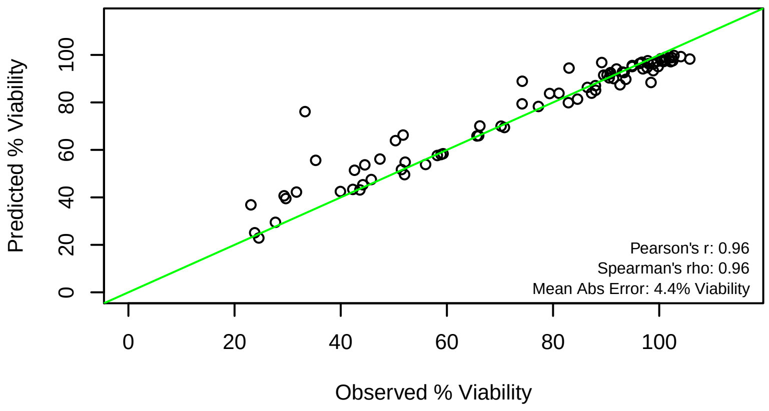

ALMANAC Combos with Raloxifene hydrochloride (30uM)
Mean Mono Via = 33.1%

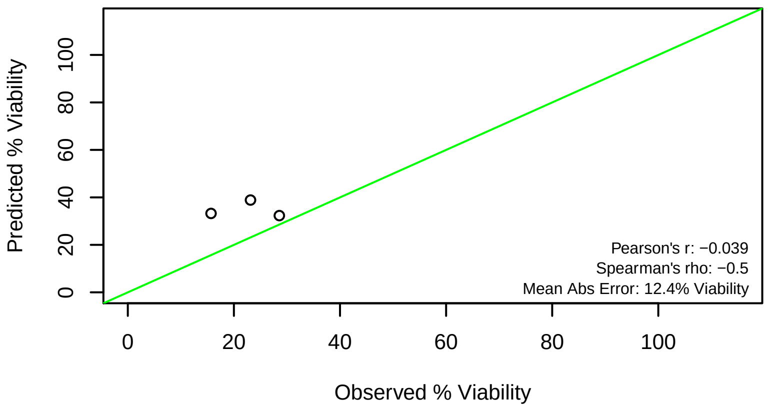

ALMANAC Combos with Altretamine (0.01uM)
Mean Mono Via = 98.7%

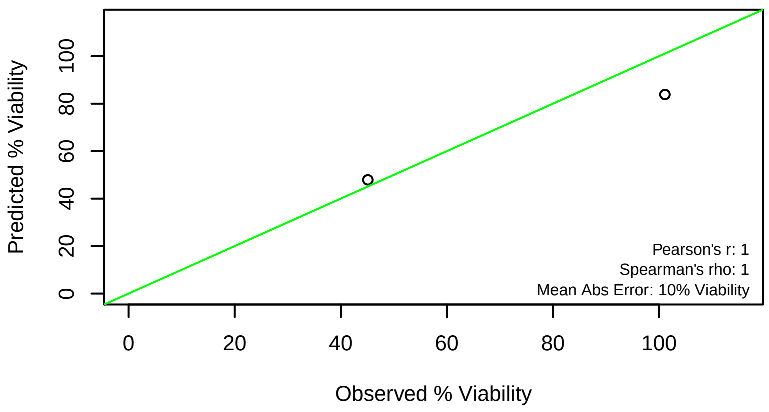

ALMANAC Combos with Altretamine (3uM)
Mean Mono Via = 101%

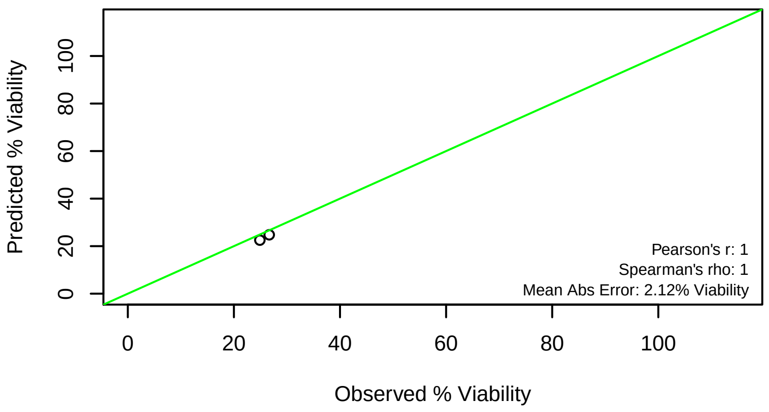

ALMANAC Combos with Altretamine (75uM)
Mean Mono Via = 101%

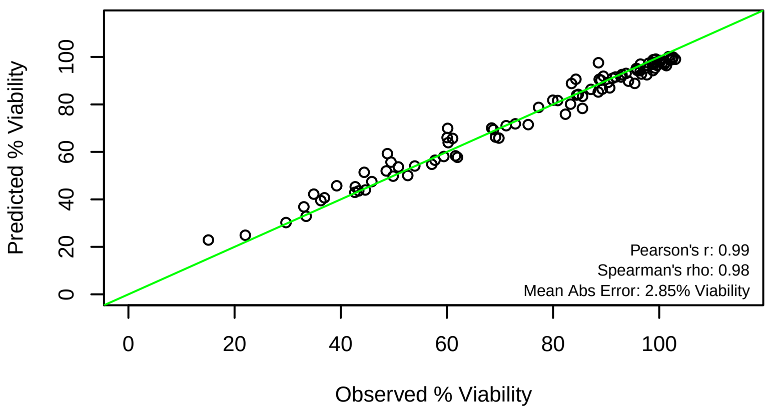

ALMANAC Combos with Ifosfamide (100uM)
Mean Mono Via = 98.6%

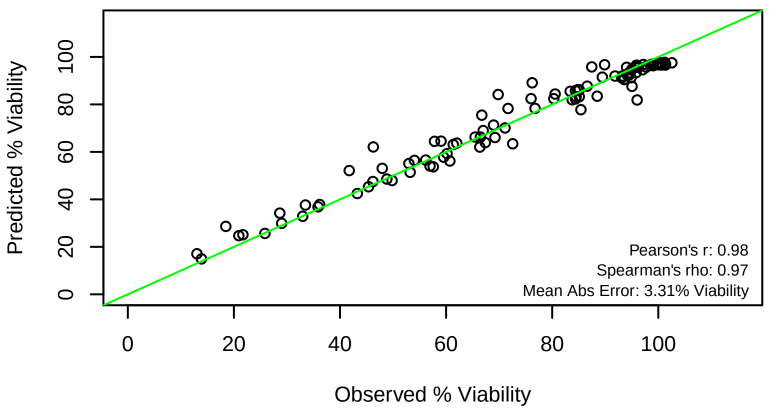

ALMANAC Combos with Ifosfamide (400uM)
Mean Mono Via = 94.9%

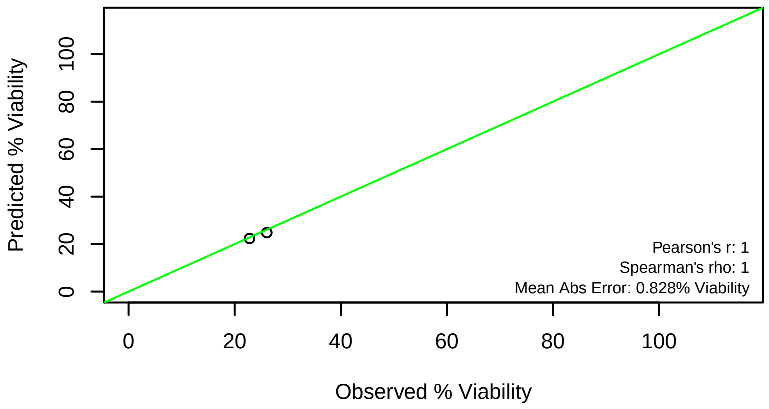

ALMANAC Combos with Imiquimod (0.07uM)
Mean Mono Via = 100%

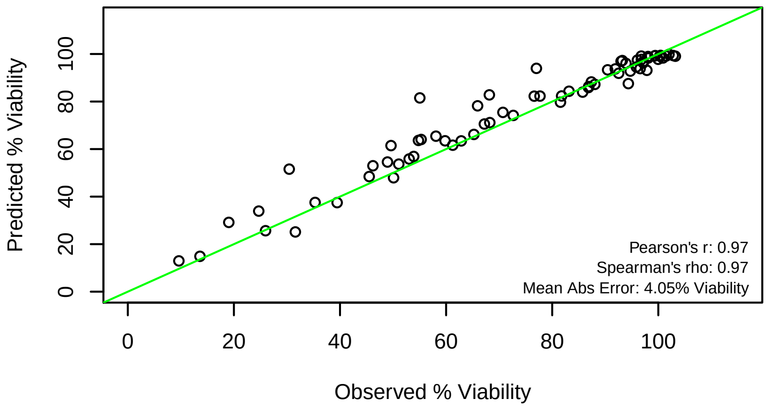

ALMANAC Combos with Imiquimod (0.5uM)
Mean Mono Via = 101%

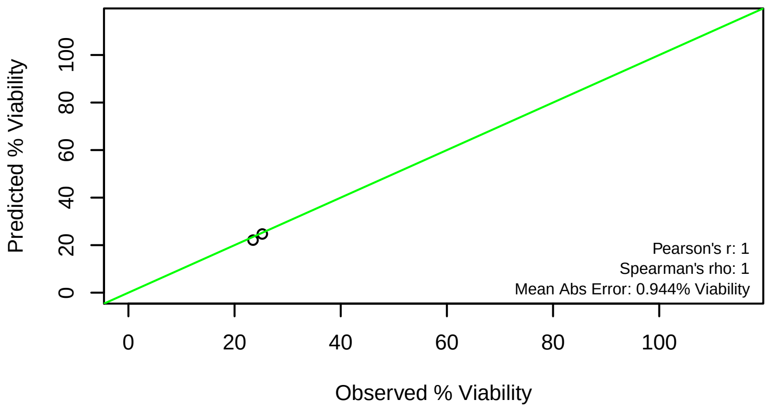

ALMANAC Combos with Imiquimod (0.7uM)
Mean Mono Via = 102%

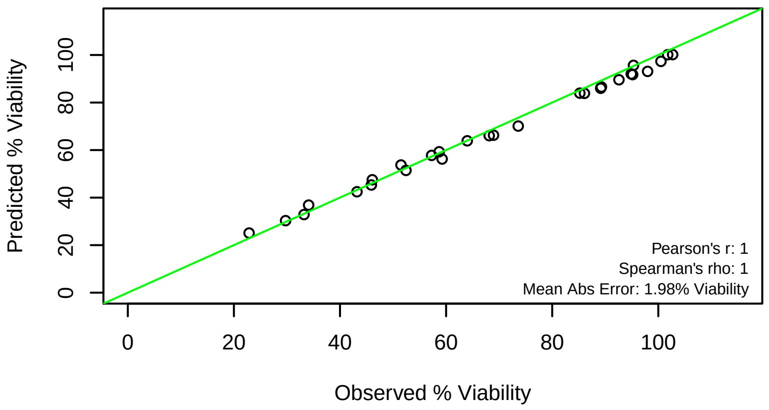

ALMANAC Combos with Eribulin mesylate (0.01uM)
Mean Mono Via = 57.2%

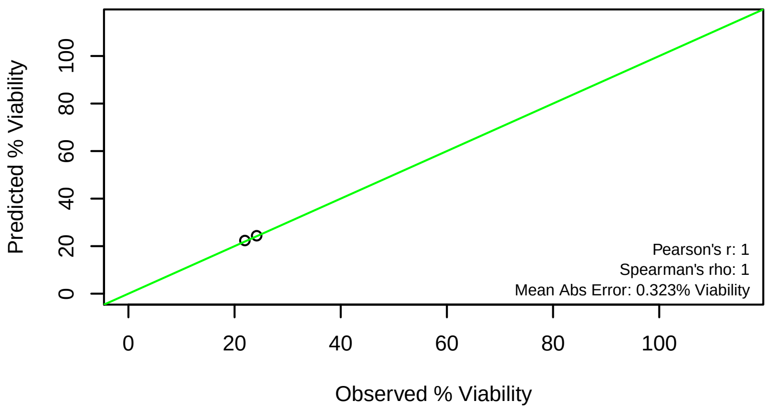

Supplement: Supplementary file 5 — Supplementary Data 2 [file 41467_2020_19563_MOESM5_ESM.zip › Supplementary Data 2_drug by drug NCI-ALMANAC validation.pdf]
