# Supplementary material for: Computationally predicting clinical drug combination efficacy with cancer cell line screens and independent drug action
Source: Nat Commun. 2020 Nov 17;11:5848. doi: 10.1038/s41467-020-19563-6 (PMC7673995; doi:10.1038/s41467-020-19563-6)
Supplement: Supplementary file 10 — Reporting Summary [file 41467_2020_19563_MOESM10_ESM.pdf]

nature research

Corresponding author(s): R. Stephanie Huang

Last updated by author(s): Oct 7, 2020

Reporting Summary

Nature Research wishes to improve the reproducibility of the work that we publish. This form provides structure for consistency and transparency in reporting. For further information on Nature Research policies, see our Editorial Policies and the Editorial Policy Checklist.

Statistics

For all statistical analyses, confirm that the following items are present in the figure legend, table legend, main text, or Methods section.

n/a	Confirmed	
☐	☒	The exact sample size (n) for each experimental group/condition, given as a discrete number and unit of measurement
☒	☐	A statement on whether measurements were taken from distinct samples or whether the same sample was measured repeatedly
☐	☒	The statistical test(s) used AND whether they are one- or two-sided<br>*Only common tests should be described solely by name; describe more complex techniques in the Methods section.*
☒	☐	A description of all covariates tested
☐	☒	A description of any assumptions or corrections, such as tests of normality and adjustment for multiple comparisons
☐	☒	A full description of the statistical parameters including central tendency (e.g. means) or other basic estimates (e.g. regression coefficient) AND variation (e.g. standard deviation) or associated estimates of uncertainty (e.g. confidence intervals)
☐	☒	For null hypothesis testing, the test statistic (e.g. F, t, r) with confidence intervals, effect sizes, degrees of freedom and P value noted<br>*Give P values as exact values whenever suitable.*
☒	☐	For Bayesian analysis, information on the choice of priors and Markov chain Monte Carlo settings
☒	☐	For hierarchical and complex designs, identification of the appropriate level for tests and full reporting of outcomes
☐	☒	Estimates of effect sizes (e.g. Cohen's d, Pearson's r), indicating how they were calculated

Our web collection on statistics for biologists contains articles on many of the points above.

Software and code

Policy information about availability of computer code

Data collection	The code necessary to compile the clinical trial results dataset used in our analysis, along with detailed readme files to aid investigators in navigating and understanding each analysis and script in the project, have been uploaded in their entirety to Open Science Framework (OSF) and are stored in the "IDACombo Paper" project, which can be publicly accessed at https://osf.io/sym6h/. Specifically, the code for compiling the clinical trial result dataset are located within the "Identifying Clinical Trials" folder of the OSF repository and involved use of the rvest R package (v0.3.2, https://CRAN.R-project.org/package=rvest).
Data analysis	The code necessary to reproduce the analyses in this paper, along with detailed readme files to aid investigators in navigating and understanding each analysis and script in the project, have been uploaded in their entirety to Open Science Framework (OSF) and are stored in the "IDACombo Paper" project, which can be publicly accessed at https://osf.io/sym6h/.<br><br>Most analyses were performed using R v3.4.229 with Microsoft R Open v3.4.230 and RStudio v1.1.46331. Processing of the raw dose-response data from CTRPv2 and GDSC was performed using the Mesabi compute cluster at the Minnesota Supercomputing Institute (MSI) at the University of Minnesota (http://www.msi.umn.edu) and R v3.4.4.<br><br>The IDACombo R package (v1.0.2) created for this analysis is available on GitHub at https://github.com/Alexander-Ling/IDACombo/.<br>Additional R packages used in the analysis are listed below:<br>1. car (v2.1.5, https://CRAN.R-project.org/package=car)<br>2. ComplexHeatmap (v1.14.0, https://bioconductor.org/packages/release/bioc/html/ComplexHeatmap.html)<br>3. drc (v3.0.1, https://CRAN.R-project.org/package=drc)<br>4. openxlsx (v4.1.4, https://CRAN.R-project.org/package=openxlsx)<br>5. parallel (v3.4.2, Created by the R Core team and included in R since R version 2.14.0.)<br>6. pbapply (v1.3.3, https://CRAN.R-project.org/package=pbapply)

7. powerSurvEpi (v0.0.9, https://CRAN.R-project.org/package=powerSurvEpi)
8. precrec (v0.9.1, https://CRAN.R-project.org/package=precrec)
9. progress (v1.1.2, https://CRAN.R-project.org/package=progress)
10. RColorBrewer (v1.1.2, https://CRAN.R-project.org/package=RColorBrewer)
11. readr (v1.1.1, https://CRAN.R-project.org/package=readr)
12. readxl (v1.0.0, https://CRAN.R-project.org/package=readxl)
13. rgl (v0.98.1, https://CRAN.R-project.org/package=rgl)
14. rvest (v0.3.2, https://CRAN.R-project.org/package=rvest)
15. sandwich (v2.4.0, https://CRAN.R-project.org/package=sandwich)
16. xlsx (v0.5.7, https://CRAN.R-project.org/package=xlsx)

For manuscripts utilizing custom algorithms or software that are central to the research but not yet described in published literature, software must be made available to editors and reviewers. We strongly encourage code deposition in a community repository (e.g. GitHub). See the Nature Research guidelines for submitting code & software for further information.

Data

Policy information about availability of data

All manuscripts must include a data availability statement. This statement should provide the following information, where applicable:

- Accession codes, unique identifiers, or web links for publicly available datasets
- A list of figures that have associated raw data
- A description of any restrictions on data availability

The data necessary to reproduce the analyses in this paper, along with detailed readme files to aid investigators in navigating and understanding each analysis and script in the project, have been uploaded in their entirety (with the exception of the AstraZeneca-Sanger Drug Combination Prediction DREAM Challenge dataset, see below) to Open Science Framework (OSF) and are stored in the "IDACombo Paper" project, which can be publicly accessed at https://osf.io/sym6h/.

The NCI-ALMANAC dataset was accessed by downloading the "ComboDrugGrowth_Nov2017.zip" folder from the following link on 5/17/2019: https://wiki.nci.nih.gov/download/attachments/338237347/ComboDrugGrowth_Nov2017.zip?version=1&modificationDate=1510057275000&api=v2

The AstraZeneca-Sanger Drug Combination Prediction DREAM Challenge dataset was accessed by downloading the "DREAM_OI_matrices_final.zip" [syn18468836] and "OI_combinations_synergy_scores_final.txt" [syn18435126] files from Synapse.org on 2/13/2020. Note that this data is not included in the OSF repository for this paper because it is controlled access and cannot be distributed with this manuscript.

The O'Neil et al., 2016 dataset was obtained by downloading the supplemental data files associated with O'Neil et al.7 using links from the following webpage on 1/31/2020: https://mct.aacrjournals.org/content/15/6/1155.figures-only

Release 6.0 of the GDSC dataset was downloaded from the following website on 2/26/2018: ftp://ftp.sanger.ac.uk/pub4/cancerrxgene/releases/release-6.0

The CTRPv2 dataset was downloaded from the following weblink on 2/26/2018: ftp://anonymous:guest@caftpd.nci.nih.gov/pub/OCG-DCC/CTD2/Broad/CTRPv2.0_2015_ctd2_ExpandedDataset/CTRPv2.0_2015_ctd2_ExpandedDataset.zip

Field-specific reporting

Please select the one below that is the best fit for your research. If you are not sure, read the appropriate sections before making your selection.

[x] Life sciences [ ] Behavioural & social sciences [ ] Ecological, evolutionary & environmental sciences

For a reference copy of the document with all sections, see nature.com/documents/nr-reporting-summary-flat.pdf

Life sciences study design

All studies must disclose on these points even when the disclosure is negative.

Sample size	Sample sizes for the number of cell lines required to make drug combination efficacy predictions were usually defined by the number of available cell lines in the published datasets which we use to make predictions. We performed analyses reported in Figure S10 establishing the suitability of these sample sizes and speculating on minimum acceptable sample sizes for disease specific predictions in the future.<br><br>Sample sizes for the number of clinical trials included in the clinical trial validation portion of our paper were entirely based on the number of available published trials suitable for our analysis. This availability was determined by systematically querying clinicaltrials.gov using a mix of computational and manual curation to identify all available trials which met our inclusion criteria. Those criteria are described in Figure 3 and in the "Data exclusions" section below. The suitability of those sample sizes is evidenced by the statistically highly significant differences found between trial groups.<br><br>Patient sample sizes for each clinical trial were determined by the authors of those studies.
Data exclusions	Clinical trials were excluded from our validation analysis if they did not meet any of the following inclusion criteria:<br>1. Completed, phase III clinical trial

	2. ≥50 patients per trial arm<br>3. All cytotoxic drugs in control and test therapies are available in at least one of either CTRPv2 or GDSC<br>4. ≥50 cell lines available for predictions of tested control and test therapies<br>5. Test therapy is control therapy plus one or more additional drugs<br>6. Clinically relevant drug concentrations for each drug in a trial are not > 2x the tested drug concentrations in the dataset(s) necessary to predict that trial's efficacy (i.e. CTRPv2 and/or GDSC)<br>7. Trial is not substantially the same as another selected trial (i.e. same treatment groups, doses, cancer type, patient population, and outcomes).<br><br>These criteria were established prospectively with the exception of the criteria that clinically relevant drug concentrations for each drug in a trial must not be > 2x the tested drug concentrations in the in vitro dataset(s) used to make predictions for that trial as this was not a problem we were expecting when the criteria were initially defined.
Replication	Given the limited number of clinical trials available for this dataset, no meaningful replication analyses were possible. Seeds are defined at the beginning of each script to ensure that the outputs of those scripts are exactly replicated if they are rerun.
Randomization	This is not relevant to our study, because we do not report any experiments in which we divide a population of patients or cell lines into groups and then test the effect of different treatments or analysis techniques between groups.
Blinding	The only analyses for which blinding would be relevant in our study is the clinical trial validation analyses, where authors could have been blinded to published trial outcomes prior to making trial outcome predictions. This was not possible, because the authors had to first create the clinical dataset before predictions could be made. It was also not necessary, because all clinical trial power predictions were made using a well-defined pipeline that was consistently applied across all trials. Furthermore, the only variable that authors could influence to affect prediction outcomes was the selected drug concentration believed to be most clinically relevant. The definition for these concentrations was also well defined and applied consistently, and selected drug concentrations affected predictions for all trials which used each drug regardless of reported trial outcomes.

Reporting for specific materials, systems and methods

We require information from authors about some types of materials, experimental systems and methods used in many studies. Here, indicate whether each material, system or method listed is relevant to your study. If you are not sure if a list item applies to your research, read the appropriate section before selecting a response.

Materials & experimental systems

n/a	Involved in the study
☒	☐ Antibodies
☒	☐ Eukaryotic cell lines
☒	☐ Palaeontology and archaeology
☒	☐ Animals and other organisms
☒	☐ Human research participants
☐	☒ Clinical data
☒	☐ Dual use research of concern

Methods

n/a	Involved in the study
☒	☐ ChIP-seq
☒	☐ Flow cytometry
☒	☐ MRI-based neuroimaging

Clinical data

Policy information about clinical studies

All manuscripts should comply with the ICMJEguidelines for publication of clinical research and a completedCONSORT checklist must be included with all submissions.

Clinical trial registration	All of the clinical data used in this paper was obtained from previously published clinical trials. As such, clinical trial registration information can be found in the publications for each trial, which are listed in the paper. Clinical trial registration numbers from clinicaltrials.gov are also available for each trial in Data S4.
Study protocol	All of the clinical data used in this paper was obtained from previously published clinical trials. As such, clinical trial registration information can be found in the publications for each trial, which are listed in the paper. Clinical trial registration numbers from clinicaltrials.gov are also available for each trial in Data S4.
Data collection	All of the clinical data used in this paper was obtained from previously published clinical trials. As such, clinical trial registration information can be found in the publications for each trial, which are listed in the paper. Clinical trial registration numbers from clinicaltrials.gov are also available for each trial in Data S4.
Outcomes	All of the clinical data used in this paper was obtained from previously published clinical trials. As such, clinical trial registration information can be found in the publications for each trial, which are listed in the paper. Clinical trial registration numbers from clinicaltrials.gov are also available for each trial in Data S4.
